# Quality and Dependability of ChatGPT and DingXiangYuan Forums for Remote Orthopedic Consultations: Comparative Analysis

**DOI:** 10.2196/50882

**Published:** 2024-03-14

**Authors:** Zhaowen Xue, Yiming Zhang, Wenyi Gan, Huajun Wang, Guorong She, Xiaofei Zheng

**Affiliations:** 1 Department of Bone and Joint Surgery and Sports Medicine Center, The First Affiliated Hospital, The First Affiliated Hospital of Jinan University Guangzhou China

**Keywords:** artificial intelligence, ChatGPT, consultation, musculoskeletal, natural language processing, remote medical consultation, orthopaedic, orthopaedics

## Abstract

**Background:**

The widespread use of artificial intelligence, such as ChatGPT (OpenAI), is transforming sectors, including health care, while separate advancements of the internet have enabled platforms such as China’s DingXiangYuan to offer remote medical services.

**Objective:**

This study evaluates ChatGPT-4’s responses against those of professional health care providers in telemedicine, assessing artificial intelligence’s capability to support the surge in remote medical consultations and its impact on health care delivery.

**Methods:**

We sourced remote orthopedic consultations from “Doctor DingXiang,” with responses from its certified physicians as the control and ChatGPT’s responses as the experimental group. In all, 3 blindfolded, experienced orthopedic surgeons assessed responses against 7 criteria: “logical reasoning,” “internal information,” “external information,” “guiding function,” “therapeutic effect,” “medical knowledge popularization education,” and “overall satisfaction.” We used Fleiss κ to measure agreement among multiple raters.

**Results:**

Initially, consultation records for a cumulative count of 8 maladies (equivalent to 800 cases) were gathered. We ultimately included 73 consultation records by May 2023, following primary and rescreening, in which no communication records containing private information, images, or voice messages were transmitted. After statistical scoring, we discovered that ChatGPT’s “internal information” score (mean 4.61, SD 0.52 points vs mean 4.66, SD 0.49 points; *P*=.43) and “therapeutic effect” score (mean 4.43, SD 0.75 points vs mean 4.55, SD 0.62 points; *P*=.32) were lower than those of the control group, but the differences were not statistically significant. ChatGPT showed better performance with a higher “logical reasoning” score (mean 4.81, SD 0.36 points vs mean 4.75, SD 0.39 points; *P*=.38), “external information” score (mean 4.06, SD 0.72 points vs mean 3.92, SD 0.77 points; *P*=.25), and “guiding function” score (mean 4.73, SD 0.51 points vs mean 4.72, SD 0.54 points; *P*=.96), although the differences were not statistically significant. Meanwhile, the “medical knowledge popularization education” score of ChatGPT was better than that of the control group (mean 4.49, SD 0.67 points vs mean 3.87, SD 1.01 points; *P*<.001), and the difference was statistically significant. In terms of “overall satisfaction,” the difference was not statistically significant between the groups (mean 8.35, SD 1.38 points vs mean 8.37, SD 1.24 points; *P*=.92). According to how Fleiss κ values were interpreted, 6 of the control group’s score points were classified as displaying “fair agreement” (*P*<.001), and 1 was classified as showing “substantial agreement” (*P*<.001). In the experimental group, 3 points were classified as indicating “fair agreement,” while 4 suggested “moderate agreement” (*P*<.001).

**Conclusions:**

ChatGPT-4 matches the expertise found in DingXiangYuan forums’ paid consultations, excelling particularly in scientific education. It presents a promising alternative for remote health advice. For health care professionals, it could act as an aid in patient education, while patients may use it as a convenient tool for health inquiries.

## Introduction

The fast growth of artificial intelligence (AI) in recent years has brought tremendous changes to different professions and businesses, altering the way people live and work. The application of AI in medicine is expanding in several areas, including medical image analysis, medication-interaction detection, the identification of high-risk patients, and medical record coding [1,2]. As technology advances, OpenAI introduced ChatGPT on November 30, 2022, as a new kind of natural language model capable of communicating with people through text-to-text, human-like dialogues [3,4]. The more powerful GPT-4 subsequently became accessible through a paid ChatGPT Plus membership on March 13, 2023. It has attracted a lot of interest since its release and has the potential to be widely used in the health care system [5,6]. Most medical AI research has targeted medical workers as software users, which requires medical knowledge reserves [7]. ChatGPT and other conversation question-and-answer AI software programs do not establish a user threshold, and their strong function makes them an essential auxiliary tool to increase finance and management job efficiency [8]. Health is a natural component of humans and should be explored and used in ChatGPT, particularly in the context of situational conversations between patients and physicians.

As human civilization advances, the quest for more convenient, professional, and precise medical services intensifies, with patients expecting increasingly high standards of care. The internet era has spurred hospitals to offer remote diagnostic and treatment services, facilitating doctor-patient interactions beyond physical boundaries and enhancing an understanding of medical issues through remote health care, particularly for those far from medical centers [1]. The recent COVID-19 pandemic has accelerated this digital shift in medicine [2,9,10]. However, the complexity of medical information can reduce physician efficiency and patient comprehension, highlighting the need for patient navigation services, especially in countries with evolving medical systems such as China [11-13]. Amid this backdrop, the rapid advancement of AI technologies such as ChatGPT offers promising support in navigating medical systems, aiding patients in understanding their disease, and selecting a health care facility [14].

“DingXiangYuan” is a leading digital health technology enterprise in China that seeks to unite physicians, researchers, patients, and hospitals through expert and authoritative knowledge exchange, extensive and thorough medical data collection, and top-notch digital medical services [15]. Its remote diagnosis and treatment application has been widely used in China. In the application forums, users may seek the assistance of physicians who are qualified and accredited by the site. At the same time, the information provided by doctors is public, and supervision by the platform leads to a high level of quality for the questions and answers listed in these forums. However, consultations on DingXiangYuan are costly and restrict the number of conversations patients can have with their physicians. In addition, websites offering remote consultations, such as DingXiangYuan, still require physicians to respond on the web, which does not reduce the burden on clinicians.

Nevertheless, a comparative analysis of the quality of responses obtained from paid remote health consultations and ChatGPT-4 has yet to occur. This analysis was based on 82 orthopedic surgery–related consultations sourced from the Doctor DingXiang section of the DingXiangYuan platform. Responses from physicians on the web served as the control group, while those from ChatGPT-4 made up the experimental group. To determine the efficacy of ChatGPT-4 as a reliable remote health consultation resource, we conducted a comparative analysis of its logical response structure, diagnostic accuracy, the viability of its treatment recommendations, and the ability to effectively disseminate medical knowledge pertaining to various conditions. The goal is to provide a workable foundation for the development of ChatGPT-4 in the medical domain.

## Methods

### Data Set of Orthopedic-Related Remote Consultation

**Figure 1:**
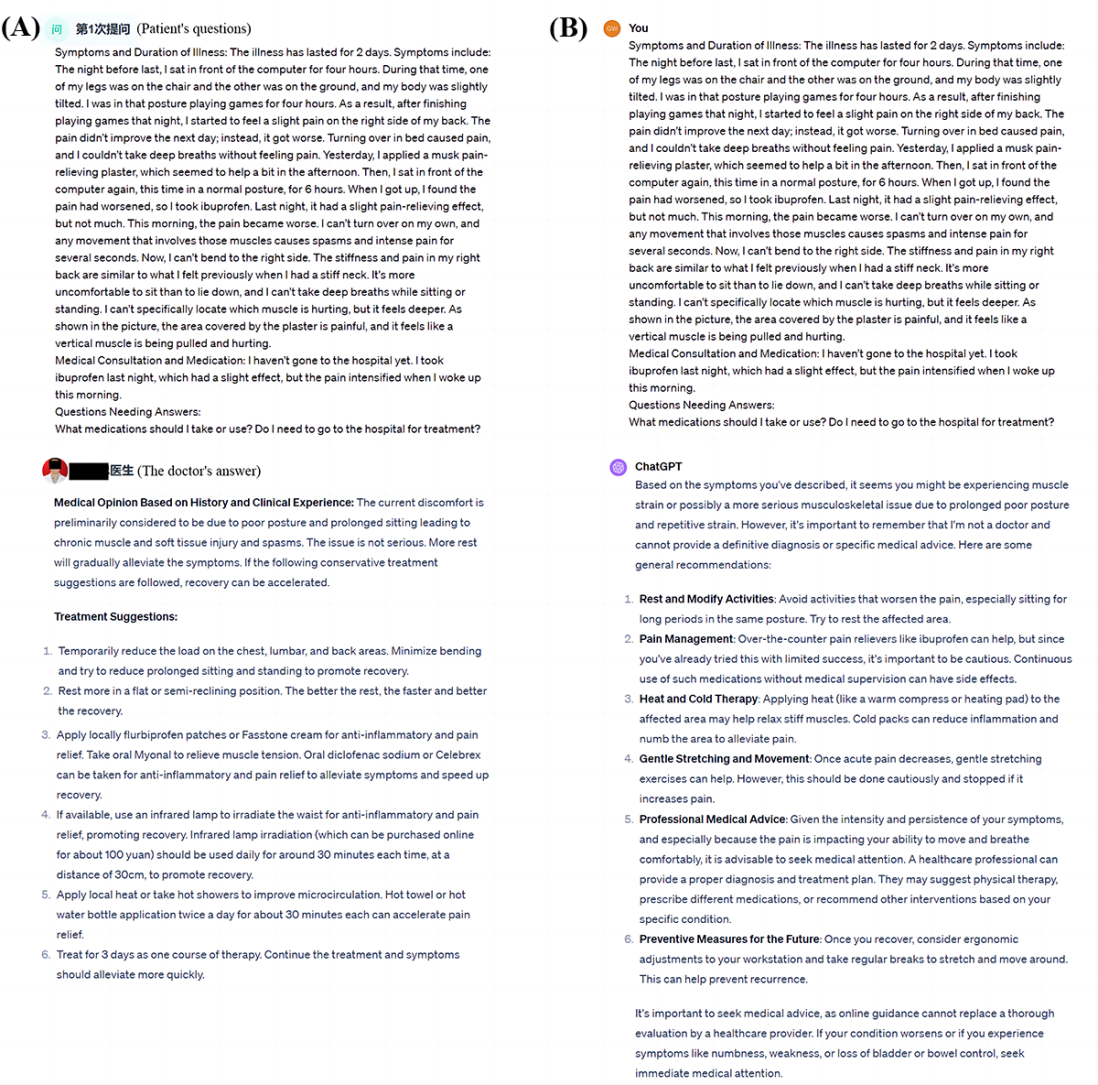
(A) Patient health consultation and certified physician's answer on the Doctor DingXiang website (translation from Chinese to English completed by ChatGPT-4). (B) The responses to the health queries were entered as Chinese text into ChatGPT-4. A high-definition version is available in Multimedia Appendix 3

**Figure 2:**
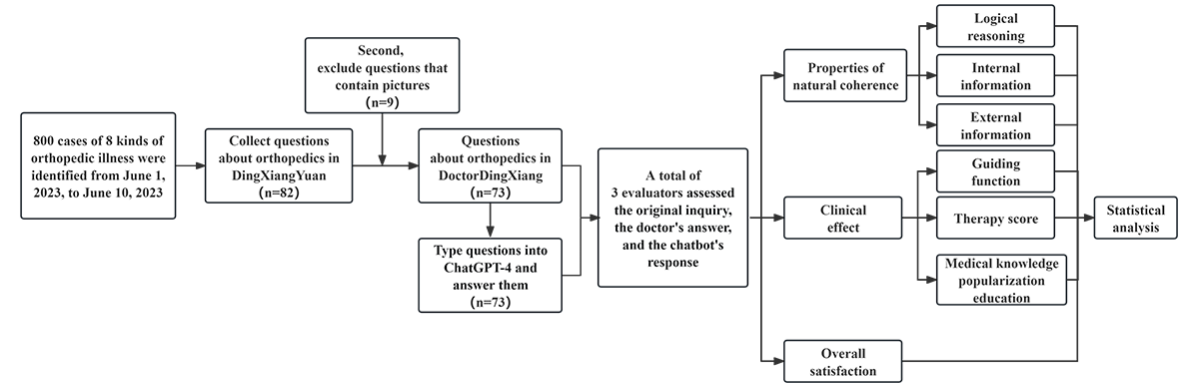
Flow diagram showing the study process.

The “Doctor DingXiang” website is a remote network that houses a collection of orthopedic-related medical dialogues and is one of China’s largest remote-paid consultation platforms (Figure 1A and Figure 2). To protect patients, the website blocks access to all content that may compromise their privacy, including the patient’s username, images provided in the question, imaging data, and biochemical examination results, from all other website visitors, allowing only the questioner and the target doctor to access it. In addition, there are categories of diseases on the site, and only about 100 consultation results are displayed for each type of disease. Each doctor’s response can be either spoken or written; however, since the spoken answers are not as accurate as the written answers and contain many spoken words, only the written answers were adopted (Multimedia Appendix 1). From May 20, 2023, to May 30, 2023, a total of 8 types of illness (with a total of 800 cases) were identified, namely gout, osteoarthritis, plantar fasciitis, fracture, osteoporosis, lumbar disc herniation, tendon sheath cyst, and osteoporosis. Of these, 82 patients originally met the screening criteria according to the above requirements. The 82 issues (Figure 1) we collected from this website are compliant with the HIPAA (Health Insurance Portability and Accountability Act) of 1996, given the information provided above [16]. “Doctor answers” refers to the website’s collection of responses from board-certified physicians (Figure 2A). Multimedia Appendix 2 contains all queries obtained from the Doctor DingXiang website, as well as the doctors’ responses.

### ChatGPT’s Answers

ChatGPT exhibits robust learning capabilities within the same dialogue window, enhancing responses to subsequent questions based on previous answers. However, this ability also introduces the potential for systematic error. To elaborate, this interconnectedness of responses does not allow for the maintenance of independence in ChatGPT-4’s answers to each question. Therefore, when 73 patients’ questions from the included consultations were entered into ChatGPT as questions (Figure 1B), a “new chat” was created for each question-and-answer set to minimize systematic errors. This process took place from June 1, 2023, to June 10, 2023. The use of a “new chat” for each inquiry ensured the independence of each response by preventing the AI from using context from previous interactions, thereby eliminating any learning or bias that may have been carried over from earlier questions. In addition, no plug-ins were used with ChatGPT-4, and the “chat history and training” option was deactivated to preserve the objectivity of each response. All ChatGPT-4 answers can be found in Multimedia Appendix 4.

### Response Qualification

The “data set of orthopedic-related remote consultation” was compiled by a professional orthopedic doctor on the Doctor DingXiang website, and 3 professional orthopedic physicians evaluated the ChatGPT and doctor response quality scores. To reduce systematic error resulting from human factors, the orthopedic surgeon who assessed the answers did not know how the answers were grouped. Specific scoring criteria were separated into “properties of natural coherence,” “clinical effect,” and “overall satisfaction” (Multimedia Appendix 5). The 3 orthopedic physicians convened initially to calibrate their scoring criteria using 2 examples provided by the author (Multimedia Appendix 6). After individual scoring, the Fleiss κ method was used to test the interrater consistency among the 3 physicians’ scores. The final statistical data were derived from the mean value of the scores given by the 3 physicians.

### Dependability of Comparative Analysis of Responses

When discussing the dependability of comparative analysis of responses, it is essential to consider 3 critical aspects: logical reasoning, internal information, and external information. These components collectively form the foundation for assessing the dependability of answers.

1. Logical reasoning: The answer uses logic and stepwise thinking to produce a response with the given information in the question stem.
2. Internal information: The answer uses information present within the question stem to procure a response.
3. External information: The answer uses external information to produce a response.

### Usability of Comparative Analysis of Responses

When assessing the usability of comparative analysis of responses in the medical field, it is crucial to focus on how effectively these analyses can guide diagnosis and treatment, provide therapeutic insights, and educate patients on their conditions.

1. Guiding function: To evaluate the accuracy of the provided diagnosis and differential diagnosis as well as the accuracy of the clinical treatment direction judgement and guidance.
2. Therapeutic effect: To determine whether the treatment suggestions provided in response to the consultation are accurate and if they can alleviate or treat the diseases proposed by the patients.
3. Medical knowledge popularization education: To evaluate whether the response introduces the cause and course of the disease and whether it can enhance patients’ understanding of the illness.

### Overall Satisfaction

On a scale of 1-10 points, the rater assigned a general rating to the replies. A score of 1-3 points indicated that the responses are biased and that they do not include contents that could call for differential diagnosis and certain auxiliary exams that need to be improved. A score of 4-6 points suggests that there is a possible danger of misdiagnosis or a delay in treatment. Scores of 7-9 points indicate consultation services that can practically replace licensed medical professionals. Finally, a score of 10 points indicates a full replacement for a licensed medical professional’s consultation service.

### Statistics

For statistical analysis, SPSS (version 26.0; IBM Corporation) was used. Chi-square analysis was used to analyze scoring differences between different groups. The Kolmogorov-Smirnov technique was used to determine whether the data exhibited a normal distribution; ultimately, it indicated that none of the data in this investigation were normally distributed. Consequently, the Mann-Whitney *U* test of independent samples was used to assess the disparity in scoring performance between the experimental and control groups [17]. When *P*<.05, the difference was considered statistically significant. Scott π statistic is a statistical measure of interrater reliability. Fleiss κ is a generalization of this statistic. SPSS was used to examine the consistency of the 3 raters for each item. Finally, GraphPad Prism 8 (GraphPad Software) was used to construct bar charts to display the comparison of dependability and usability between 2 types of responses, as well as the overall satisfaction outcomes.

## Results

### Orthopedic Case Selection and Comparative Assessment

We selected 8 orthopedic diseases from the Doctor DingXiang website and consulted 800 cases in total, namely fracture, osteoarthritis, cervical spondylosis, lumbar disc herniation, tendon sheath cyst, plantar fasciitis, osteoporosis, and gout. In the initial screening, we excluded 717 cases in which patients provided information that visitors could not view or where doctors used voice responses. The second screening process excluded patients who provided information that the visitor could not view in the follow-up questions (a total of 9 cases). Finally, 73 eligible cases were included. Without being aware of the replies’ origin, 3 orthopedic physicians in practice assessed the responses. The authors concluded by summarizing the statistical findings and designating the response assessment of Doctor DingXiang as the control group and the response evaluation of ChatGPT-4 as the experimental group.

### Evaluation Results for Dependability and Usability

**Figure 3:**
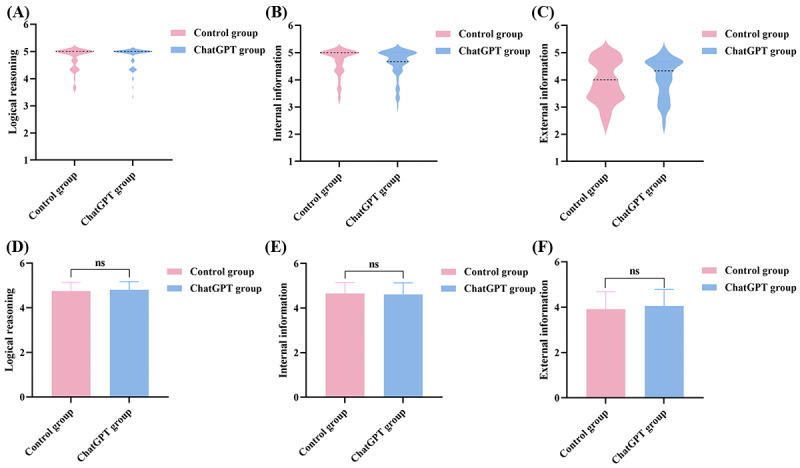
(A) The distribution of logical reasoning scores in the 2 groups. (B) The distribution of internal information scores in the 2 groups. (C) The distribution of external information scores in the 2 groups. (D) Logical reasoning scores of the 2 groups. (E) Internal information scores of the 2 groups. (F) External information scores of the 2 groups.

**Figure 4:**
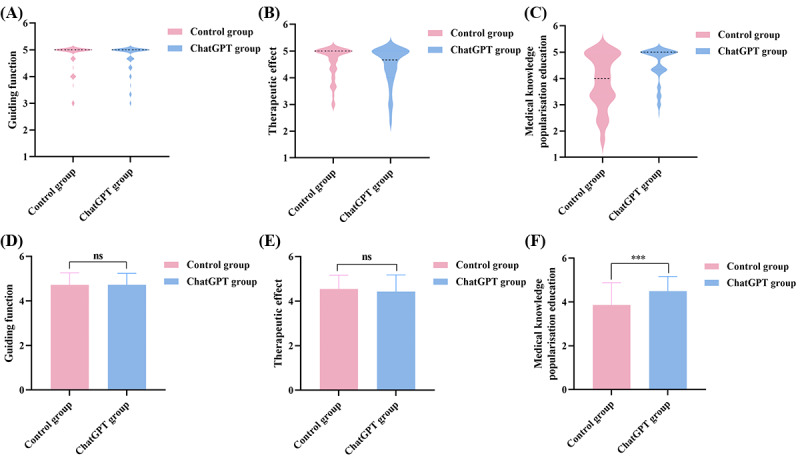
(A) The distribution of guiding function scores in the 2 groups. (B) The distribution of therapeutic effect scores in the 2 groups. (C) The distribution of medical knowledge popularization education scores in the 2 groups. (D) Guiding function scores of the 2 groups. (E) Therapeutic effect scores of the 2 groups. (F) Medical knowledge popularization education scores of the 2 groups (*P*<.001).

After statistical scoring, we discovered that ChatGPT’s “internal information” score (mean 4.61, SD 0.52 points vs mean 4.66, SD 0.49 points; *P*=.43) and “therapeutic effect” score (mean 4.43, SD 0.75 points vs mean 4.55, SD 0.62 points, *P*=.32) were lower than those of the control group, but the differences were not statistically significant (*P*>.05; Figures 3E and 4E). ChatGPT showed better performance in the “logical reasoning” score (mean 4.81, SD 0.36 points vs mean 4.75, SD 0.39 points; *P*=.38), “external information” score (mean 4.06, SD 0.72 points vs mean 3.92, SD 0.77 points; *P*=.25), and “guiding function” score (mean 4.73, SD 0.51 points vs mean 4.72, SD 0.54 points; *P*=.96), although the changes were not statistically significant (Figures 3D, 3F, and 4D). However, we were glad to see that, in terms of remote diagnosis and treatment, ChatGPT’s “medical knowledge popularization education” scores were better than those of the control group (mean 4.49, SD 0.67 points vs mean 3.87, SD 1.01 points; *P*<.001), and the difference was statistically significant (Figure 4F). Figure 3A depicts the score distribution of ChatGPT and the control group in terms of “logical reasoning.”

The figures show the distributions of “logical reasoning” (Figure 3A), “internal information” (Figure 3B), “external information” (Figure 3C), “guiding function” (Figure 4A), “therapeutic effect” (Figure 4B), and “medical knowledge popularization education” (Figure 4C). Other than that for “medical knowledge popularization education,” the score distribution for the remaining elements was roughly comparable.

**Figure 5:**
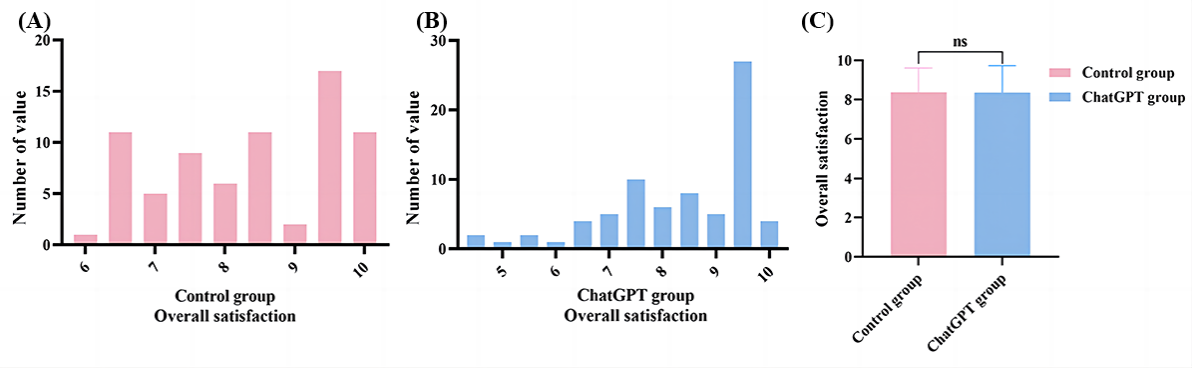
(A) The distribution of overall satisfaction scores in the control group. (B) The distribution of overall satisfaction scores in the ChatGPT group. (C) Overall satisfaction scores of the 2 groups.

In terms of “overall satisfaction,” we see that ChatGPT had slightly higher overall satisfaction scores of <5 points compared with the control group (Figures 5A and 5B), but the difference was not statistically significant (mean 8.35, SD 1.38 points vs mean 8.37, SD 1.24 points; *P*=.92; Figure 5C).

### Consistency Testing Among the 3 Orthopedic Physicians’ Evaluations

Using Fleiss κ, the consistency of the ratings among 3 physicians was determined (Multimedia Appendix 5). The Fleiss κ evaluations for “logical reasoning,” “internal information,” “external information,” “therapeutic effect,” “medical knowledge popularization education,” and “overall satisfaction” were rated as showing “fair agreement” for the control group, while “guiding function” was rated as showing “substantial agreement” (Multimedia Appendix 5; *P*<.001). According to Fleiss κ values, “internal information,” “external information,” and “overall satisfaction” were rated as displaying “fair agreement” in the ChatGPT responses, whereas “logical reasoning,” “guiding function,” “therapeutic effect,” and “medical knowledge popularization education” were rated as showing “moderate agreement” (Multimedia Appendix 5; *P*<.001).

## Discussion

### Main Findings of This Study

This cross-sectional research gathered 73 frequently asked clinical questions from patients and excellent responses given by licensed, qualified physicians on a reputable remote medical service website. After using ChatGPT to get the answers to these queries and seeking the evaluation of professional doctors, we discovered that, when compared with the responses of qualified clinicians, ChatGPT’s answers also showed strong logic and the capacity to extract and analyze key information, and they could help physicians respond to patients’ questions in a manner that reflects professionalism. Overall, ChatGPT’s responses received generally positive feedback from doctors. Professional responses from ChatGPT were able to assess and address queries in light of a large database, and the service even suggested literature on diseases for interested customers. With the help of ChatGPT, this method may unleash latent productivity, allowing health care personnel to use the time saved on more difficult duties. However, ChatGPT still has a lot of drawbacks. Although ChatGPT can analyze photographs, the procedure to do so is very complicated: medical images must be submitted to a public site to establish links for analysis, and the success rate of analysis is not very high. In addition, ChatGPT is not yet able to accurately diagnose a patient’s illness; this task must be left to expert physicians, whose assessment and oversight are crucial to the process [18]. Therefore, we believe that ChatGPT may successfully help doctors with remote diagnosis and treatment services, significantly increase clinicians’ job efficiency, and save more time, but it still cannot take over from the doctor entirely.

### Comparison With Previous Research

As the internet has grown, many hospitals have established remote medical services. Doctor-patient contact is no longer hampered by distance thanks to the internet, which makes it easier for both parties to interact. Remote medical services have expanded quickly over the last 3 years as a result of the COVID-19 pandemic, and, to some degree, they have even altered the conventional medical model. Remote diagnosis and therapy, nevertheless, are not yet flawless. Patients must pay additional costs for remote diagnostic and therapy services, and their communications may be ignored or they may receive pointless answers [2]. More crucially, in certain fields, including orthopedics, textual communication alone may be unable to provide clinicians with a whole picture of the patient’s condition. There is still no replacement for a physical examination, imaging examination, or biochemical test. In addition, physicians must expend a great deal of additional time and effort to decide how to respond to patients, which adds significantly to their burden and may not have the intended outcome [19].

Previous studies have indicated that ChatGPT-3.5 demonstrated strong performance in addressing public health inquiries on Reddit’s r/AskDocs, showcasing its considerable promise for offering remote medical consultation services [20]. This is noteworthy given the hesitancy of some patients to discuss their health issues publicly, coupled with the challenge of ensuring the reliability of unpaid responses on such platforms [20]. Contrasting with this, this study compares ChatGPT-4 with paid professional responses on the Doctor DingXiang forums, revealing that ChatGPT-4’s overall performance is comparable to that of paid medical professionals, with the added benefit of more effective dissemination of medical knowledge. In addition, ChatGPT’s low barrier to entry means this real-time, AI-driven, question-and-answer software better addresses the immediate health consultation needs of users, making it more significant for widespread application.

Interactive AI software that offers immediate feedback has an advantage over traditional AI analytical output software in that it allows users to inquire not only about the answers to “what” but also about the underlying “why” [21]. In the context of clinical scenarios, users have the ability to request critical information and foundations for diagnosis and treatment through ChatGPT. This functionality aids in the clarification of the operational logic behind their decisions, fostering greater transparency in the use of ChatGPT software and the comprehension of users. In relation to personal privacy, users have the ability to configure ChatGPT’s personal settings to “chat history and training” and enable a personalized input mode to proactively minimize the exposure of sensitive data.

### Significance for Hierarchical Diagnosis and Treatment as Well as Triage

A major worldwide problem is the scarcity and unequal distribution of medical resources. The issue is made worse in certain nations with high population densities, such as China, by the abundance of people who require medical treatment [22]. In addition, China is unable to guarantee the effectiveness of medical resource allocation, as other high-income countries can, due to ineffective rules and legislation and a lack of rigorously educated general practitioners [23]. To address this issue, the hierarchical medical system was created, and it has steadily replaced other medical systems to provide basic health care in the majority of high-income countries [24]. According to the severity and urgency of their sickness, patients must be sent to medical facilities of the appropriate level, such as primary medical institutions or specialized medical institutions [25]. This is a perfect medical paradigm, but patients’ treatment decisions are significantly influenced by their self-rated health state, chronic illnesses, socioeconomic situation, and educational level, particularly since the majority of patients lack an objective grasp of their ailment and pertinent medical expertise [26,27]. The hierarchical medical system has not had the desired impact in China as a consequence of its deployment. Some medical facilities are suffering from severe work pressure overload due to a lack of medical resources and patients’ unrealistic treatment preferences [23]. To enhance patients’ medical behavior and help them choose the best medical facilities, high-quality guiding services are thus necessary to assist patients in understanding their disease-related information before treatment [28]. This may somewhat mitigate the issues brought on by a lack of medical resources and assist patients in receiving more focused and appropriate medical care.

Patient navigation services, a patient-centered intervention, are becoming more and more popular. These services use trained personnel to identify patient-level barriers to care, such as cultural, logistical, and educational ones, and then remove them to encourage full and prompt access to care [29,30]. A growing body of research demonstrates the beneficial effects that patient guide services have on illness prevention, the spread of health information, medical decision-making, and communication promotion. Patient navigation can help remove barriers brought on by language, cultural differences, a lack of relevant medical knowledge, and other factors, especially for some patients with a lack of medical knowledge or a relatively low level of education, in the face of a more complex but hierarchical medical center or sociomedical system. This will lead to a more effective patient path and fewer delays in diagnosis and treatment [31]. More crucially, research has demonstrated that patient guiding services have benefited people with chronic illnesses such as diabetes and cardiovascular disease and have somewhat decreased the likelihood of rehospitalization [32]. Patient guidance services may not only aid in the patient’s healing process but also assist them with developing a more thorough and expert understanding of the causes, symptoms, and other facets of associated diseases, enabling them to treat, care for, and monitor their disease more skillfully and effectively [33]. However, previous research discovered that some issues remain with the present patient guidance service, such as navigators’ potential lack of expertise. In addition, some patient navigators, although trained on how to perform their job, lack a history of medical education, making it difficult for them to respond to the patient’s consultation [11]. Even if it may be a little harsh to demand that patient navigators be all-knowing, finding practical and trustworthy approaches to boost the effectiveness and caliber of patient navigation services is still necessary.

### The Challenges of Promoting ChatGPT in the Medical Field

While using AI is the general trend in science and technology development, individuals must also understand that the tool can only work optimally in the ideal regulatory environment, which often has some lag. To ensure the rational use of ChatGPT in the medical field, hospitals need to organize training on the use of ChatGPT and uniformly manage the accounts used by doctors during working hours. Doctors must also take responsibility for assessing the quality of ChatGPT’s responses and ensuring that the patient’s right to be informed of the use of ChatGPT is met. Specifically, physicians are required to assign a unique account when using ChatGPT in clinical practice, and they must also have the corresponding patient present. The physician has the authority not only to assess the quality of ChatGPT’s responses before presenting them to the patient but also to provide the patient with the final interpretation of said responses. Conversely, individuals who use ChatGPT but do not identify as medical professionals should refrain from relying exclusively on it for health-related information.

ChatGPT can assist clinicians in better organizing clinical data, analyzing imaging results, and providing personalized support for clinical decision-making regarding cancer patients, according to recent studies [34-36]. As previously stated, physicians, in their capacity as users of ChatGPT, are additionally obligated to oversee its use. In this regard, ChatGPT functions as a supplementary tool. Should ChatGPT outputs be incorporated into the physician-patient communication and clinical decision-making process, the physician must disclose the information source to the patients to guarantee that they are well-informed. Simultaneously, the hospital must oversee the ChatGPT accounts used by physicians and coordinate training courses on ChatGPT usage to guarantee that physicians who use ChatGPT in their clinical practice possess a certain level of proficiency in its operation. By implementing these management tasks, certain potential hazards and medical disputes can be circumvented, and the application of AI software in the medical field can be promoted more effectively.

Although this study establishes a sound theoretical foundation for the clinical implementation of ChatGPT, there are numerous areas still requiring further refinement. As one example, there is a need for further refinement of cross-sectional experiments in the future to compare the following: the quality of answers provided by AI software in various clinical disciplines, variations in the quality of answers generated by different AI software programs (ChatGPT, Google Board, Claude, and so forth), and disparities between different language inputs used by AI software. Alternatively, a randomized controlled trial could assess the efficacy of ChatGPT as a supplementary tool for clinicians to use while interacting with patients. Further development is required to ensure the full functionality, safety, and dependability of ChatGPT as a medical AI.

### Limitations

Initially, we intended to investigate the viability of using the ChatGPT app for medical guidance. This study solely included orthopedic cases as the research object and did not gather multidisciplinary clinical cases to rule out variations in the difficulty levels of working in other clinical specialties, which may produce different findings. In the future, it will be possible to aggregate challenges from many disciplines and examine how AI performance differs between fields in solving difficulties. Furthermore, neither machine translation nor manual translation can preserve the flaws and precision of the original sentence content. Users are unable to ascertain the processing logic of AI when using ChatGPT as a research tool across different language types. Consequently, they are limited to inputting ChatGPT data in accordance with the language type used in the control content and assessing the output quality of ChatGPT content in the same language. Medical personnel are required to use ChatGPT under a special number with a real-name system for supervision purposes. As an auxiliary tool, ChatGPT users are not only tasked with assessing the quality of responses but also possess the authority to make the ultimate interpretation of the content. Ultimately, further randomized controlled trials are required in the future to validate the use of AI in medicine while controlling for confounding variables, as this study was cross-sectional in nature.

### Conclusion

This study demonstrates that ChatGPT-4 responses match the expertise found among health care practitioners on DingXiangYuan, a leading remote medical consultation platform in China, across various metrics such as logical reasoning and diagnostic accuracy. Notably, it excels at providing scientific education. ChatGPT-4 is thus recommended as an alternative to traditional remote health consultations. It can assist physicians in educating patients, thereby enhancing medical knowledge dissemination. For patients, it offers accessible, reliable health advice, improving information accessibility and decision-making support. These findings suggest a transformative potential for ChatGPT-4 in health care, notably in enhancing access to medical advice and patient education. It implies the need for advancing medical AI with a focus on ethical and transparent applications, highlighting its role in improving health care delivery and patient empowerment.

## Appendix

Multimedia Appendix 1 Select inclusion criteria and exclusion criteria for website consultation dialogue information.

Multimedia Appendix 2 The 82 patient questions and online responses from doctors on the Doctor DingXiang website (translated from Chinese to English using ChatGPT-3.5).

Multimedia Appendix 3 High-resolution version Figure 1.

Multimedia Appendix 4 ChatGPT-4 responses after asking questions from 82 patients in Chinese (translated from Chinese to English using ChatGPT-3.5).

Multimedia Appendix 5 Consistent evaluation of Fleiss κ among the 3 raters.

Multimedia Appendix 6 Orthopaedic physicians’ initial criteria calibration.

## Abbreviations

AI: artificial intelligence
HIPAA: Health Insurance Portability and Accountability Act
