# Supplementary material for: Quality and Dependability of ChatGPT and DingXiangYuan Forums for Remote Orthopedic Consultations: Comparative Analysis
Source: J Med Internet Res. 2024 Mar 14;26:e50882. doi: 10.2196/50882 (PMC10979330; doi:10.2196/50882)
Supplement: Multimedia Appendix 1 [file jmir_v26i1e50882_app1.docx]

| Inclusion criteria | Exclusion criteria |
| --- | --- |
| Make an enquiry on the DOCTOR DINGXIANG website | The patient has uploaded picture information |
| Orthopedic surgery-related problems | The doctor's answer is in the form of speech rather than text. |
| The doctor is certified |  |
| Questions and answers are in Chinese |  |

**Table S1**. Select inclusion criteria and exclusion criteria for website consultation dialogue information.
