# Supplementary material for: Quality and Dependability of ChatGPT and DingXiangYuan Forums for Remote Orthopedic Consultations: Comparative Analysis
Source: J Med Internet Res. 2024 Mar 14;26:e50882. doi: 10.2196/50882 (PMC10979330; doi:10.2196/50882)
Supplement: Multimedia Appendix 2 [file jmir_v26i1e50882_app2.docx]

1.

Symptoms and Duration of Illness: Joint pain when exposed to cold, especially during the summer when the room is cold. It mainly affects the shoulder, elbow, wrist, knee, ankle joints, and hand joints that cannot touch cold water. The pain caused by cold water is quite severe and can be felt deeply. I believe I developed this condition around the summer of 1996 when I was living in a damp house. The joints were painful, but wearing knee pads for warmth helped. Even now, staying warm helps alleviate the pain. I am not a physically demanding worker and do not do much housework. Currently, there is no deformation in any of the joints. Sometimes, I experience morning stiffness in the finger joints, but it improves with movement. I saw doctors about this condition in my early years, but it was never officially diagnosed. The pain flares up when exposed to cold, and it has recently started bothering me again. It's a cold, tingling pain that doesn't affect my sleep, and keeping warm helps.

Medical History and Medication: I have not taken any medication for this condition. I recently had a calcium test, and I do not have a calcium deficiency; I am only deficient in vitamin D3.

Questions to Answer: What is this illness, and how should it be treated?

**The doctor's answer on the website**: Hello, your inquiry has been received. Thank you for your trust! For our joints, they are generally located close to the surface of the skin because joints need to move easily and are not typically deep within the body. Therefore, they tend to be sensitive to changes in the environment. In your case, if there are other underlying conditions, it could significantly amplify your discomfort. The most common condition is arthritis, which comes in many forms, including osteoarthritis and rheumatoid arthritis. Based on your description, osteoarthritis is a more likely possibility, but further examination is needed. I have a few recommendations: First, I suggest you go to a local hospital and get some X-rays taken. Focus on the joint that has the most pronounced and severe impact on your quality of life, and evaluate the specific degree of degeneration in your bone structure. Second, we need to assess whether you have rheumatism or rheumatoid arthritis. I recommend getting a blood test, including a complete blood count, ESR (erythrocyte sedimentation rate), C-reactive protein, antinuclear antibody, and rheumatoid factor. Third, is your menstrual cycle currently normal? Have there been any changes in its frequency or duration? As the body's hormone levels gradually decline, it can make you more sensitive to environmental changes, which is a natural process in our bodies and could be a possible reason. You can get these tests done to further evaluate your condition.

2.

Symptoms and Duration of Illness: A 31-year-old male experiences pain in the fingertips of two fingers, especially when typing. The thumbs also have slight pain and morning stiffness, making it difficult to grip in the morning. The symptoms disappear after about 10 minutes. Due to his profession, he types approximately 10,000 words daily. The soreness is mainly concentrated in the fingertips of both hands. There is mild pain in the finger joints. He has a habit of using a mobile phone extensively, typing around 10,000 words daily. The discomfort includes a sense of swelling mainly in the fingertips of both hands. There is slight pain below the fingertips, and typing exacerbates the pain, especially in the thumbs. The swelling doesn't extend from the wrist to the fingers. He previously had a lumbar muscle strain diagnosis but no other illnesses. Pain relief is achieved with warm water soaks, and avoiding pressing on the phone also reduces pain. There is no aggravation or sharp pain when tapping the center of the wrist. No drug allergies have been reported.

Medical History and Medication: In the past three days, he has taken ibuprofen and applied diclofenac diethylamine gel, but he has not visited a hospital.

Questions to Answer: What is the condition, and what medication should be used?

**The doctor's answer on the website**: Hello, considering that you type a lot and experience fingertip pain, especially when typing, the most likely possibility is "computer hand," which is a form of soft tissue strain caused by prolonged typing.

Recommendations:

Reduce your activity level, decrease the amount of typing, and avoid movements that cause pain. Consider switching to typing on a computer keyboard.

Ensure that your wrists are as flat as possible when operating, avoiding bending or hanging them.

Maintain an elbow working angle greater than 90 degrees to avoid pressure on the median nerve in the elbow.

Keep the angle between your upper arm and torso at 45 degrees. This position is appropriate for the distance between your body and the mouse, and try to stay relaxed to avoid leaning forward when using the mouse.

Keep your wrist straight when typing and maintain an upright posture. Use a high-quality backrest, and place both feet flat on the floor or on a footrest.

Position the computer monitor at a height that doesn't require excessive up and down movement of your head. Ensure that your eyes are in line with the screen when sitting upright, and adjust the screen's brightness to a moderate level.

Select a suitable desk, chair, and keyboard and mouse set for yourself.

During work, regularly stretch and relax your hands. You can slowly bend your wrists for 10 seconds every hour, or you can continuously make fist motions for 10 seconds every hour.

Apply warm compresses to the areas experiencing swelling and pain twice a day, for 30 minutes each time. You can use a hot water bag for this purpose. The temperature of the warm compress should not be too high to avoid burning the skin. Generally, a temperature of around 60°C is suitable, slightly higher than body temperature. Any temperature between 50-80 degrees Celsius is acceptable. If it cools down, replace it with a warm towel. Be sure not to burn your skin. If the hot water bag's temperature is relatively high, use a dry towel as a barrier to avoid direct contact with the skin.

You can take Celebrex orally, one 200mg tablet per day. It has anti-inflammatory properties and can be used for approximately two weeks.

Please consult a healthcare professional for a proper diagnosis and personalized treatment plan.

3.

I apologize for the misunderstanding. Here's the translation of the information you provided from Chinese to English:

Symptoms and Duration of Illness: Duration of illness is 2 days. Symptoms: Two days ago, after sitting for four hours in front of the computer, with one leg on the chair and one leg on the floor, the body was slightly tilted. In this posture, the person played video games for four hours. After finishing the game that evening, they felt slight pain in the right upper back. The next day, the pain not only didn't improve but worsened. Turning over during sleep caused pain, and even deep breaths were painful. Yesterday, during the daytime, they applied a musk pain relief patch, which provided some relief in the afternoon. However, after sitting in a normal position in front of the computer for 6 hours, they noticed that the pain had worsened. So, they took ibuprofen and had some pain relief last night, although it was limited. This morning, the pain has increased further, and they can't turn over voluntarily. Any position that uses the affected muscle results in severe spasms lasting several seconds. The body can't bend to the right side, and the pain in the right upper back is similar to the stiffness and pain they experienced during a previous neck sprain. Sitting is more uncomfortable than lying down, and sitting or standing makes deep breathing difficult. The specific area of the painful muscle is difficult to pinpoint, and it feels deeper. The range of pain corresponds to the length of the patch, and it feels like a vertical muscle is pulling.

Medical History and Medication: They have not visited the hospital yet. They took ibuprofen last night, which provided slight relief, but the pain worsened upon waking up this morning.

Questions to Answer: What medications should I take, and do I need to visit a hospital?

**The doctor's answer on the website**: Hello, based on the medical history and clinical experience, the current discomfort is likely due to poor posture and prolonged sitting, leading to secondary chronic muscle soft tissue injury and spasms. There shouldn't be a major issue, and with some rest, your symptoms should gradually improve. If you follow the conservative treatment advice below, it can speed up your recovery.

Treatment Recommendations:

Temporarily reduce the load on your chest, waist, and back, minimize bending, and try to limit prolonged sitting or standing to facilitate recovery.

Rest in a flat or semi-reclined position as much as possible. The better you rest, the faster and better your recovery will be.

You can use a local fluorobiphenyl patch or Fasutong ointment to reduce inflammation and pain. Taking Miouna orally can help relieve muscle tension. You can also take sodium diclofenac or Celebrex for anti-inflammatory and pain relief purposes, which can alleviate symptoms and expedite recovery.

If you have access to an infrared lamp, you can use it to irradiate the lumbar region for anti-inflammatory and pain relief, promoting recovery. Infrared lamp irradiation (available for purchase online, costs about 100 yuan) can be done approximately twice a day, each session lasting about 30 minutes, with a distance of 30 cm to the skin.

Local pain relief with hot compress or hot showers can also help improve local microcirculation. Hot compress with a hot towel or hot water bag for about 2 times a day, each time lasting about 30 minutes, can accelerate pain relief.

Stick to the treatment, and generally, a 3-day course should lead to relatively quick relief of symptoms.

If your symptoms do not improve or continue to worsen, it is recommended to seek medical attention promptly for further evaluation and diagnosis by a healthcare professional, who can provide more advanced treatment recommendations if necessary.

4.

Symptoms and Duration of Illness: A 22-year-old female with no history of gout or other illnesses experienced an injury on May 6th when she fell while sitting, with her right buttock and the inside of her left knee simultaneously hitting the ground (on a flat surface without water). Initially, her entire knee swelled severely, with a bruise area larger than the palm of her hand. When she didn't move, there was no specific pain sensation, but bending her leg was difficult, and there was a sense of heaviness when standing up. However, the condition has gradually improved. Later, the bruising partially disappeared, but there was still swelling around the shinbone area, which was tender when pressed. When her leg is flat, there is only slight swelling, but when bending her leg, there is a significant protrusion. Currently, she can bend her leg beyond 90 degrees without pain. Between 90 to 110 degrees, there is tightness and some pain, but it is tolerable. Further bending causes significant pain, and she hasn't attempted it much. On May 26th, she went to school for an exam, walking very slowly, and the pain wasn't very severe at the time. She walked for approximately 500 meters, encountered some stairs, and sat for about 100 minutes, after which her knee felt very uncomfortable. After returning home, she felt a stabbing pain in her knee. She rested at home for a few days, and the pain gradually subsided.

Medical History and Medication: Due to inconvenience, she hasn't sought medical attention yet. In the initial two days, she applied ice at home. In the first week, she started using Bengay muscle pain cream and has been wearing a knee brace similar to a sports knee support since then. She has mostly stayed home and performed leg elevation exercises later.

Questions to Answer: I would like to ask Dr. Wang the following questions: Do you think there is a high probability of fracture or bone crack? Why is the knee protruding so much, is it fluid accumulation or some inflammation? How long does it typically take to return to normal? What should be done next? Thank you.

**The doctor's answer on the website**: Hello, I've reviewed the information you provided, including the description of your knee injury and the photos you shared.

Fractures usually come with significant pain that is difficult to alleviate and may persist for an extended period. Therefore, if your current pain is not very severe, the likelihood of a fracture may not be very high. However, based on the photos you shared, including the significant initial swelling and bruising, it suggests that there might have been some significant structural and tissue damage. This could include fractures, ligament injuries, or meniscus injuries, which can cause noticeable bleeding. Therefore, I cannot definitively rule out the possibility of a fracture.

Regarding the protrusion in your knee, it's likely due to the accumulation of blood within the joint. When an injury is severe enough, it can cause bleeding into the joint space, and the blood has nowhere to go, leading to swelling and discomfort. As the red blood cells and hemoglobin in the blood are slowly absorbed, it can transform into fluid accumulation.

The recovery time varies depending on the severity of the injury. For relatively minor soft tissue injuries, it may take about two weeks. If the injury is more severe, it may require 6 to 8 weeks. In cases of significant muscle and bone injuries, as the saying goes, "injured muscle and bone take 100 days." Some injuries may even result in incomplete healing, such as severe meniscus injuries.

As a Western medical perspective, without undergoing imaging studies such as X-rays, it's challenging to determine the nature and severity of the injury definitively. Therefore, if possible, it's advisable to visit a hospital for imaging tests to get a clearer diagnosis.

From a management perspective, regardless of the nature of the injury, the most crucial aspect is rest. The human body has its own healing capabilities, and proper rest is essential for recovery. Reduce any burden on the injured limb and immobilize it to minimize movement, which is protective. However, prolonged immobilization should be avoided, typically not exceeding two months. Extended immobilization may lead to joint stiffness and adhesions.

During the rest and recovery period, you can consider using topical medications like Futheron ointment to alleviate local post-traumatic inflammation. Still, remember that rest and immobilization are the primary methods of protection. After an extended period of rest, your leg muscles may weaken due to lack of use. Targeted rehabilitation exercises will be necessary at that point. When the time comes, you should seek consultation at a rehabilitation department, where a rehabilitation physician can design a rehabilitation exercise plan based on your condition and guide you through the process.

5.

Symptoms and Duration of Illness: I had experienced lumbar discomfort in the past, which improved after using medicated patches and performing exercises. However, two days ago, I went for a long jump wearing thick-soled shoes and jumped about two meters. I ended up kneeling on the ground and couldn't get up. After managing to stand up, I experienced significant pain in my lumbar spine. After three days, it improved slightly, but I still feel a sensation of my lower back sinking when sitting on the bed. I can't bend or squat down, and I need to hold onto something to get up for various activities. I don't experience pain when walking.

Medical History and Medication: I haven't gone for an X-ray yet. In the meantime, I have taken Panlong Seven Pills and used medicated patches.

Questions: I would like to ask if it's possible that I have lumbar spondylolisthesis or a lumbar disc protrusion.

**The doctor's answer on the website**

Hello, thank you for your trust. Based on your description, it seems that you had pre-existing discomfort in your lower back, and recently, after performing a long jump, you experienced lower back pain and restricted mobility.

From your description, if it's solely lower back pain without any numbness, weakness, or pain radiating down your legs, the primary consideration would be inadequate warm-up before the long jump. This could result in injuries to the supraspinous ligaments, muscle strains in the lower back, or minor muscle sprains.

This appears to be related to muscle and ligament issues, and if there is no clear history of trauma, the likelihood of bone involvement is minimal. The possibility of lumbar spondylolisthesis (vertebral slippage) is also quite low. Even if lumbar spondylolisthesis were present, it would likely be related to pre-existing developmental factors, such as a congenital spondylolysis, and intense physical activity may have exacerbated the condition, leading to lower back pain.

However, since you don't mention any leg pain, numbness, or weakness, even if there is a minor injury, it is not a significant concern. If you want to confirm the diagnosis, you may consider getting a lumbar spine X-ray in the anteroposterior and lateral views.

If there is no leg pain or numbness, there's no immediate need for a lumbar spine CT scan or MRI. If visiting a hospital for examination is inconvenient at the moment, you can wear a lumbar support belt to protect your lower back. Try to avoid prolonged sitting, standing, or bending, and avoid maintaining the same posture for extended periods.

For pain relief, you can use topical anti-inflammatory and analgesic ointments like "扶他林" (Futalin) or take oral non-steroidal anti-inflammatory drugs (NSAIDs) such as sodium diclofenac or celecoxib. These medications can help reduce inflammation and alleviate pain.

Based on your current description, the issue doesn't seem to be of significant concern, so there's no need to worry too much.

6.

Symptoms and Duration of Illness: On May 18th, I accidentally bumped my left knee on the outer side. Since then, when I exert force to bend my left knee, it hurts. Bending the knee without applying force doesn't cause pain. However, when the knee is bent under stress, there is severe pain on the outer side of the left knee. A similar symptom occurred in 2012, diagnosed as tendonitis, which was successfully treated. It has been about a week since the current medication regimen, and while the pain has decreased, there is still noticeable discomfort when bending the left knee under stress. Pressing on the outer side of the left knee with my hand also causes pain.

Medical History and Medication: On May 21st, I visited the hospital, and the doctor diagnosed it as tendonitis again. The prescribed medications include Nimelusil dispersible tablets, Xianling Guobao capsules, and anti-inflammatory pain relief patches.

Questions to Address:

My prescribed medications are running out, and the patches are used up. The outer side of my knee still hurts. How should I proceed with medication?

How long is the expected recovery period?

Should I avoid exerting force on my left knee or engage in moderate exercise during the recovery period?

**The doctor's answer on the website**

Hello, based on the medical history and clinical experience, the current discomfort in the knee area is initially considered to be related to soft tissue damage, possibly involving the iliotibial band and the muscles on the outer side of the thigh, due to the previous trauma. It doesn't seem to be severe, but it would be beneficial to undergo a knee magnetic resonance imaging (MRI) scan to assist with diagnosis. Currently, it is advisable to rest, avoid strenuous exercises, and refrain from deep squats. Recovery may take around 2-3 weeks. In the meantime, you can follow the conservative treatment suggestions below to promote healing.

Treatment recommendations:

Temporarily reduce activities that cause discomfort in the affected area and avoid heavy lifting. Avoid vigorous exercise and try to minimize prolonged sitting or standing. Rest as much as possible, as good rest leads to faster and better recovery. Wearing a knee brace can aid in recovery.

You can apply local heat therapy to the painful area to improve blood circulation. Apply a warm towel or hot water bag to the affected knee for about 30 minutes, approximately twice a day, to expedite symptom relief.

Consider using local pain relief patches containing flurbiprofen or apply topical analgesic gels like Fastum. These can help alleviate pain and inflammation. You can also take oral anti-inflammatory medication like flurbiprofen or Celebrex to assist with pain and inflammation.

If you have access to an infrared lamp, you can use it to irradiate the painful area. This can help expedite symptom relief. Infrared lamp therapy (available online for approximately 100 yuan) should be applied for about 30 minutes, twice a day, at a distance of 30cm from the affected area. If you don't have access to an infrared lamp, continue with the other treatment recommendations.

Follow the treatment regimen for a full 2-week course, and adhere to the treatment plan. Symptoms should improve relatively quickly.

7.

Symptoms and Duration of the Condition: Since May 7, 2023, I have been experiencing discomfort in the muscles above the left hip joint and the left buttock, which can radiate to the back of the left thigh. Occasionally, when I exert force on the left side, I feel uncomfortable. Resting for a while alleviates the symptoms, and it doesn't significantly impact my daily life. Starting on the same day, I also began to experience limited rotation and pain in the right wrist and forearm. Resting briefly makes the symptoms disappear. I have occasionally experienced muscle soreness in the lower back in the past, but adjusting my posture resolved it completely.

Medical Consultation and Medication: I have not sought medical attention for these issues. I have applied topical pain relief cream (such as Voltaren) to the lower back, buttocks, and wrist, but the results have not been significant.

Questions Needing Answers: I have eight years of experience in marathon running, including a full marathon and a half marathon this April. In early May, I added abdominal muscle training to my routine, and that's when the symptoms in my lower back and wrist began. Could these symptoms be related to continuing exercise after the marathon or improper abdominal muscle training that may have caused injury to the lower back? I have stopped running for 20 days, and my weight has increased rapidly. Starting on May 28, I have been doing extremely slow 2.5-kilometer runs daily. Apart from a slightly elevated heart rate, I feel okay during these runs. Can I continue with these slow runs? What treatment recommendations do you have?

**The doctor's answer on the website**

Hello, based on your description, it appears that the discomfort in your lower back and hip region is likely due to muscle and soft tissue strain in the lower back. However, the overall severity seems relatively mild, and with some rest, there has been noticeable improvement. If you can engage in slow jogging without significant discomfort, you can continue.

For the lower back and hip area, it's a good idea to apply heat therapy. Since the skin in this area is thicker, topical creams like Voltaren may not be very effective. You can use a hot water bottle to apply heat, which can promote blood circulation and accelerate tissue repair.

Regarding the issue with your wrist, it may be related to the abdominal wheel exercise you've been doing. It's advisable to take a break from this exercise, as it seems your wrist joint is not tolerating the intensity of the activity well.

8.

Symptoms and Duration of Illness: It has been 97 days since my cesarean section. I noticed this protrusion a few days after the C-section. At that time, I thought it was a normal protrusion of the sternum. I rarely breastfeed in a lying position. I only discovered the asymmetry of the ribs today. I only feel uncomfortable when lying on my side to breastfeed, but I don't experience any discomfort during daily activities or when sleeping on my side. How should I treat this?

Medical History and Medication: No medication has been used.

Questions Requiring Answers: How should I treat this?

**The doctor's answer on the website：**

Hello, thank you for your trust. I just looked at your question, mainly about the asymmetry in the ribcage area with some mild discomfort. However, from the appearance, there are no apparent abnormalities. I have marked the protruding area in the picture.

The central protrusion is the xiphoid process, which is a normal bony prominence. The protrusions on both sides are called the costal arches, which are part of the edges of the ribs.

If there is no clear trauma, no significant tenderness, and no signs of bruising or swelling, the bones are generally fine. Discomfort in this area may be related to improper breastfeeding positions or prolonged maintenance of one position when taking care of your child. It could also be costochondritis, a non-infectious inflammation. Rest is essential, avoiding excessive fatigue, and applying heat to the painful area to improve local blood circulation can help alleviate symptoms.

If the pain is significant, you can use non-steroidal anti-inflammatory drugs (NSAIDs) like ibuprofen to reduce inflammation and pain.

If you are breastfeeding, try to minimize the use of oral medications. Rest, local heat application, and these measures should generally help relieve the symptoms. Overall, it's not a significant issue, and there's no need to worry. There is also no need for X-ray imaging at this time.

9.

Symptoms and Duration of Illness: I started experiencing lower back pain yesterday (May 28th). This morning, I also felt pain in my left hip and a slight ache in my left thigh. During lunch, I squatted for a while, and that's when I suddenly experienced severe pain in my left hip. I immediately found a chair to sit on, but the pain didn't subside. At this point, I couldn't stand up anymore. When I try to stand, my left hip and thigh hurt intensely. I managed to slowly move to the bed, where I can only lie flat. Even when lying flat, there's still some discomfort in my left hip, but it's bearable. I fell asleep in this position for a while. I have now woken up, and I still feel some pain in my left hip and lower back. I want to sit up and stand, but it's impossible. When I try to get up, my left hip and thigh experience severe pain.

Medical History and Medication: Currently, I haven't taken any medication or visited a hospital. Two years ago, I had an X-ray, and the doctor mentioned a slight herniated disc.

Questions Needing Answers: What medication can I take right now to alleviate the pain temporarily and enable me to stand up? After that, I plan to visit a hospital for treatment.

**The doctor's answer on the website**

Hello, thank you for your trust. I just read your situation, which primarily involves lower back pain along with pain in your left hip and lower limb. Based on your description, it is possible that you have a herniated disc in your lumbar spine that is compressing a nerve.

Considering your medical history, it's likely related to a previous herniated disc. The current symptoms, including pain in the lower back and down the left leg, suggest nerve compression due to the disc herniation. The pain might be exacerbated when you bend over or squat, as these movements increase the load on the lower back, leading to increased pressure on the herniated disc and nerve compression.

In your current situation, it's crucial to avoid prolonged sitting and bending over. If possible, rest in a supine position, and you can wear a lumbar support belt to protect your spine. Applying heat to the painful area of your lower back may provide relief. If the symptoms are significant, you can consider taking oral non-steroidal anti-inflammatory drugs (NSAIDs) for pain and inflammation relief, such as sodium diclofenac or celecoxib.

If you don't have access to these medications, ibuprofen may provide some pain relief, although it might be less effective. The primary goal is to reduce the load on your lower back. Once the pain subsides, it's essential to seek medical attention promptly. During your outpatient visit, the doctor may decide whether to perform a CT scan or MRI of the lumbar spine based on a physical examination.

Both CT and MRI scans can visualize the lumbar intervertebral discs, but MRI is generally more accurate. However, MRI appointments may have longer waiting times. If there's a substantial wait for an MRI, you can consider getting a CT scan first, as it can also provide clear imaging of the lumbar discs. If you experience radiating pain and numbness in your lower limbs, the doctor might also recommend taking methylcobalamin to support nerve health.

10.

Symptoms and Duration of Illness: Two weeks ago, I began experiencing pain in my right ankle, similar to the pain of a sprained ankle. The pain is particularly pronounced when standing and walking, and each time it occurs, it lasts for 2-3 days before subsiding. My right ankle has not been subjected to recent injury, but I started running suddenly in May without proper warm-up. The pain tends to occur on overcast days. It even hurts when there's no weight on it. The primary area of pain is on the left side of my ankle near the back of the heel, with no pain on the top of the foot. Currently, standing and walking are very painful, and the inner side of the ankle is also experiencing increased pain. Each episode of pain has specific triggering factors. There is no redness or increased skin temperature in the entire ankle area, but pressing on it causes pain.

Medical History and Medication: I have not sought medical attention or used any medication for this issue.

Questions to be Answered:

Why is my ankle experiencing pain?

How can I alleviate this pain?

**The doctor's answer on the website**

Hello, based on the description of your condition, the possibility of a foot tendon injury is quite likely.

The primary anatomical structures in the area of your pain are the posterior tibial tendon and the flexor tendons of the toes. These tendons slide over the calcaneus bone. There is a tendon sheath in this area where the tendons normally slide smoothly back and forth. However, sudden and intense physical activity can lead to swelling and inflammation of the tendons, resulting in the type of pain you're experiencing.

Here are my recommendations for your specific situation:

Make sure to immobilize your ankle and foot. Avoid excessive physical activity and consider wearing an ankle brace to provide a good resting environment for the tendons.

To promote blood circulation and accelerate the healing of tendon tissue, you can apply local heat therapy and physiotherapy. If visiting a hospital is not convenient, you can do heat therapy at home. Use a hot water bottle to apply heat to the painful area for about 20 minutes, three times a day.

If the pain is severe, you can use topical analgesic creams like Voltaren to relieve symptoms.

Rest following the above recommendations for two weeks. If the pain persists, I recommend going to the hospital for a foot MRI scan to assess if further intervention is necessary.

Tendon injuries generally have a slower recovery process, so be patient and try these steps first.

I hope this helps!

11.

Symptoms and Duration: My mother has been experiencing general body pain for approximately 8 years.

Medical History and Medication: She has been taking traditional Chinese medicine and receiving acupuncture treatment.

Questions Requiring Answers: Are there any suitable medications or methods to alleviate my mother's back pain and enable her to lead a normal life?

**The doctor's answer on the website**

Based on the information you provided, it seems that your mother's condition may not be solely related to cervical and lumbar issues. It's possible that osteoporosis is a significant factor, especially considering she has been experiencing widespread body pain for 8 years. This type of systemic pain is likely due to a systemic condition rather than a localized problem. Therefore, I suggest scheduling a bone density test at the hospital, preferably a dual-energy X-ray absorptiometry (DXA) scan, which provides more accurate results. If severe osteoporosis is confirmed, it could explain her current symptoms.

Treatment for osteoporosis typically involves three main aspects:

Lifestyle adjustments: Encourage her to increase calcium intake by consuming foods rich in calcium, such as eggs, milk, lean meats, and fish. She should also engage in about an hour of outdoor activities in sunlight daily. Adequate vitamin D is essential for calcium absorption.

Medications: Long-term use of calcium supplements and vitamin D is usually recommended. Additionally, specific osteoporosis medications may be prescribed based on her condition. Common options include bisphosphonates like alendronate or intravenous zoledronic acid.

Physical therapy: Physical therapy sessions can help improve her symptoms. Treatments such as infrared therapy and other forms of physical therapy may provide relief.

As for her neck and back pain, it appears that she has mild disc protrusions but no significant nerve compression. Conservative treatments are suitable in this case:

Rest: Avoid prolonged sitting, standing, or activities that involve bending or heavy lifting. Use a cervical pillow for neck pain and consider using a lumbar pillow for back pain.

Physical therapy: Consult a physical therapist for exercises and stretches that can help alleviate her neck and back pain.

Medications: Non-prescription pain relief gels, like diclofenac gel, applied externally at the painful sites may provide relief. Oral medications like celecoxib, meloxicam, and methylcobalamin can be considered.

I hope these suggestions help improve her symptoms. However, please note that complete recovery may be challenging, especially regarding disc protrusions. It's crucial for her to continue protecting her spine by avoiding prolonged sitting, bending, and heavy lifting to prevent symptom recurrence.

If you have further questions or concerns, feel free to ask. I'll be happy to assist you.

12.

Hello, Doctor. Last night, while I was in bed (not long after getting into bed), I suddenly felt a dull pain in my left elbow joint, and this morning, there hasn't been a significant improvement. The discomfort in my elbow remains the same when I bend it or make a fist. Yesterday, I spent most of my day sitting at the computer and coding (although I did get up and move around frequently, similar to a regular workday). Over the past month or two, on weekdays, I've been going to the gym in the evening for a brief workout, which includes about ten minutes of running and some simple exercises for my back and chest. I typically go to bed around 12:30 AM. There doesn't seem to be any specific cause for this, so I wanted to ask the doctor what might be going on. Thank you, Doctor! Translated into English.

**The doctor's answer on the website**

I'm sorry to keep you waiting. Based on the information provided, your main symptom currently is left elbow joint soreness and discomfort for one day, with no significant changes in symptoms during elbow joint movement. From the information provided, your daily routine seems fairly regular, and you haven't engaged in any strenuous activities recently. If we rule out any obvious history of trauma, such as a collision or a fall, then the localized pain in your elbow joint is likely related to your extended period of coding in front of the computer yesterday.

Although you mentioned that you didn't sit for extended periods continuously and took breaks, coding work can still be quite intensive, and the duration required to complete tasks might not be short. I'm not sure if you have this level of work intensity every day. There are two possibilities here:

If you do this kind of work regularly, then it's possible that the chronic strain on muscles and ligaments due to prolonged computer use and upper limb activity is causing the pain, with yesterday's workload acting as a triggering factor.

If this level of work intensity is not common for you and it was a short-term (within a day) high-intensity upper limb activity, it might have caused acute soft tissue damage.

I recommend reducing any unnecessary high-intensity or repetitive upper limb activities in the near term, prioritizing rest. If the pain is significant, you can use topical diclofenac sodium gel to alleviate the symptoms. It's essential to note that if you experience numbness in the skin on the ulnar side of the forearm, numbness in the pinky and ring fingers, or restricted mobility, please consult an orthopedic outpatient department at the hospital promptly to rule out the possibility of cubital tunnel syndrome.

13.

Hello, from the information you've provided, here are your questions translated into English:

Symptoms and Duration: Hello, Dr. Wang. I had pain in my left ankle after running previously, but there was no swelling. It has been ongoing for over two weeks, and there has been some improvement in the past few days. I had sprained my ankle in 2012 and again in December 2021, but I recovered from both incidents. In between, I engaged in running and playing basketball without any discomfort.

Medical History and Medication: This week, I had an MRI, which showed a small cystic lesion on the lateral aspect of my left ankle. The doctor mentioned that this cystic lesion does not seem to be the cause of the pain and is not significant. They suggested that once the pain subsides, I can resume physical activities. I would like to understand more about this small cystic lesion and the possible reasons for the pain.

Questions to be answered:

What exactly is a small cystic lesion? Is it fluid accumulation between the joints?

How does this type of small cystic lesion form? Has it been present since the ankle sprains in the past or could recent physical activity have caused some damage (if so, what kind of damage)?

Will the small cystic lesion naturally disappear over time? And once it disappears, is there a possibility of it reoccurring?

If the small cystic lesion is an MRI finding, what is the source of my current pain? Is there inflammation, or is it caused by something else?

The MRI report mentions "T1WI low signal, PD fat suppression high signal." What does this mean?

**The doctor's answer on the website**

Hello, it's a small cyst within the bone, unrelated to joint effusion. As long as it doesn't affect the joint surface or damage the bone, it doesn't have any significant impact. The exact cause of these cysts is currently unknown, and they are not related to previous ankle injuries. Typically, they do not disappear on their own. These cysts are primarily imaging findings and do not have clinical significance unless they affect the joint or cause joint surface damage. The pain in the ankle might be due to ligament strain from an injury or inflammation of the fascia. The MRI description refers to the T1, T2, and fat suppression images, which are technical terms used in radiology.

I hope this clarifies things for you. If you have any more questions or need further information, please feel free to ask.

14.

Hello, I noticed pain on the inside of my right knee around early May. The day before, I may have run a few steps to catch a bus, but I wasn't running very fast. The pain has persisted for almost a month now. Currently, when I bend my leg outward, it causes a sharp pain. When I lie on my side, the inside of my knee also hurts, but it doesn't hurt when I'm still or walking normally. I've read reports about conditions like bone cancer, and I'm worried it might be something serious because this condition is common in people in their twenties. I hope the doctor can provide some answers regarding this one-month-long pain in the inside of my right knee.

**The doctor's answer on the website**

Based on the information you've provided, it's unlikely that you have a serious condition like bone cancer. The pain you've described in the inside of your right knee may be related to a minor injury, possibly from running to catch a bus or other activities. It's not uncommon for such discomfort to persist for several weeks.

I recommend that you consider visiting a healthcare professional for a thorough examination, including a possible MRI scan to determine the exact cause of your knee pain. The potential reasons for pain in this area may include a minor bone bruise, damage to the medial meniscus (a cartilage structure in the knee), or a minor ligament injury. Given that you don't experience significant pain during normal walking and that the pain is mostly triggered by specific movements or positions, it suggests that the injury may not be severe.

In the meantime, it's important to prioritize rest. Avoid activities that worsen the pain and consider using a knee brace during the day. Try to maintain different sleeping positions at night to minimize discomfort. Additionally, you may benefit from physical therapy, such as infrared therapy or ultrasound treatment, to aid in your recovery.

As for medication, you could consider using topical fluorouracil gel, oral pain relievers like ibuprofen (if not contraindicated for your specific situation), and other medications that your healthcare provider may recommend based on your diagnosis.

Please keep in mind that without a proper diagnosis, it's challenging to provide a precise treatment plan. Once you've received further evaluation and a diagnosis from a healthcare professional, you can tailor your treatment accordingly. Overall, don't be overly concerned, and focus on seeking professional guidance to address your knee discomfort effectively.

15.

Symptoms and Duration of Illness: My mother, who is 57 years old, experiences discomfort in her lower legs when standing for extended periods during the day. It's not exactly pain, but rather a feeling of discomfort. At night, when she lies down, she feels a dull pain in her right lower leg (see attached images). The pain isn't severe, but it prevents her from falling asleep, typically lasting until around 3 a.m. Occasionally, she also experiences lower back pain, with some localized swelling. When pressing on the affected area, it doesn't immediately return to normal. There are times when prolonged standing or sitting causes numbness in her hips, which extends down to her knees. These symptoms started around the Chinese New Year this year and have worsened over the past two months.

Medical History and Medication: In January of this year, she underwent an MRI, which showed a protrusion in her lower back. A recent evaluation by a doctor referred to it as a "protrusion." She was prescribed medication for promoting blood circulation and resolving stasis and also used topical plaster. However, there hasn't been a noticeable improvement in her pain despite taking these medications. Over the past month, she has been consistently engaging in lumbar stretching exercises, with each session lasting approximately 10-15 minutes. She has also avoided heavy activities and minimized actions involving squatting or bending.

Questions Needing Answers: Are there any further examinations that should be conducted? What medications can be taken to alleviate lower back and leg pain? What exercises can help reduce the pain?

**The doctor's answer on the website**

Based on the information you provided, your main symptom appears to be pain in your legs. During the day, it might be uncomfortable but not extremely painful, while it becomes more severe at night and can affect your sleep. This suggests that the symptoms are relatively serious. Initially, I thought it might be a problem with your lumbar spine, but you mentioned that you have already had a lumbar spine examination at the hospital, and the report indicated a disc protrusion. The doctor believes it could be a herniation. However, in this situation, even if it's a herniation, it may not be too severe, and it shouldn't necessarily be compressing a nerve. Additionally, even if there is nerve compression, the symptoms might be more prominent during daytime activities and relatively better at night, so this factor doesn't seem to fit entirely.

You also mentioned numbness from the hip to the thigh and knee when standing or sitting for extended periods, which may not necessarily be related to lumbar spine issues. It could be due to inflammation of the lateral femoral cutaneous nerve and might be related to prolonged sitting. The treatment for this issue primarily involves avoiding prolonged sitting and using physical therapies such as local heat application or infrared therapy lamps at the hospital. Typically, it can gradually improve, and you can consider using nerve-nourishing medications such as oral methylcobalamin and mazindol. So, there's no need to worry too much about this problem.

Furthermore, you mentioned swelling in your legs at times, and pressing on it leaves an indentation that doesn't immediately bounce back. This is a sign of pitting edema, which could be related to vascular issues. You should consider visiting the vascular surgery department of the hospital for a lower limb vascular color Doppler ultrasound, including arteries and veins. Additionally, you can consider having an electromyogram (EMG) for your lower limbs to check for any significant muscle or nerve issues. Considering your age, if you are postmenopausal, it's also worth investigating osteoporosis, which can sometimes be related to leg and lower back pain. You can consider a bone density test in the orthopedic department for a more comprehensive analysis of your condition.

If there are vascular issues in your legs, such as arterial plaque or blockages, or venous thrombosis, treatment options like medication or interventional procedures can be considered, which generally have good results. If the EMG shows minor issues, you can manage it with physical therapy, rest, acupuncture, and nerve-nourishing medications like methylcobalamin and mazindol, as mentioned earlier. The medications your doctor prescribed for lumbar disc protrusion may not be necessary if they haven't been effective.

If osteoporosis is detected and is significant, you should focus on a three-pronged treatment approach: 1) Increase intake of high-calcium foods like eggs, milk, lean meat, fish, and shrimp. Get plenty of sunlight daily to promote calcium absorption. 2) Take oral calcium and vitamin D supplements daily as the basic treatment for osteoporosis. 3) Consider using specialized medications for osteoporosis, such as oral alendronate sodium or intramuscular calcitonin. These treatments are generally effective.

In conclusion, I recommend that you undergo further examinations at the hospital to determine the root cause of your symptoms and receive appropriate treatment. If you cannot immediately access these tests, you can consider using medications to alleviate your symptoms temporarily. Regarding exercise, at this point, I would advise against strenuous activities. Focus on rest and consider light walks in the sunlight.

16.

Symptoms and Duration of Illness: Symptoms: When I bend my left wrist backward and support it with my hand, there is significant pain at the wrist joint. Sometimes, when I bend my left hand forward, there is a protrusion of pain at the wrist, and pressing on that area causes mild discomfort. However, when I fully extend my left hand and press on the painful area, there is no pain. These issues are only present in my left hand, and my right hand does not experience these problems.

Duration: It has been over a year since these symptoms started. Initially, I had some pain, but I didn't pay much attention to it as I wasn't engaged in any physical activities.

Specific Presentation: When attempting to do push-ups or any other activity that requires supporting my body weight with my hands, my left hand is unable to bear the weight and can only form a fist.

Medical Consultation and Medication History: I haven't sought any treatment or undergone relevant medical examinations for this issue.

Questions to be Answered: How should I treat the wrist pain issue? What medications, if any, are needed? How long will it take for the pain to be completely resolved so that it no longer bothers me?

**The doctor's answer on the website**

Hello, based on the medical history and clinical experience, the current discomfort in the wrist is initially considered to be more likely due to wrist joint arthritis or chronic ligament damage. Secondly, it could be caused by tenosynovitis. Overuse, excessive frequency, excessive force, repetitive trauma, regular vigorous exercise, or frequent heavy physical labor can contribute to these conditions. You can visit the orthopedic department of the hospital for a wrist magnetic resonance imaging (MRI) examination to assist in diagnosis. For now, you can consider conservative treatment for approximately one month to promote recovery. After a re-evaluation with an MRI, if the condition is less severe, you should consider a rest and recovery period of 2-3 months.

Treatment recommendations:

Temporarily reduce excessive local activities and weight-bearing, wear a wrist brace, and rest more. The better the rest, the faster and better the recovery.

Apply local heat packs to the painful area to improve circulation. Apply a warm towel or hot water bag for about 2 times a day, each time for about 30 minutes, to expedite symptom relief.

You can apply Fluibiprofen gel or Fastum locally to the painful area to alleviate symptoms. Oral medications such as Piroxicam or Celecoxib can assist in reducing inflammation and pain.

If available, you can use an infrared lamp to irradiate the painful area. Infrared lamp irradiation (available for purchase online, approximately $100) can be done approximately twice a day, each time for about 30 minutes, at a distance of 30 cm. If you don't have an infrared lamp, follow the other treatment recommendations.

Each treatment cycle is 4 weeks. Stay consistent with the treatment, and your symptoms should improve relatively quickly.

17.

After playing badminton on the 11th, my left little toe became red, swollen, and painful. I took amoxicillin for three days, and it seemed to improve. However, after playing badminton again on the evening of the 14th, it became extremely painful the next day. I started receiving intravenous treatment with clindamycin on the 16th and continued with penicillin on the 17th and 18th, but I haven't noticed any improvement. The area under the little toe is still very painful and swollen.

**The doctor's answer on the website**

Based on the information you provided, it seems that your current toe pain may be caused by gout. Given that the pain appeared after playing badminton and there doesn't seem to be a clear external injury during the activity, overexertion during the exercise could have triggered a gout flare-up. While you may not have had gout before, I believe you haven't had your uric acid levels checked. In your current condition, with symptoms characterized by redness, swelling, and severe pain, gout should be considered. Although acute infection is also a possibility, since antibiotics haven't shown significant improvement, I personally believe that the likelihood of infection is relatively low. However, when you visit the hospital, you should consider getting a complete blood count, erythrocyte sedimentation rate, C-reactive protein, and uric acid levels checked. If your blood counts are not elevated but uric acid levels are high, gout is likely the cause of your symptoms. Nevertheless, there's no need to worry, as gout can be effectively treated.

For the next steps in treatment, I'll provide you with some recommendations that you can consider for a successful recovery.

Rest is crucial in your case. With localized symptoms of redness, swelling, and pain, excessive movement and exercise should be avoided. Rest is essential to allow the local inflammatory response to subside. It's important to note that while you initially felt some relief after taking anti-inflammatory medication, your condition didn't fully recover, and symptoms worsened after exercising again. Therefore, it's highly advisable to prioritize rest. If necessary, you can use crutches to minimize pressure and irritation on the affected area.

Cold compresses, rather than hot ones, should be applied locally when you experience significant symptoms. Since you are in the acute inflammatory phase, cold compresses can effectively control local inflammation and aid in your recovery.

I recommend taking oral colchicine tablets. This medication has strong anti-inflammatory properties and is specifically used for gout. Typically, you should see noticeable improvement within 3-5 days of oral administration. Continued use should gradually lead to a full recovery. After your symptoms have subsided, we will need to evaluate your uric acid levels. If they are high, you may need to take uric acid-lowering medication, such as allopurinol. You will need to follow up with regular blood tests, and once your uric acid levels drop below 300, the medication can be discontinued.

Dietary control is essential. You should increase your daily water intake significantly and consider drinking soda water, as this helps with uric acid excretion. Additionally, you should avoid or limit the consumption of high-purine foods, such as alcohol, seafood, hotpot, beef, lamb, soy products, and animal organs.

So, as long as you get a proper diagnosis at the hospital and follow these recommendations for treatment, especially if it turns out to be gout, the prognosis should be quite positive, and there's no need to worry. Currently, you're using antibiotics, but if the issue is not an infection, these medications are unlikely to be effective.

18.

Symptom Description: Pain at the second joint of the fingers and toe joints without swelling. Specifically, I experience discomfort when gripping things for an extended period, such as holding chopsticks while eating or carrying packages, with pain setting in after about five to six minutes, requiring some movement and rest for relief. At night, if I clench my hand into a fist for an extended period while sleeping, it causes pain and wakes me up. I don't experience pain during regular activities.

Duration of Illness: Approximately two weeks.

Medical Examination: I haven't sought medical attention or taken any medication because the pain is not continuous. There are no visible deformities or swelling, and normal finger movement is not affected. The most noticeable symptoms occur when I exert force with my fingers or toes, leading to a sensation of pain and stiffness, which can be alleviated by stretching. At night, maintaining a specific position with my fingers or toes also causes pain and stiffness.

I decided to seek medical advice because I noticed this pain sensation occurring in my elbow and knee joints this morning. The pain is very brief, so I didn't immediately identify any specific triggers.

**The doctor's answer on the website**

In this case, I would recommend the following:

Consult a rheumatologist to rule out the possibility of underlying systemic diseases such as rheumatoid arthritis, lupus, gout, and other rheumatic conditions.

Consult a hand surgeon to investigate the possibility of tenosynovitis.

Based on your description, the symptoms appear to be more aligned with the first possibility. In clinical practice, we generally start by considering one disease that could explain all the symptoms. The likelihood of a simple multi-joint tenosynovitis is relatively low.

19.

Symptom Description: There is pronounced redness and swelling around the ankle, and when muscles are engaged or pressure is applied, there is significant pain that subsides when the pressure is released. There hasn't been a significant sprain prior to the appearance of these symptoms. (Additionally, a few days ago, there was a glass scratch on the toe, which has since scabbed and is almost healed. Could this be related to the way you walked while injured?)

Duration of Illness: Two days.

Medical Examination: Currently unable to seek medical attention due to lockdown and quarantine conditions in Shanghai.

Medication: Fluibiprofen gel patch.

**The doctor's answer on the website**

Based on the information you provided, I believe that your current condition in the foot is likely an acute inflammatory response, with the most probable cause being gout. I don't know if you have had gout before or if you've ever had your uric acid levels checked. If your uric acid levels are high, it could be the cause of this condition. Since you mentioned no specific injury before the onset of symptoms, it is less likely related to the scratch on your toe, as infections from such scratches would typically affect the immediate area first and then potentially spread. Therefore, my personal assessment is that it could be gout.

If you are unable to visit a hospital for consultation, you can follow these recommendations for managing this condition:

Rest: Since you are experiencing pain, try to limit your physical activity and elevate your foot as much as possible. Resting will aid in your recovery.

Medication: You can apply flurbiprofen gel locally, and the gel patch you mentioned is also suitable. Additionally, take oral indomethacin tablets. This medication is effective for reducing acute inflammation caused by gout. You can purchase it from a pharmacy.

Cold Compress: Apply cold compresses locally. Avoid heat compresses during the acute phase. Apply cold for 5-10 minutes every 4 hours.

Dietary Control: Avoid or reduce the intake of alcohol, seafood, hotpot, beef, lamb, soy products, organ meats, and other similar foods both in the short term and long term. Drink plenty of water to promote uric acid excretion.

You can follow this treatment plan for now, and I believe it should yield positive results. Don't worry too much. If you can, consider checking your uric acid levels in the future. If they are elevated, you may need to take oral uric acid-lowering medication such as allopurinol. After a period of treatment, you can undergo a follow-up test, and if your uric acid levels are below 300, you can discontinue the medication.

20.

Hello, I have been experiencing difficulties in moving my right elbow joint since yesterday. There is a persistent pain at a specific location in the joint, making it difficult for me to raise or bend my arm without severe pain. I have a history of gout, but my uric acid levels have been controlled at around 300, and I have been taking uric acid-lowering medications regularly. I'm not sure if this is gout affecting my elbow joint or if it's a new form of arthritis in the elbow. Currently, I am taking medications for gout, such as slow-release sodium diclofenac and other conventional uric acid-lowering drugs. My elbow joint feels warm, but there isn't significant swelling. When I touch it, there is a specific point that is very tender. Due to the current lockdown measures in place in some neighborhoods around Beijing, I am hesitant to visit a hospital. Therefore, I am seeking your help here for advice.

**The doctor's answer on the website**

Hello, I am a doctor from the Orthopedics Department at SUDA First Affiliated Hospital. Based on your description, along with the localized warmth, it seems that gout affecting the elbow joint is more likely. Recommendations:

Drink plenty of water, ensuring a daily urine output of over 2000ml.

Consider switching to an anti-inflammatory and analgesic medication, such as oral colchicine.

You should definitely control your diet and reduce consumption of foods rich in purines. Ideally, it would be best to visit the outpatient department, get an X-ray, and have your blood tested to check your uric acid levels.

21.

I am in home quarantine, and for no apparent reason, the large joint connecting the base of my left big toe to the sole of my foot has started to hurt. I have never experienced a similar situation before. I soaked my feet in cold water barefoot about 10 days ago. Could this be acute arthritis?

**The doctor's answer on the website**

Based on the information you provided, I don't believe that the localized pain in your big toe joint is acute arthritis. It's unlikely to be related to soaking your feet in cold water 10 days ago, as the pain would typically occur on the same day or within a day or two afterward, not with a 10-day gap. In general, when a young male suddenly experiences pain in the big toe joint, the most likely cause is gout. Since you haven't been engaging in any physical activity during your quarantine, it's not likely to be a result of strain or injury.

You should consider your recent dietary habits. Have you consumed foods that are known to elevate uric acid levels, such as seafood, hotpot, beef, lamb, soy products, or organ meats? Additionally, given your relatively higher body weight, it's worth noting that overweight individuals are more prone to gout. Gout is fundamentally a metabolic disorder.

Considering that you currently can't visit a hospital for blood tests, I suggest you initially treat it as a potential gout episode. The primary focus of treatment is rest, so try to minimize walking and avoid putting weight on the affected foot. Dietary control is essential; avoid the mentioned foods and continue to do so in the future to reduce uric acid production, which will aid in your recovery and prevent future flare-ups. Make sure to drink plenty of water daily, preferably with soda water if available, or plain water if soda water is not accessible. Aim for at least two liters of water daily to promote uric acid excretion.

If the pain is severe, you can use medication to manage your symptoms. The recommended medication is colchicine. If that's not available, other pain relievers like ibuprofen or acetaminophen can also be used. Typically, within 3 to 5 days or a week of using these medications, you should experience significant relief from your symptoms.

Once your symptoms have improved, it's advisable to check your blood uric acid levels. If they remain high, you may need to continue uric acid-lowering medications like allopurinol. Regular uric acid checks are necessary, and you can stop the medication when uric acid levels drop below 300. With good dietary control, the risk of recurrence is low, so the prognosis for gout is generally favorable. Don't worry too much about it.

22.

Hello, doctor. My right Achilles tendon started feeling uncomfortable four days ago, with a slight discomfort while walking. I applied Yunnan Baiyao, but it hasn't improved. Yesterday afternoon, it started to hurt more when walking, and by the evening, it became quite painful. The pain occurs even when I'm not moving, with occasional throbbing sensations. Strangely, when I stand up without moving, it doesn't hurt. I drank some alcohol yesterday at lunchtime. Can you please tell me the cause and how to relieve the pain? I noticed tenderness on the outer side of my heel last night. I've confirmed that the ankle bones aren't painful. My knee and other joints are also not painful. There's no change in temperature when I touch the skin in that area. The pain is more pronounced when I flex my ankle, and lifting the top of my foot causes significant pain. Oddly, there isn't much pain when I raise my toes. I have a flight today, and during the flight, the area became swollen, and walking is painful. Standing with pressure also causes pain. Can I apply medicinal alcohol now for adjunctive treatment, and do you have any suggestions for acute pain relief medications?

**The doctor's answer on the website**

I'm sorry for the delay. Based on the information you provided, the pain in your right Achilles tendon area could have two potential causes:

Physical Strain or Injury: If you engaged in intense physical activity, prolonged walking, or strenuous labor before experiencing the discomfort, it's possible that you have incurred some strain or inflammation in the Achilles tendon or the adjacent bursa. In this case, rest is essential. Refrain from any strenuous activities, including walking or physically demanding tasks for 1-2 weeks. You can also consider using topical pain relief gels or ointments like Flurbiprofen gel for localized relief.

Gout: If you haven't engaged in any strenuous activity before the onset of pain, but you did consume alcohol recently, gout is a possibility. Gout is more common in young men and typically affects joints, including the Achilles tendon area. It is associated with high levels of uric acid in the blood. To confirm or rule out gout, it's advisable to have a medical evaluation and blood tests to check your uric acid levels.

For both scenarios, it's crucial to rest the affected area to reduce strain and promote recovery. If you suspect gout, it's essential to control your diet, avoiding high-purine foods such as alcohol, seafood, red meat, beans, and organ meats. Drink plenty of water, preferably including soda water, to facilitate uric acid excretion. You can also use over-the-counter pain relief medication, such as Ibuprofen or Naproxen, for short-term relief.

If the pain persists or worsens, or if you receive a diagnosis of gout with elevated uric acid levels, your healthcare provider may prescribe specific medications like Allopurinol or Febuxostat to manage gout and prevent future attacks. Long-term dietary and lifestyle modifications are important for gout management.

In the case of swelling, you can use ice packs intermittently for 15-20 minutes to reduce inflammation. If you plan to take a flight, try to keep your leg elevated during the flight to minimize swelling. However, it's crucial to consult with a healthcare professional for a proper diagnosis and treatment plan, especially if you suspect gout or if the pain persists.

23.

Hello, I am 29 years old, male. My right knee started to hurt suddenly yesterday. When I bend it, it hurts a lot, and it feels tight with some swelling. Walking and driving are inconvenient now, and I can only walk with my right leg straight. I have no history of high uric acid, no medical history, and no allergies. I'm not sure if it's related to eating seafood last night. I applied some topical pain relief oil last night, so my right knee appears a bit red, but there hasn't been any improvement as of this morning.

**The doctor's answer on the website**

Based on the information you provided, I consider that the pain in your right knee may be related to gout. Sudden knee pain in young males, especially without any obvious cause or triggering factors, such as vigorous exercise, physical labor, or extensive walking, should raise suspicion of gout. Although you didn't have any prior symptoms of gout, you may not have undergone specific tests for high uric acid levels. If you've been consuming foods with high purine content in the past, such as beer, seafood, hotpot, beef, lamb, soy products, organ meats, etc., your uric acid levels might have been elevated, leading to a condition called hyperuricemia. When uric acid levels become significantly high, uric acid crystals can deposit in joints or nearby soft tissues, triggering acute inflammatory responses and causing pain and swelling. Given these considerations, it's likely that your current knee pain is due to gout.

I recommend that you visit a hospital's orthopedic department today. First, have a doctor examine the painful area in your leg. Second, the doctor will likely recommend a blood test to check your complete blood count and uric acid levels. If your blood uric acid level is significantly elevated, it will confirm that your current symptoms are indeed caused by gout. However, there's no need to worry, as your pain has not been present for long, starting only yesterday. With proactive treatment, you can expect a swift recovery.

Here are some treatment recommendations:

Rest: Avoid activities that aggravate the pain. Try to keep your leg immobilized and avoid bending it as much as possible. Consider taking some time off work to rest at home.

Cold Compress: Since the area may feel warm to the touch due to acute inflammation, use an ice pack or cold compress for about 20 minutes at a time, every 4-5 hours. This will help reduce pain and swelling and aid in your recovery.

Medications: Apply topical pain relief ointment like "扶他林软膏" locally. Additionally, take oral medications such as "依托考昔片" and "迈之灵," which are highly effective in treating acute gout attacks and reducing pain and inflammation.

Diet Control: Avoid foods high in purines, such as beer, seafood, hotpot, beef, lamb, soy products, organ meats, and sugary beverages. Hydration is essential, so drink at least two liters of water daily, preferably including soda water, to facilitate uric acid excretion.

Uric Acid Lowering: If your uric acid levels are significantly elevated (e.g., above 500), after the initial pain relief, your doctor may prescribe uric acid-lowering medications like "苯溴马隆" or "非布司." You will need to take these medications for about two weeks and have a follow-up blood uric acid test. If your uric acid levels drop below 300, you can discontinue the medication; otherwise, you may need to continue taking it.

Once you confirm your diagnosis at the hospital and follow these recommendations, your recovery should be swift. Don't worry too much, and with good dietary control, the likelihood of recurrence is generally low.

24.

My grandmother is 84 years old this year and is experiencing profuse sweating, fever, pain, and difficulty sleeping at night. She can only fall asleep by taking sleeping pills. She went to the hospital for an examination, and they said it's osteoporosis. There isn't any specific medication for treatment, just calcium supplementation and exposure to sunlight. Are there any other treatment options available?

**The doctor's answer on the website**

Hello, regarding the diagnosis of "osteoporosis" based on the bone density test results:

Osteoporosis is a systemic bone disease characterized by reduced bone density, compromised bone quality, and increased bone fragility, leading to a higher susceptibility to fractures. In elderly individuals, the decrease in sex hormones stimulates bone-resorbing cells while inhibiting bone-forming cells, resulting in decreased bone mass. Furthermore, aging is associated with reduced nutrient absorption and organ function, potentially leading to vitamin D deficiency and chronic negative calcium balance, contributing to decreased bone mass and quality. Women have a higher risk and severity of osteoporosis compared to men, especially after menopause.

The main symptoms of osteoporosis include back pain, which is often observed in the lumbar area, as well as generalized bone pain. The pain is usually diffuse and lacks specific focal points. Pain tends to worsen with changes in posture, prolonged walking, nighttime, or weight-bearing activities and may even lead to limited mobility.

Systemic treatment for osteoporosis can alleviate symptoms and reduce the risk of fractures. Generally, osteoporosis treatment primarily involves medications:

Calcium Supplements: Calcium serves as the raw material for bones. Choose calcium supplements that are readily absorbed, such as "钙尔奇" or liquid calcium. The requirements for calcium supplements are not very stringent; common options suffice.

Active Vitamin D: These promote calcium absorption. Common options include calcitriol and alfacalcidol. The choice of medication and dosage may depend on your bone density, typically ranging from 1 to 4 tablets daily. You may also receive a monthly injection of vitamin D2 (15mg) at the hospital, with a treatment duration of approximately six months.

Bisphosphonates: These medications inhibit the activity of bone-resorbing cells. There are various types of bisphosphonates, including domestic and imported options. A commonly used medication is alendronate sodium (taken once a week for over three years). Alternatively, you can opt for the more effective intravenous zoledronic acid (marketed as "密固达"), an imported bisphosphonate. It costs around 3,000 RMB per injection, administered once a year for three consecutive years, and is currently the best bisphosphonate available in China.

Calcitonin: This medication also inhibits the activity of bone-resorbing cells and is administered via injection. Calcitonin helps mobilize calcium from the bloodstream to deposit it in the bones, increasing bone density. It can also provide relief from osteoporosis-related pain. If pain is pronounced, consider using it. However, calcitonin is only available as an injection and should be administered in a hospital. It may be necessary to simultaneously receive intravenous calcium supplementation since it can potentially lead to hypocalcemic seizures.

Osteoporosis is a systemic disease requiring systematic treatment. Single medications may not yield optimal results. Nonetheless, with consistent treatment, it is possible to effectively reduce the risk of fractures. Fractures are the most dangerous complication, so it's essential to take precautions against falling.

I hope this information is helpful. If you have further questions or need additional information, please feel free to ask.

25.

Hello doctor, the sole of my right foot, specifically the arch, is painful. There are calluses at the painful site, and it hurts when I walk. The pain is alleviated when I'm not walking. How should I treat this?

Duration of condition: One week

**The doctor's answer on the website**

From the information you provided, this condition is typically consistent with plantar fasciitis, which is a chronic inflammation of the plantar fascia on the sole of the foot due to repeated stress. It doesn't hurt when you're not putting weight on it but becomes painful when walking. I recommend wearing cushioned shoes and avoiding vigorous physical activity when experiencing pain. You can soak your feet and apply medicated patches. If the pain is severe, you can use anti-inflammatory and pain-relieving medications like Celecoxib or Etoricoxib. In some cases, localized pain relief methods may be necessary.

26.

Hello, regarding your mother's condition, the appearance of a soft, fluid-filled lump on the wrist without a history of insect bites or significant pain could be due to various causes. It's essential to consider possibilities like ganglion cysts, lipomas, or other benign growths. While there may not be immediate pain, these growths can sometimes cause discomfort or complications over time.

I recommend that your mother consult a medical professional for a proper evaluation. The doctor can perform a physical examination, possibly order imaging tests like an ultrasound or MRI, and determine the most appropriate course of action. Treatment options may include observation, drainage, or surgical removal, depending on the diagnosis.

Please seek medical advice to ensure an accurate diagnosis and appropriate management for your mother's condition.

**The doctor's answer on the website**

Hello, based on the information provided, it seems that the lump on your mother's wrist may have two possible causes.

Firstly, it could be a tenosynovial cyst, which is a fluid-filled sac that develops in the tendon sheath due to repetitive strain or inflammation. This can occur in individuals who engage in activities that involve frequent wrist movement or strain. Initially, there may be some discomfort or aching in the wrist, but once the cyst forms, it may not cause significant symptoms. It's essential to have a medical evaluation to confirm the diagnosis. I recommend taking your mother to the orthopedic department of the hospital tomorrow for an examination and a possible ultrasound to determine the nature of the lump. If it is a tenosynovial cyst, it may not require immediate intervention but should be protected with a wrist brace or support for about a month. Additionally, applying warm compresses and topical anti-inflammatory ointments can help reduce inflammation and promote cyst absorption.

Secondly, it could be a subcutaneous lipoma, which is a benign fatty lump that can develop under the skin. These are more common in older individuals, and while they may not initially cause pain, they can grow over time. Again, a medical evaluation and possibly an ultrasound can confirm the diagnosis. If it is a lipoma and small in size, it may not require immediate treatment but should be monitored for any changes.

In either case, it's essential to seek medical advice to determine the exact cause and appropriate management for your mother's condition. Please do not worry excessively, as both tenosynovial cysts and lipomas are generally benign and can be managed effectively with the right approach.

27.

Hello! I've been experiencing mild pain in the finger joints for the past two days, which is affecting my ability to perform tasks. This has occurred a couple of times before, and it eventually resolved on its own. There are no visible signs externally. I'm not sure if it's related to the weather as the temperature here is quite low, around 2-3 degrees Celsius.

I haven't sought medical attention or taken any medication for this issue. My main concern is finding relief from the pain.

**The doctor's answer on the website**

Hello, based on the information you provided and the photos you uploaded, it appears to be synovitis or osteoarthritis in the joints. I would recommend applying warm compresses to the affected hand twice a day for 20 minutes each time. For discomfort in the hand, you can use topical pain relief ointment like "扶他林软膏" for anti-inflammatory and pain relief treatment. You can also take oral anti-inflammatory and pain relief medication like "塞来昔布." It's advisable to rest more during the treatment period, avoid exposure to cold, and refrain from excessive exertion.

28.

Duration of the condition: 2-3 years Symptom description: My grandmother, who is 72 years old, has swelling and discomfort in the inner part of her knee. Medication history: No medication has been taken.

**The doctor's answer on the website**

The popliteal cyst (also known as Baker's cyst) is a benign condition. It is often secondary to arthritis, which is common in elderly individuals. In this case, the cyst has grown to a size that is not considered small. If it is causing pain or significantly impacting joint mobility, such as restricting flexion and extension and affecting the quality of life, I would recommend considering active intervention. Active intervention involves draining the fluid accumulated within the cyst. Since the ultrasound results indicate that the cyst is located just under the skin, you can use a simple procedure with a syringe to aspirate the cyst's fluid. If you have concerns about safety, it's also possible to perform the aspiration guided by ultrasound, which is a safer approach. This procedure is relatively straightforward and does not require advanced technical skills. Most hospitals above a certain level should be able to perform it. Therefore, if the cyst is causing significant discomfort and affecting the quality of life, it is worth considering this procedure. Without intervention, conservative treatments like medication or physical therapy may not be very effective, especially given the size of the cyst. These conservative methods are more suitable for smaller cysts.

29.

Based on the information provided, it appears that your child, who is eight years old, has a swollen knee, but there is no pain, and it does not affect walking. The recent physical activities include approximately twenty days of swimming, and yesterday, they played at a trampoline park. This morning, you noticed the swelling in the knee, but your child doesn't seem to feel any discomfort.

The swelling has been present for one day, and there hasn't been any medication or hospital examination.

**The doctor's answer on the website**

Based on the information you provided, it seems that your child's swollen knee may not be a significant issue. The swelling is observed without any pain or functional impairment, and it may be related to recent intense physical activities, especially trampoline jumping, which can exert considerable stress on the knee joint. Falls or kneeling during such activities could potentially lead to soft tissue injuries, resulting in swelling. Another possibility is the presence of some joint fluid, which could be diagnosed as synovitis. However, since there is no pain involved, the issue may not be of great concern.

If you are unsure or concerned, it's advisable to schedule an appointment with an orthopedic specialist at a hospital. A physical examination by the doctor can help determine whether there is an issue with the joint, and they may recommend an ultrasound to further assess the condition. In the absence of pain, there is no need for medication. Rest and gentle care, such as applying warm compresses and elevating the legs during rest, can be helpful. Additionally, using a knee support while engaging in physical activities can provide extra protection.

Overall, it appears that your child's condition is not severe, and children typically do not hide symptoms of pain. Therefore, you can monitor your child's activities and seek medical attention if you deem it necessary, although the need for immediate intervention seems relatively low.

30.

Symptom description: Since September last year, there has been swelling and pain near the Achilles tendons of both feet, especially noticeable when going downstairs or after sleeping. Last year, I spent a long time walking and standing during a Xinjiang aid mission, with temperatures dropping to more than minus 30 degrees Celsius in winter and exceeding 40 degrees Celsius in summer. Over the course of a year, I have consistently experienced swollen and painful feet, and my knees have also been uncomfortable, making it difficult to exert force. Resting for a few days helps alleviate the symptoms, but as soon as I start walking again, the discomfort returns. I had no health issues before going to Xinjiang. Could you please tell me what condition this might be, whether medication is necessary, and whether I should see a doctor? I haven't visited a hospital or taken any medication for this issue so far.

**The doctor's answer on the website**

Based on the information you provided, I believe that the pain in your ankles may be caused by synovitis. As for why this condition has occurred, it is likely related to the increased physical activity from your daily walking and standing during your aid mission in Xinjiang. You mentioned that you didn't have this issue before, which suggests that your activity level may not have been very high previously. Therefore, suddenly engaging in a lot of walking can lead to synovitis, causing pain not only in your ankles but also in your knees.

However, since you haven't visited a hospital for a proper diagnosis, this is a tentative assessment. If you are unsure or concerned, it's best to consult a doctor. You can consider getting a magnetic resonance imaging (MRI) scan of your ankles. If the MRI shows fluid accumulation or signs of inflammation, it can help confirm the diagnosis of synovitis.

Nevertheless, you don't need to worry too much. Synovitis can be managed and treated effectively. As you mentioned, resting for a period of time helps alleviate the symptoms, but they return when you resume walking. Therefore, rest should be the primary focus of your treatment. If possible, consider adjusting your work or taking a leave of absence for a while to rest. Adequate rest is crucial for your recovery. You can also consider using ankle braces or supports when walking to provide additional protection and support for your ankles.

Additionally, I recommend soaking your feet in warm water daily or using a hot water bag to apply heat to your feet, especially during cold winter days. Keeping your feet warm is essential. If feasible, you may also want to explore physical therapy options at a hospital, such as infrared therapy, which can greatly benefit your recovery.

Finally, medication is likely to be part of the treatment plan. Since you've been experiencing these symptoms for some time, I suggest applying Voltaren gel externally to the affected area after each hot compress or physical therapy session. You can also consider taking oral medications like Celebrex and Mobic, as they should aid in your recovery.

In summary, it appears that there is an issue with your ankles, but it doesn't seem to be very severe. With proactive treatment and care, there is a good chance that you can gradually return to normal. Don't worry too much, and make sure to consult a medical professional for a thorough evaluation and personalized treatment plan.

31.

Hello, doctor. I am a 22-year-old female, and I have recently experienced sudden knee joint pain. I don't experience pain when walking normally, but I cannot squat deeply, use the toilet while squatting, or walk downstairs without mild discomfort. I haven't engaged in strenuous exercise recently, but I did ride a bicycle for a couple of days. Occasionally, I do ride a bike as part of my daily routine. Could you please tell me if this is a serious issue, whether it can heal on its own, or if I need to go to the hospital for a comprehensive examination?

Additional information:

I have never experienced similar symptoms before.

I cannot complete deep squats due to the pain.

Currently, the joints appear to be similar to their usual state, with no redness or other symptoms. There is a slight sensation of coolness in the joints, but it disappears with adequate warmth. During cycling, my joints shouldn't have been exposed to cold, as I was dressed warmly. I don't experience joint fatigue or stiffness while walking normally, and there is no stiffness in the morning. The joint symptoms all seem quite similar, possibly because they only appeared yesterday.

**The doctor's answer on the website**

Hello, thank you for your patience. I carefully reviewed the information you provided. Based on the current situation, with no previous similar symptoms and sudden knee joint pain, especially during knee flexion, it's possible that you may be experiencing knee synovitis.

The synovial membrane is a thin layer within the knee joint that has several functions, including lubrication, nourishment, and protection of the joint. It contains rich blood vessels and secretes synovial fluid, which lubricates the joint surfaces, reduces friction, and dissipates heat generated during joint movement.

Synovial fluid, which is found within the joint cavity, is formed by filtration of plasma from tiny blood vessels beneath the synovial membrane. Additionally, synovial lining cells produce hyaluronic acid, which, together with synovial fluid, lubricates the joint and nourishes the cartilage. Normally, there is a minimal amount of synovial fluid within the joint cavity.

In some cases, acute trauma or chronic overuse can lead to damage to the synovial lining in the knee joint, resulting in tissue swelling, congestion, increased exudate, and joint effusion, leading to an inflammatory response. Additionally, exposure to cold weather can trigger synovitis. Knee synovitis is known to be sensitive to cold weather, and pain can worsen with cold temperatures. In other words, cold exposure can easily trigger synovitis. Although you've been mindful of keeping warm, the occasional sensation of coolness in your joints suggests that synovitis is a possibility.

The knee joint lacks the protective muscle and fat tissue found in the calf and thigh regions, making it more susceptible to heat loss, especially since it is essentially a "skin-and-bone" area. Therefore, local heat can dissipate easily, and insulation is less effective, causing the temperature in this area to be lower than in other parts of the body. Additionally, joint sensitivity is often lower than in other areas. Traditional Chinese medicine also associates the entry of cold and dampness into the joint cavity with certain conditions. Furthermore, exposure to cold can alter the joint microenvironment, leading to symptoms.

Considering your current situation, synovitis seems to be a likely possibility. However, since the time frame is relatively short, I recommend starting with symptomatic treatment and monitoring the progress of your symptoms. Here are some specific recommendations:

Reduce walking distances and prioritize rest, while avoiding knee flexion when lifting objects.

Apply warm compresses around the knee joint twice a day, each time for about 30 minutes, with a water temperature of approximately 40°C. Warm compresses can improve blood circulation and facilitate the absorption of fluid.

Pay attention to keeping your knee joint warm, especially as the weather gets colder, to prevent exposure to cold temperatures.

You can consider taking some over-the-counter anti-inflammatory and pain-relief medication. I'm providing a prescription for your reference.

32.

Symptom description: Initially, I woke up yesterday feeling like I had twisted my right big toe (possibly due to exercise). Last night, when I walked downstairs to have dinner, I noticed it was quite painful. Later, I used an ice pack to apply cold compress. This morning, the condition worsened significantly. The entire right foot, including the big toe, has become swollen. I cannot even fit it into slippers, and there is intense pain whenever there is pressure.

Duration of symptoms: Two days.

Hospital examination: None.

Medication: None. I recently received the COVID-19 vaccine and am unsure if I can take pain relievers.

Additional information:

There is a history of right ankle injury, with two previous instances of spraining it while playing sports. I don't wear tight shoes as my feet have some flesh, and I tend to choose shoes one size larger.

This level of severity has not occurred before. It typically involved the ankle only, but now the entire foot is swollen.

Recent physical activity has been moderate, with little exercise during the day. There have been 2-3 instances of non-strenuous sexual activity. My feet may have been exposed to cold, and I forgot to cover myself while using air conditioning.

The night before the symptoms appeared, I consumed around 700ml of beer. I have been eating fresh beef in my diet recently.

The pain is characterized by a throbbing and aching sensation when sitting, which transforms into sharp pain when standing or walking (applying pressure). At times, it is difficult to endure.

P.S.: Can I take pain-relieving medications (such as ibuprofen) after receiving the Sinovac COVID-19 vaccine on Monday?

**The doctor's answer on the website**

Thank you for providing a detailed medical history and symptoms. Based on your medical history and symptoms, there is a high suspicion of gout. It's important to clarify that gout is not necessarily caused by consuming seafood, and not everyone who consumes seafood will develop gout. I will provide a detailed explanation below.

Gout is a group of syndromes characterized by elevated levels of uric acid due to disturbances in purine metabolism, leading to the deposition of urate crystals in various tissues. Gout can be primary or secondary and is often associated with factors such as obesity, alcohol consumption, hypertension, hyperglycemia, and the excessive consumption of purine-rich foods and medications.

Gout typically affects joints such as the first metatarsophalangeal joint (big toe), dorsum of the foot, ankle joint, heel, knee joint, wrist joint, fingers, and elbow joint. The main symptoms of gout include redness, swelling, heat, and intense pain in the affected area. The pain is described as a tearing, sharp pain that is not related to movement, although it can be exacerbated by activity. Gout-related pain often occurs at midnight or in the early morning.

From the images you provided, the extensive swelling and the location of severe pain you mentioned, particularly in the first metatarsophalangeal joint, strongly suggest an acute gout attack. Given your recent history of alcohol consumption, the diagnosis is relatively clear.

You can consider the following treatment options:

Oral non-steroidal anti-inflammatory drugs (NSAIDs) for pain relief. You can also apply topical diclofenac gel to alleviate local pain. Additionally, you can take colchicine orally to reduce swelling.

Oral allopurinol can be taken to promote uric acid excretion. The usual dosage is 0.5–1 mg (1-2 tablets) every 1–2 hours until the symptoms improve, with a typical treatment dose of 3–5 mg (6-10 tablets) within 24 hours. Do not exceed 6 mg (12 tablets) in 72 hours. After a 72-hour break, take 0.5–1.5 mg (1-3 tablets) daily, divided into multiple doses, for a total of 7 days.

Avoid high-purine foods (such as animal organs, seafood, meat broth, dried peas, etc.) in your diet. Ensure adequate daily water intake (2000–3000 ml). Quit smoking and limit alcohol consumption (especially beer and spirits). Exercise regularly to control your weight. Increase the intake of alkaline foods (such as bananas, watermelons, pumpkins, cucumbers, strawberries, apples, spinach, radishes, green beans, lotus roots, and seaweed).

Elevate the affected limb, especially when sleeping at night. You can place a pillow under the ankle joint to promote venous blood return and alleviate swelling.

Consume more soda water to alkalize the urine and facilitate uric acid excretion.

In your daily life, please consider the following precautions for gout management:

Engage in moderate exercise to boost overall immunity and avoid exposure to cold and dampness, excessive fatigue, and stress.

Wear comfortable shoes to prevent joint injury.

Maintain a healthy weight, abstain from alcohol, and stay well-hydrated.

I hope this information is helpful. Please don't worry or be anxious; gout is a relatively common condition in clinical practice, and with proper treatment, symptoms can be alleviated. I apologize for the delay in my response due to the slower nature of text editing on a mobile device.

33.

Symptom Description: Severe osteoporosis, often experiencing hip joint dislocation when walking, sitting, or lying down in the wrong posture. Family members can assist in relocating the hip joint, but inflammation persists for a long time, causing significant pain during daily activities. It's challenging to perform tasks, and rest is required. Treatment at a clinic with anti-inflammatory IV drips is necessary for relief. Last year, a medical examination showed severe osteoporosis. I tried two types of calcium supplements from the pharmacy, but they didn't seem to be effective and even caused leg cramps.

Duration of Illness: 6 years

Hospital Examinations: Osteoporosis diagnosed during a medical examination

Medication: Alternating between two types of calcium supplements

**The doctor's answer on the website**

Generally, the diagnosis of osteoporosis is made through bone density testing, primarily based on the T-score indicated on the test results. A T-score between -1.0 and -2.5 suggests low bone mass, while a T-score below -2.5 indicates osteoporosis. It would be helpful to provide her bone density test results for a more accurate assessment of her condition.

If osteoporosis is diagnosed through testing, simply increasing calcium intake through diet is not sufficient. Treatment typically involves medications in addition to basic calcium and vitamin D supplementation.

However, calcium supplementation alone is insufficient. It needs to be complemented with vitamin D to enhance calcium absorption, and there is also a need for specific medications to treat osteoporosis. There are two main approaches to treatment.

Option 1: Prevent further bone loss and gradually increase bone mass. Choice 1: Oral alendronate sodium (Fosamax) Specific regimen:

Basic treatment: Calcium carbonate with vitamin D3, with calcium intake not less than 1500mg/day and vitamin D not less than 800 IU/day, taken after meals.

Anti-resorptive medication: Alendronate sodium (Fosamax or equivalent) Explanation: Fosamax is priced at approximately $70 per tablet, taken once a week. The annual cost is approximately $3500. It is recommended for use over 3-6 years to combat osteoporosis.

Choice 2: Intravenous injection of zoledronic acid (Reclast)

Basic treatment: Calcium carbonate with vitamin D3, with calcium intake not less than 1500mg/day and vitamin D not less than 800 IU/day, taken after meals.

Anti-resorptive medication: Intravenous injection of zoledronic acid (Reclast) Explanation: Reclast is priced at approximately $3200 per infusion, administered once a year for 3-6 consecutive years.

Option 2: Promote the regeneration of bone tissue to increase bone mass more rapidly.

Basic treatment: Calcium carbonate with vitamin D3, with calcium intake not less than 1500mg/day and vitamin D not less than 800 IU/day, taken after meals.

Bone formation-promoting medication: Teriparatide (Forteo) Explanation: Subcutaneous injection of Teriparatide (Forteo) once daily, similar to insulin injection. It is recommended to inject after dinner. The cost is approximately $5810 per pen, which can be used for 28 days. The treatment course is 1-2 years. Currently, there is a charity program available: Buy 6 pens, get 7 pens (a total of 13 pens, sufficient for 1 year), with a total cost of approximately $34,860 for a year. After using Teriparatide for 1-2 years, it is recommended to continue with Option 1 to maintain increased bone mass and prevent bone loss after discontinuation of treatment.

34.

Hello, I am 27 years old, 184 cm tall, and weigh 163 kg. I love playing basketball. Two years ago, I used to experience knee pain and discomfort at night after playing basketball every day. An MRI examination revealed synovitis. Nowadays, I occasionally experience knee pain after playing basketball. At night, I often feel uncomfortable with the positioning of my feet, and bending my legs produces a crisp sound that makes me feel somewhat relieved. The loudest sound occurs when I bend my legs in the morning. What could be the cause of this situation?

**The doctor's answer on the website**

Based on the information you provided, your current knee issue could be related to synovitis, as diagnosed through an MRI examination. However, since the problem has persisted for a long time without significant improvement, there may be other underlying factors contributing to your symptoms. Therefore, I recommend seeking further evaluation from a reputable orthopedic specialist at a good hospital.

Here are some steps you can consider:

Consult with a specialist who can examine your knee to determine the exact cause of the discomfort and the clicking sound you've described. They may perform additional tests or imaging, such as X-rays or CT scans, to assess the bone structure more thoroughly.

It's important to temporarily refrain from playing basketball or engaging in activities that exacerbate your symptoms. Reduce walking, squatting, or stair climbing in your daily life to minimize stress on the knee joint.

Use a knee brace or support during walking to protect your knee, regardless of the underlying cause.

Ensure your knee is kept warm, especially in cold conditions. Avoid direct exposure to cold air conditioning or fans. Consider applying warm compresses to the knee area at home.

If you experience significant discomfort or pain, you can consider over-the-counter pain relievers, such as ibuprofen, but consult with a healthcare professional first, especially if you have any allergies or underlying health conditions.

Please note that this advice is for general guidance, and it's essential to consult with a healthcare provider for a proper diagnosis and personalized treatment plan. Whether it's synovitis or another issue, with the right treatment and care, you should be able to recover and return to normal activities over time.

35.

Hello, doctor. My knees hurt significantly during rainy seasons and seasonal changes. It feels like the pain originates from inside the bones and can affect the nerves in my buttocks. I need to use heating pads for relief. Is this symptom indicative of gout? Translated into English.

**The doctor's answer on the website**

Hello, doctor. I apologize for keeping you waiting. Based on the information you provided, it doesn't seem to resemble gout. Firstly, you are a young woman, and gout is less common in this age group, with young men being more prone to it. Secondly, gout attacks are typically triggered by dietary factors, such as alcohol consumption, seafood, hotpot, red meat, organ meats, etc., leading to elevated blood uric acid levels. Your symptoms, however, occur only during rainy seasons or seasonal changes, which is not in line with the typical triggers of gout.

From your current symptom description, it appears to be localized knee joint pain, and you mentioned that it feels like bone pain and can radiate to your buttocks. Therefore, considering your symptoms, it is more likely related to other factors. It could potentially be synovitis of the knee joint, especially if you spend a lot of time sitting at a desk, leading a sedentary lifestyle without much physical activity. Exposure to cold conditions could exacerbate such local symptoms.

Another possibility to consider is whether you have issues with lumbar disc herniation or vascular problems. Exposure to cold conditions can stimulate blood vessels and nerves, leading to similar symptoms. If you have been experiencing these symptoms for a prolonged period, it would be advisable to consult a hospital and see an orthopedic specialist. A comprehensive examination by the doctor can help determine the cause of your knee pain and radiating discomfort in the buttocks.

They may recommend further diagnostic tests such as knee joint MRI, lumbar CT scans, electromyography (EMG) for lower limbs, and vascular ultrasound. Additionally, blood tests for uric acid, rheumatoid factors, and calcium levels might be conducted. Once a definitive diagnosis is made, appropriate treatment measures can be taken to address the issue.

However, if you are unable to access medical care immediately or lack the necessary resources, you can follow some home care strategies. Resting adequately is essential. Engage in light activities like walking, but avoid strenuous exercises that could worsen knee pain. Consider using a knee brace when walking to provide support.

Moreover, ensure you stay warm, especially during seasonal changes or rainy weather. Wearing an additional layer of clothing or using a knee brace with insulation can be helpful. If you continue to experience symptoms, over-the-counter medications such as ibuprofen, glucosamine, and chondroitin may provide relief. A combination of these treatments can help alleviate your symptoms.

In summary, while I cannot provide a definitive diagnosis, I recommend seeking medical attention to get a proper evaluation and diagnosis. In the meantime, following the home care suggestions mentioned above should help manage your symptoms and potentially provide relief. Rest assured that with the right diagnosis and treatment, your condition can improve, and you should not worry excessively.

36.

Hello, I understand your concerns about the elderly family member's lower back and leg pain. Given their age of 73 and a history of mild hypertension, it's essential to approach their health with care.

The reported symptoms of experiencing lower back and thigh pain after walking a few hundred meters may be due to various factors, including musculoskeletal issues or vascular problems. Since there is no disc herniation observed in medical examinations, the cause of the discomfort may be related to other factors, such as muscle strain, joint issues, or circulation problems.

Given the uncertainty regarding the effectiveness of over-the-counter health supplements like "舒筋健腰丸" (Shu Jin Jian Yao Wan), it is advisable to prioritize medical advice and evaluation over self-prescribed supplements. While such supplements may have their place in traditional medicine, it's crucial to ensure that they are safe and effective, especially for an older individual with underlying health conditions.

**The doctor's answer on the website**

Hello, thank you for your patience. Based on the information provided, I would consider the possibility of an old compression fracture and osteoporosis. Further evaluation is needed to determine if there is a herniated lumbar disc.

Analysis:

The patient is 73 years old, an age at which bone loss accelerates, increasing the risk of osteoporosis. Osteoporosis is a risk factor for fractures, particularly in the wrist, hip, and thoracolumbar spine. Osteoporosis typically doesn't present with symptoms, but when it does, it can cause generalized aches, back pain, and more.

Considering the patient's symptoms of back pain and thigh soreness, it aligns with the symptoms of osteoporosis. Additionally, the presence of symptoms in both the lower back and thigh raises the need to rule out a herniated lumbar disc.

The X-ray provided is somewhat dated, and while it shows a lumbar vertebra that seems to have experienced a compression fracture, it may not reflect the current situation. Such fractures are often associated with osteoporosis and can contribute to back pain. However, the X-ray may not show whether there is a herniated lumbar disc.

Therefore, considering the above analysis, I suggest the following:

Rest: Prioritize rest for now, avoiding heavy lifting.

Lumbar Support: Consider using a lumbar support device for 1-2 weeks, but avoid prolonged use, as it can potentially worsen the injury.

Lumbar MRI: It's advisable to undergo a lumbar spine MRI to assess the extent of the compression fracture, rule out nerve compression, and evaluate the spinal discs.

Medication: Depending on the MRI results and diagnosis, consider appropriate pain relief and medications for osteoporosis.

Bone Density Scan: A bone density scan (DEXA scan) can help determine the severity of osteoporosis.

In summary, considering the likelihood of an old compression fracture and osteoporosis, it's essential to pursue a comprehensive evaluation and treatment plan. Osteoporosis is a common condition in older adults, and with proper care, it can be managed effectively. While "舒筋健腰丸" (Shu Jin Jian Yao Wan) doesn't have clear scientific evidence or medical experience to support its use, I would recommend following the guidance provided above and seeking medical advice for a tailored treatment plan. Please be assured that proactive treatment can yield favorable results.

37.

Hello, doctor. I have a hard lump on the back of my hand that feels like it's on the bone. When I press it, it hurts. It has been there for about two weeks. I noticed it the day after I spent a long time carrying my child. The lump doesn't move, and I don't typically feel any pain unless I press on it. Duration of the condition: Two weeks.

**The doctor's answer on the website**

Hello, and thank you for your trust. Based on the image you provided, it is likely a tenosynovial cyst. A tenosynovial cyst is a cystic swelling that occurs within the tendon sheath of a joint, typically caused by degeneration of connective tissues within the joint capsule, ligaments, or tendon sheath. The cyst contains colorless or slightly colored thick, viscous synovial fluid, and its wall is composed of dense, tough fibrous connective tissue. Tenosynovial cysts are commonly found on the dorsal aspect of the wrist and foot. They often affect young and middle-aged individuals, with a higher prevalence in females. The onset is gradual, and a round lump may be observed at the affected site. There can be mild discomfort or pain, and in severe cases, it may cause functional impairment.

As for the treatment of this condition, some tenosynovial cysts may regress on their own, but others may require various treatment methods. In your current situation, you can try applying pressure to the cyst to rupture it. However, there is a possibility that the cyst wall remains intact, leading to a recurrence. The definitive treatment is surgical excision. Given the current small size of the cyst and if it doesn't significantly impact your daily life, you can choose to observe it for the time being.

38.

Hello Dr. He, there is a soft lump on the back of my foot that has been there for a few years. It's not painful when lightly pressed, but there is a slight discomfort when pressed firmly. Recently, it has grown in size, and I haven't used any medication. It doesn't affect my daily life. I wanted to ask if this could be a form of tendon sheath inflammation?

**The doctor's answer on the website**

If you can feel a lump and experience some discomfort when pressing on it, the most common cause in this location is indeed a tendon sheath cyst. To confirm the diagnosis, you should have an ultrasound done.

The main cause of a tendon sheath cyst is localized tenosynovitis, where the body attempts to encapsulate it, resulting in the formation of a fluid-filled sac, which is the cyst itself.

For localized tenosynovitis or tendon sheath cysts with discomfort, you can apply topical Voltaren gel and take Celebrex capsules orally. If possible, you can also consider physical therapy at a local hospital's rehabilitation department, which can help alleviate pain and discomfort. If these treatments don't work and the cyst continues to grow, you may opt for a local anesthesia surgical removal, which is a minor procedure and can be done on an outpatient basis.

If your symptoms are not severe at the moment, you can leave it alone. Do not attempt to drain it, as the cyst's membrane responsible for secreting synovial fluid will remain, and the cyst may quickly return. Tendon sheath cysts have the potential to resolve on their own.

In daily life, due to pressure or friction, they may rupture and disappear, but the cyst wall remains, with a 50% chance of recurrence, so it's not advisable to attempt to rupture it.

Based on your description, it is likely a tendon sheath cyst, and I recommend you get an ultrasound for a confirmed diagnosis.

39.

Congenital right hip dislocation for 15 years, and no treatment has been administered during this time!

Additional information:

Apart from limping, there are no other notable symptoms.

There is no pain upon pressing.

There is a sensation of pain after strenuous activity.

Previous examination revealed the diagnosis of congenital right hip dislocation. I am seeking your opinion on whether it is necessary to undergo corrective osteotomy surgery. Can this surgery correct the condition and allow for normal gait and walking afterward?

**The doctor's answer on the website**

Hello, let's analyze the patient's condition below:

Based on the X-rays, the hip joint is completely dislocated, resulting in limping while walking. Currently, it is considered developmental dysplasia of the hip.

Recommendation: The natural course of untreated developmental dysplasia of the hip depends on age and the severity of the condition. A dislocated hip joint may function well for several years. However, over time, there may be a gradual progression of functional disability, pain, and accelerated development of osteoarthritis. The risks of these complications are not yet clear but may be related to the formation of a false acetabulum. For hip joint dislocations in children aged ≥18 months, the benefits of reduction should be weighed against the natural course of untreated hip dislocation. Major surgical risks include bone necrosis and proximal femoral growth disturbance, residual developmental dysplasia, and the need for additional surgical treatment. The benefits of successful surgery include correcting leg length inequality and preventing or delaying the onset of hip joint arthritis. The earlier the reduction is performed, the more time there is for acetabular development and remodeling, increasing the likelihood that the benefits of reduction surgery outweigh the risks. In general, unilateral dislocations can be reduced before the child reaches the age of 9 or 10, and bilateral dislocations can be reduced before the child is about 8 years old. If surgery is performed after the age of 9 or 10 (for unilateral dislocations) or after the age of 8 (for bilateral dislocations), the outcome is likely to be worse than not receiving treatment. Older dislocations typically already have femoral head deformities, which reduce the chances of successful concentric reduction. Furthermore, the incidence of bone necrosis after open reduction is much higher in older individuals. All things considered, it is advisable to undergo corrective osteotomy surgery, and typically, normal gait can be restored after the procedure. However, there is also a higher surgical risk associated with it.

40.

Hello doctor, I am a 36-year-old female. In the past six months, I haven't been working and have been spending a lot of time on my phone. Recently, I've been experiencing a sensation like something is pulsating on the left side of my head near my ear. This has been going on for a while, and sometimes it feels like it's flowing. Lately, I've been using air conditioning at night, and in the past couple of days, my fingers have felt slightly stiff when bending. I've also been experiencing back pain when waking up in the morning. My sleep quality is still good, but I still feel some pulsating sensations in my head, and the area feels a bit numb, as does a small part of my face. I had a head CT scan in December last year, and it was normal. Now, I also feel a slight coolness in my toes and fingers. Doctor, can you please tell me what these symptoms might be?

Additional information: Doctor, it's just a slight sensation in all my fingers, not severe, like a feeling of slightly reduced blood flow. The pulsating sensation is like the feeling of blood vessels pulsating. It was stronger a couple of days ago, but today it's just a little bit. Around the area where it's pulsating, there's a slight numbness, and there's a slight tingling sensation around my face, like a subtle static feeling. I don't have nausea or vomiting. When I lower my head, there's a soreness in the lower part of my neck, and everything else is the same. I haven't been taking any medication recently, except for antibiotics for gynecological inflammation.

**The doctor's answer on the website**

Hello, based on the information you have provided, there are several possible factors to consider regarding the symptoms you mentioned:

Regarding the pulsating sensation in your face, there are two potential factors to consider. First, it could be related to a neurological issue as facial nerves have a wide distribution, and this kind of discomfort is relatively common. However, neurological problems more commonly lead to pain rather than pulsating sensations. Nonetheless, it cannot be ruled out as a possible cause, and local muscle tremors might also contribute to the sensation of pulsation.

Another possibility could be vascular in nature, as you mentioned feeling a pulsating sensation. There might be some connection between the localized pulsation discomfort and blood flow, although this is relatively uncommon.

Regardless of the cause, many of these discomfort symptoms in the facial area may also be related to factors such as inadequate rest, fatigue, or exposure to cold stimuli. Therefore, for the time being, it may be worthwhile to address these possible triggers. You can consult a neurologist for this issue, given its connection to neurological symptoms.

As for the stiffness and coolness you feel in your fingers and toes, several factors could be responsible. First, it could be related to non-infectious inflammation of local soft tissues, which is often associated with increased activity or exposure to cold. This kind of inflammation, particularly around joints and synovial tissues, can lead to stiffness and discomfort.

The sensation of coolness might also be related to blood vessels. In response to cold stimuli, blood vessels in the peripheral regions like fingers and toes tend to constrict, which can result in slightly reduced blood supply and the sensation of coolness and stiffness.

The back pain in the neck area, especially due to prolonged bending of the neck, could lead to changes in the physiological curvature of the neck, making it more prone to discomfort and soreness, particularly when combined with exposure to cold. In most cases, this is unlikely to lead to severe issues and is primarily related to soft tissues.

In terms of treatment, it's essential to improve your lifestyle first. Avoiding potential triggers, such as prolonged neck bending and exposure to cold, is important. Keep the neck, hands, and feet warm, and consider applying heat, such as soaking your hands or feet in warm water, which can help with symptoms. Heat application can also promote local blood vessel dilation and improve blood supply, which can alleviate stiffness and discomfort.

Medication can also be considered for treatment. Non-steroidal anti-inflammatory drugs (NSAIDs) like sodium diclofenac can be used to address non-infectious inflammation in soft tissues, including muscle fascia inflammation in the back and synovial inflammation in the hands. These medications are generally taken after meals, and if you don't have a history of gastrointestinal issues, they are usually well-tolerated and effective in relieving pain and discomfort.

For the pulsating sensation in your face, especially with the numbness you mentioned, you can also consider supplementing with vitamins that support nerve health, such as methylcobalamin (vitamin B12). If the pulsating sensation persists, it's advisable to consult a neurologist. However, if your symptoms continue to improve with enhanced rest, local warmth, and medication, there may not be a significant underlying issue for now.

41.

The inner side of my thigh feels sore, especially when sitting down. I feel like there might be an issue with the joint in my buttocks.

**The doctor's answer on the website**

Based on the information you've provided, I believe your current issue may be related to synovitis in the hip joint. However, other causes cannot be entirely ruled out, such as muscle injury or arthritis. Given your relatively young age, if you've been involved in strenuous physical activity, muscle injury is more likely. If you haven't engaged in vigorous exercise or physical labor, the possibility of synovitis is higher. I'm not sure if there were any specific reasons or triggers before the onset of symptoms, so if you could provide more information, it would help in my assessment. If these symptoms have only appeared recently in the past one or two days, then I don't think there should be a significant problem, and the treatment should be effective. However, if the symptoms have persisted for several months, I would recommend you visit a hospital, schedule an appointment with an orthopedic specialist, and have them reevaluate the affected area. Additionally, they may recommend imaging studies, such as X-rays or an MRI, for a clearer diagnosis. If it is confirmed to be synovitis with effusion, there's no need to worry. Following the advice below should help:

Rest is crucial. Avoid strenuous physical activities, running, playing sports, or excessive walking for a while. Adequate rest is essential.

You can apply local heat therapy to the uncomfortable area on your own, or if possible, seek physical therapy at a hospital, such as using infrared therapy lamps.

Consider medication treatment. Apply topical ibuprofen gel to the painful area and take oral medications like ibuprofen and acetaminophen. These medications, when used together, should help with your recovery. I'll provide you with a prescription shortly, and you should be able to find these medications at a pharmacy easily.

As long as you actively follow these recommendations, I believe your symptoms should gradually improve. Please don't worry too much.

42.

I was diagnosed with synovitis in November last year and prescribed Bixinsijia capsules and Nimesulide tablets. I might not have rested enough, and I didn't see much improvement. Later, two swollen areas appeared on top of my knee, especially when I straighten my leg, they feel like swollen muscles. Recently, I used a synovitis granule preparation and applied a plaster, but there hasn't been significant improvement. However, I've been resting for nearly two months now, only taking a few hundred steps a day. I would like to ask if these swollen areas are related to synovitis, and my leg cannot fully straighten. When I do try to straighten it, my knee doesn't feel comfortable either.

**The doctor's answer on the website**

Hello, your current condition, given your age, appears to be related to issues in the knee joint. The symptoms you've described could be due to two main reasons. Firstly, it could be a result of synovitis, and secondly, it might be associated with local tendon and fascia inflammation. The swollen area you mentioned corresponds to the attachment site of a tendon near the joint, which can be affected by both synovitis and local tendon/fascia inflammation. Synovitis is the more likely cause. The treatment you are currently using is appropriate.

In general, I would recommend resting, reducing physical activity, keeping the affected area warm, applying local heat therapy, receiving physiotherapy and massage, and taking oral medications to improve blood circulation, reduce swelling, and alleviate pain. Additionally, you can consider using topical soft tissue ointments like Futhelin. For oral medications, you can try pain relievers and anti-inflammatory drugs like Xilebao. Amino-sugar and cartilage nutrition supplements can also aid in the treatment.

43.

Hello, about half a year ago, I injured my right ankle and it started to hurt. I didn't get an X-ray at the time. After using Yunnan Baiyao, it gradually improved. However, recently, after a night of heavy drinking, the pain has returned, and I can't put weight on my foot. I haven't had any recent injuries, and there are no visible abnormalities, just some warmth. What could be the issue?

**The doctor's answer on the website**

Hello, based on your description, there is a significant possibility of gout. Gout is a condition caused by elevated levels of uric acid in the blood, leading to the crystallization of uric acid in joints, resulting in painful inflammatory attacks in and around the affected joints. Alcohol consumption is a common trigger for gout.

Gout patients often experience sudden and severe pain in one or more joints, usually occurring at night. Other symptoms may include redness, swelling, increased skin temperature, and a shiny appearance on the affected joints.

Given your situation, it is advisable to visit a hospital for a uric acid test to confirm the diagnosis. If the pain is significant, you can consider taking non-steroidal anti-inflammatory drugs (NSAIDs) such as celecoxib or sodium diclofenac orally, and applying topical diclofenac gel. Stay well-hydrated and urinate regularly.

Daily Lifestyle Management: Diet:

Maintain adequate hydration and avoid alcohol, sugary beverages, and high-purine foods such as organ meats, pork, beef, lamb, shellfish, sardines, and tuna.

Limit alcohol consumption, especially beer.

Reduce the intake of high-purine foods.

Cut back on fructose-containing beverages.

Drink plenty of water, aiming for more than 2000 ml daily.

Increase the consumption of fresh vegetables.

44.

Hello, doctor. I'm 29 years old and weigh 120 kilograms. I gave birth in December last year, and initially, I wasn't very active. My heels didn't hurt, but around the time of the Lunar New Year, I was doing household chores like cleaning and mopping, taking care of my child, and even took a bath. I got tired, and it may have been because the shoes I was wearing while doing these activities had become stiff. As a result, I now feel discomfort and a bit of pain in both of my heels. My calves also have a sensation of tiredness, and it feels like there's poor blood circulation. When I lie down at night, my legs and heels feel tired, and it's uncomfortable. Soaking my feet doesn't seem to help. What should I do? Do I need to take any medication? I want to get better quickly and not leave any postpartum issues. Thank you for your help. I have attached a picture of the area on my heel that's uncomfortable. I have been applying topical analgesic cream, but the effect is not significant.

Additional information: I am not breastfeeding currently, both of my feet are in pain. When I lie flat, my heels don't hurt, but my heels and calves feel tired, and I have an uncomfortable sensation. After walking on the ground, my heels start to ache, with a slight numbness, and it feels like there's poor blood circulation, but it's hard to describe in detail.

**The doctor's answer on the website**

Hello, I have received your response. Thank you for waiting. (1) Based on your description, it is possible that you have plantar fasciitis. (2) The most common symptom of plantar fasciitis is pain and discomfort in the bottom of the foot. Tender points are often located on the sole of the foot, and sometimes the tenderness can be quite severe and persistent. The pain associated with it is often described as throbbing, burning, and stabbing. (3) In terms of treatment, it is essential to rest and use physical therapy to improve heel pain. This includes local massages, foot soaks, and wearing comfortable shoes with soft soles. Additionally, oral medication can be considered, such as taking one tablet of celecoxib per dose, once a day. If there is no significant improvement, local injections may be considered. (4) Recovery from the condition takes some time, so there is no need to rush. Generally, with the above treatments, most people recover without major concerns.

45.

Symptom Description: The sole of my right foot hurts. Lately, with the cold weather, when I walk outside, the sole of my foot hurts to the point where it's starting to affect my walking. However, it doesn't seem to hurt when I'm at home. In the past, the same area on the sole of my foot would hurt if I walked for a long time, but it would eventually recover on its own. This time, the foot pain persists for a while even after coming home from walking.

Duration of the Condition: It started recently with the cold weather when walking outside.

Hospital Examination: None.

Medication: None.

**The doctor's answer on the website**

Hello, based on your description, it is possible that you have plantar fasciitis. Plantar fasciitis is characterized by inflammation of the tendons or fascia in the sole of the foot. The most common symptom is pain and discomfort in the heel, with tenderness often near the heel on the sole of the foot. Sometimes, the tenderness can be quite severe and persistent. For now, you can consider conservative treatments:

Rest and immobilization, along with icing the heel for 10 to 15 minutes if there is no significant improvement.

Use arch supports or orthotic insoles with arch support to evenly distribute pressure on the sole of the foot, reducing the strain on the plantar fascia during weight-bearing activities.

Oral non-steroidal anti-inflammatory drugs (NSAIDs) for pain and inflammation relief. Topical creams or patches can also help stimulate nerve endings, reducing irritation and relieving pain.

Regularly soaking your feet in warm water can provide relief from the discomfort.

If your symptoms persist after a month, consider seeking medical attention, which may include a foot MRI and, if necessary, localized treatments.

46.

Hello, Dr. Zhou, I'm inquiring on behalf of my mother, who is 52 years old. Below is a description of her condition, and I would appreciate your assistance. Thank you.

Symptom Description: She experiences some pain in her left knee when going downstairs, and it's slightly milder when going upstairs. There hasn't been any recent injury, and the pain is localized to the left knee.

Duration of the Condition: It has been about a dozen days.

Medical Examination: She visited a local township hospital, where the doctor suggested it might be arthritis, but no additional diagnostic tests were conducted.

Medication: She took medication for two days, but there hasn't been a significant improvement, and she is currently not taking any medication.

**The doctor's answer on the website**

According to your description, your mother is currently 52 years old and has been experiencing left knee pain and discomfort for the past dozen days. The pain is particularly noticeable when going downstairs, with no recent history of injury. She visited a local township hospital where the doctor considered arthritis and prescribed medication for two days, but there hasn't been significant relief.

Considering your mother's age and the symptoms described, there is a high likelihood that she may be suffering from left knee osteoarthritis (degenerative joint disease), which is characterized by aging and degeneration of the knee joint, including the erosion of joint cartilage. Additionally, it may be associated with meniscus damage and ligament laxity, potentially leading to a vicious cycle of joint degeneration.

For further evaluation, I recommend getting an X-ray of the left knee joint and a knee joint MRI to clarify the specific damage and degeneration of the knee joint.

In terms of treatment, the focus should primarily be on joint care. In the short term, it's advisable for your mother to reduce walking and engage in minimal physical activity. When walking, she should wear a knee brace and avoid activities like climbing stairs or hills.

If the symptoms are severe, she can consider taking oral non-steroidal anti-inflammatory drugs (e.g., Celebrex, one tablet per day after a meal) for pain relief. Additionally, she can consider taking nutritional supplements for joint health, such as glucosamine sulfate.

However, I strongly recommend scheduling the MRI as soon as possible to assess the specific condition of the knee joint.

47.

Before the lockdown, I went to Huashan Hospital for sports rehabilitation due to left hip joint pain. I play badminton, and I'm 188 cm tall and weigh 194 pounds, primarily using my left leg to support my weight. Initially, I experienced soreness and pain when sitting for extended periods, and it was challenging to stand up. Later on, I began to experience radiating pain in my lower leg, making it difficult to cross my left leg. The doctor initially prescribed pain relief medication, followed by an MRI and rehabilitation sessions (once a week for five sessions). However, the MRI was not conducted due to the lockdown. Throughout the pandemic, I have been taking pain relievers, specifically etoricoxib tablets, but the pain has been worsening. Could you please provide some home rehabilitation exercises that I can do to alleviate the pain? I have some equipment at home, including resistance bands.

**The doctor's answer on the website**

Your current symptoms suggest hip joint arthritis likely caused by overexertion during physical activities, and it appears that oral medication has not provided significant pain relief.

Here are some recommendations:

Avoid high-impact activities like running and jumping in the near term. Also, minimize long-distance walking.

Apply local heat therapy to the painful area. You can use hot showers during bathing for this purpose.

When sleeping, avoid putting pressure on the painful area.

Pay attention to your diet, and avoid spicy, irritating foods, as well as excessive sugar consumption. It's crucial to avoid smoking and alcohol, particularly during this period.

Considering your current pain, which likely involves hip joint arthritis changes, it's not advisable to engage in specific rehabilitation exercises. Instead, focus on short-distance, slow-paced walking exercises. Avoid prolonged sitting or lying down without movement, as this can accelerate degenerative inflammatory changes. Therefore, it's important to engage in some activity, and try to limit each walking session to 5-10 minutes.

48.

Symptom Description: Hello doctor! My father is 65 years old this year. Since November of last year, he has been experiencing pain on the inside of his right knee joint. At that time, the joint was swollen, and his lower leg was swollen. He could only walk straight ahead and couldn't move it left or right, as it would cause pain on the inside of the knee joint. It gets a bit better after resting for a while. Duration of Illness: It started in November of last year (2021). Hospital Examination: The doctor diagnosed it as osteoarthritis. Medication Situation: He has been taking medication for promoting blood circulation and reducing stasis, but it hasn't been effective.

**The doctor's answer on the website**

The information you provided has been carefully reviewed, and the knee joint osteoarthritis appears to be primarily age-related wear and tear. The medication mentioned may not be specifically targeting the condition.

The treatment for osteoarthritis mainly consists of three stages, referred to as stepwise treatment:

In the early stage, you can use topical patches and apply hot compresses (15 minutes with a towel heated to 40-50 degrees Celsius, 3-4 times a day) to relieve symptoms. Commonly used patches include ibuprofen gel patches.

If there is no history of gastric ulcers or gastrointestinal bleeding, you can also consider oral non-steroidal anti-inflammatory painkillers such as celecoxib or naproxen.

Additionally, it's important to reduce physical activity, prioritize rest, especially avoiding activities involving climbing stairs and heavy lifting.

As age increases, knee joint wear and symptoms may worsen. Considering the current symptoms, it is advisable to initially explore the conservative treatment options mentioned above. If there is no improvement, a follow-up visit to an orthopedic specialist at the hospital may be necessary, and further treatments like intra-articular sodium hyaluronate injections can be considered.

49

Symptom Description: After waking up from sleep, the left foot suddenly became red and swollen on the side, accompanied by intense and continuous pain that seems to pulse with each heartbeat. Walking or flexing the foot exacerbates the pain.

Medication Situation: Applied Voltaren gel, but it doesn't seem to provide significant relief, and the pain remains severe.

Duration of Illness: 1 day.

**The doctor's answer on the website**

Hello, your inquiry has been received, and I appreciate your trust. I had an emergency surgery last night, and I apologize for the delay.

Based on your description, experiencing sudden, significant, continuous pain in the left foot after sleeping, with exacerbation upon walking or flexing the foot, and considering the redness and swelling in the provided image, a preliminary consideration suggests gout as a possible cause. The symptoms align with those commonly associated with gout.

Recommendations:

Rest, elevate the affected limb, avoid excessive movement, and stay hydrated.

Treatment with oral anti-inflammatory and pain-relieving medication; a prescription will be provided accordingly.

Blood tests including a complete blood count, erythrocyte sedimentation rate, and uric acid level to pinpoint the specific cause.

If conditions allow, physical therapy options like medium-frequency pulse treatment can be considered.

I hope this information proves helpful to you.

50

Hello, doctor. I've been experiencing pain in the area below my left big toe for about a week now. The pain is primarily when I walk or apply pressure, and there don't seem to be any external abnormalities or noticeable swelling or hardness when I touch it. It feels the same as my other foot. Can you please advise on the possible cause?

**The doctor's answer on the website**

Thank you for your inquiry. Based on the symptoms you described, pain in the weight-bearing area below the big toe can be attributed to several common conditions:

The most common cause is plantar fasciitis, which can result from wearing ill-fitting shoes or excessive walking. If this is the case, I recommend resting more, reducing walking, switching to comfortable shoes with soft soles, soaking your feet in warm water at night, and applying topical pain relief creams like Voltaren. Typically, these measures can provide relief within 3-5 days.

Another possibility is gout, which can be related to dietary factors such as alcohol consumption or seafood intake. It would be advisable to check your uric acid levels. If uric acid levels are elevated, specific treatments to control uric acid and alleviate pain may be necessary. A diet with light and non-inflammatory foods, increased water intake, and avoidance of red meat, lamb, seafood, and beer may be recommended.

Thank you for your inquiry.

51.

Hello, doctor. My uric acid level is 644.7, and I've been diagnosed with gout. Can you please provide specific information about low-purine foods? I've looked online, and some sources say that chicken, duck, fish, and meat are okay to eat, while others say they're not. I'm also confused about which soy products are safe to consume and which are not. I hope to receive your guidance. Thank you!

**The doctor's answer on the website**

Hello, first of all, thank you for your trust. Based on your medical history, gout is a consideration. The main focus should be on dietary control and medication. Regarding dietary control:

Control protein intake to around 1g per kilogram of body weight per day. This means you should limit the intake of high-protein foods like chicken, duck, fish, meat, and legumes, considering your body weight.

Avoid high-purine foods, primarily animal organs and seafood.

Abstain from alcohol strictly, avoid triggering factors, encourage increased water intake to maintain a urine output of over 2000ml per day, and consider alkaline medication when the urine pH is below 6.0 and the urine urate concentration is above 1000nmol/L.

Medication treatment primarily focuses on lowering blood uric acid levels and managing acute gout attacks. Medications for lowering blood uric acid levels include allopurinol, while medications for managing acute attacks include colchicine.

Wishing you a speedy recovery.

52.

Basic Information: Female, 30 years old, not very physically active, doesn't wear high heels regularly, and her daily uric acid levels range from 350 to 400 umol/L. She has never had gout before.

Symptom Description: About half a month ago, without any prior injury, she started experiencing pain in her right heel. There is no redness or swelling. The pain typically occurs when getting up after prolonged sitting, especially when putting weight on the heel while landing or when the foot is planted firmly on the ground (even though the heel itself doesn't touch the ground during the latter). The pain is not present when sitting, except when intentionally applying pressure to the heel or rotating the ankle. In other positions, there is no pain. After walking for a while, the initial pain gradually subsides, and she can walk normally without pain. Additionally, the pain is continuous, not intermittent; it occurs when pressure is applied but not during relaxation.

Over the past half month, the pain has not improved and has even worsened. She has not sought medical attention yet. I would appreciate your assistance. Thank you.

**The doctor's answer on the website**

I have carefully reviewed the information you provided, and based on your symptoms, there is a significant possibility of plantar fasciitis.

Plantar fasciitis is often related to several factors, with the fundamental cause being low arches (flat feet). Accumulated micro-injuries from daily activities can contribute to the gradual onset of symptoms, primarily characterized by pain in the sole and heel of the foot. The pain is usually most noticeable when first putting weight on the foot after rest, and it tends to lessen with activity but can worsen again with prolonged activity.

Considering your current situation, here are some recommendations for your reference:

Try to wear shoes with softer and flatter soles as much as possible. Avoid excessive and prolonged walking.

You can purchase a fascia ball online (similar to a tennis ball) for stretching and massaging the plantar fascia. The method is straightforward: sit down, place your bare foot on the ball, and roll it back and forth under your foot. Do this with a level of pressure that is tolerable but induces a slight discomfort. Repeat this exercise two to three times a day for at least five minutes each time.

You can also use a towel for foot exercises. Roll up the towel with your toes and then relax them repeatedly. Repeat this exercise with the same frequency as mentioned in the previous point.

If the symptoms persist or worsen, you may consider using pain-relieving medications, such as topical analgesics. Alternatively, you can seek treatment at a rehabilitation medicine department in a hospital, where physical therapy can be provided, which is likely to provide more effective relief from the pain.

53.

I am a 37-year-old male who enjoys running regularly. Recently, I've been experiencing mild pain on the top of my right foot, somewhere between the base of my big toe and the middle of my ankle, although I can't pinpoint the exact location. When I press on the area, there is no pain. The discomfort is more pronounced after sitting for extended periods or upon waking up, and it tends to ease after walking for a while. It has been going on for over ten days now. I'm not sure if I should be concerned. Thank you.

**The doctor's answer on the website**

Hello, based on your description, there is a possibility of plantar fasciitis, which is a common cause of heel pain. Plantar fasciitis is an aseptic inflammation at the attachment of the plantar fascia to the medial tubercle of the calcaneus, and it often results from repeated overstretching of the plantar fascia due to prolonged walking or running, leading to localized muscle and fascial strain (repeated microtrauma) and causing aseptic inflammation and pain in the plantar and heel area.

Causes: The primary cause is the repetitive overloading of the plantar fascia and its attachment points, and secondary causes may include structural or biomechanical abnormalities of the foot arch, leading to repeated microtrauma at the attachment point of the plantar fascia. Your weight could also be a contributing factor.

Clinical Presentation: The hallmark symptom of chronic plantar fasciitis is sharp pain on the inside front of the heel. The pain is most noticeable during the first few steps after getting out of bed in the morning but tends to ease as the plantar fascia becomes more relaxed with walking. However, excessive walking or stretching of the plantar fascia can aggravate the symptoms. Gentle massage and physical therapy can alleviate pain temporarily.

54.

Hello, doctor. Can you please take a look at a small lump that has developed on my wrist? It feels hard when touched, like a small grain of rice, but it moves when I move my hand or make a fist. It's usually not painful or itchy, but after I squeezed it a few days ago, it has been a bit painful recently and slightly tender to touch. It also seems to have grown slightly compared to before. Is this normal?

**The doctor's answer on the website**

Hello, thank you for your trust. Based on the image you provided, a tenosynovial cyst is a likely possibility. A tenosynovial cyst is a cystic swelling that occurs within the tendon sheath of a joint and is typically caused by degeneration of connective tissue within the joint capsule, ligaments, or tendon sheath. The cyst contains thick, colorless or orange, pale yellow viscous fluid, and its wall consists of dense, tough fibrous tissue. These cysts are often single-chambered and frequently occur on the dorsal side of the wrist and the top of the foot. They are more common in young adults and are more frequently seen in females. The onset is gradual, and a round lump can be observed at the affected site, accompanied by slight tenderness. In severe cases, it can cause some functional impairment for the patient.

In terms of treatment, some tenosynovial cysts may resolve on their own, but others may require intervention. For your current situation, you mentioned that you squeezed it and it ruptured, but since the cyst wall remains intact, there is a possibility of recurrence. The definitive treatment for this condition is surgical excision. Given the small size of the cyst and if it does not significantly impact your daily life, you may choose to observe it for now.

55.

Symptom description: A 34-year-old male has developed a red bump at the joint of the left thumb. Pressing on it causes a sore sensation, and there are occasional spontaneous sharp pains.

Duration of the condition: Over two months. Initially, it was not a concern, but it has been growing in size.

Hospital examination: None.

Medication use: None.

**The doctor's answer on the website**

Hello, in your current situation, it is likely a tenosynovial cyst that has developed at the joint of your left thumb. This is a cystic swelling that often occurs near joints and can result from prolonged friction or improper use of the hand. I recommend visiting a hospital for a local ultrasound examination to confirm the diagnosis.

Regarding this condition, I suggest consulting a general surgery department at the hospital. Tenosynovial cysts primarily contain liquid, so they can be treated through aspiration to remove the cyst fluid or through surgical excision. There is no need to be overly concerned as it is a manageable issue.

Since tenosynovial cysts tend to recur, it's important to take precautions in your daily life. Get adequate rest, maintain good nutrition, keep the affected area warm, and minimize unnecessary friction at the joint. You can also perform gentle massage in the affected area.

56.

This morning, I noticed a raised, reddish lump near the base of my right big toe. It doesn't feel like bone, and it seems somewhat soft, without any pain when pressed. It appeared red in the morning but was less red in the evening, though it's still present. I'm not sure what it could be. Thank you. I am in good health and don't have any chronic illnesses.

**The doctor's answer on the website**

It is highly likely that you are dealing with a tenosynovial cyst at the moment.

A tenosynovial cyst is essentially a cyst that forms in the tendon sheath, and it can occur either inside a joint or within the tendon sheath itself. While the exact cause is not clear, some researchers have suggested that tenosynovial cysts represent a mucoid degeneration of structures surrounding the joint.

The most common location for tenosynovial cysts to develop in the body is on the dorsal side of the wrist and the top of the foot, accounting for about 70% of cases. In reality, many patients visit outpatient clinics with the discovery of these lumps in these areas.

To put it in simpler terms, it's like having a balloon (the cyst wall) in these areas filled with a pouch of fluid (the cyst's fluid).

Tenosynovial cysts are generally considered a benign condition.

To confirm the diagnosis, it's advisable to consult with an orthopedic specialist and undergo an ultrasound examination.

57.

Symptom Description: There is a small blister on the hand that is neither painful nor itchy. When the hand is flat, it is not very noticeable, but when pressure is applied on the wrist, it becomes more pronounced. Occasionally, there is a mild pulling or discomfort sensation in the joint, especially when lifting heavy objects. The individual has a job that involves prolonged typing on a computer keyboard and also has a habit of using a smartphone.

Duration of the Condition: The symptoms were only noticed today.

**The doctor's answer on the website**

Based on your current job nature and lifestyle habits, there is a significant possibility of a wrist tenosynovial cyst, which is a localized inflammatory condition around the wrist tendons or nerves caused by long-term repetitive strain on the wrist joint.

Given your current symptoms, here are some treatment recommendations:

Since there is no significant pain or issues like numbness in the hand or limited finger mobility at the moment, I do not recommend oral medications for now. Instead, you can apply some topical ointments, which I will provide in a prescription, pending review. I can send it to you after it's approved.

In the short term, you can consider wearing a wrist brace. This can increase wrist joint stability and provide localized pressure, potentially reducing the enlargement of the cyst.

Try to minimize prolonged use of a mouse, keyboard, or smartphone in the near term. Take breaks every half hour or so to stop wrist movement and perform slow, controlled, full-range wrist exercises to relax the tension in the wrist tendons.

You can apply local warm compresses 2-3 times a night. This may help reduce the size of the cyst.

In the future, monitor any changes in the cyst. If it continues to grow, I recommend consulting an orthopedic specialist for ultrasound-guided aspiration, where the fluid inside the cyst can be drained.

58.

I would like to inquire about the possibility of treating a tenosynovial cyst during pregnancy. Would bursting it have any impact on the fetus? I underwent tenosynovial cyst removal surgery at the end of last year, but it recurred immediately after the stitches were removed. I haven't addressed it since becoming pregnant, but it has been growing larger. I experience pain when using my wrist or making certain movements, so I'm considering bursting it. However, I'm concerned about potential effects on the baby.

**The doctor's answer on the website**

Hello, thank you for your trust, and I've carefully reviewed your question.

You are currently in the midst of pregnancy and have concerns about treating a tenosynovial cyst. Here are a few considerations for dealing with a tenosynovial cyst during pregnancy:

If the cyst is asymptomatic, doesn't significantly impact your daily activities, or causes only mild discomfort, you may opt for observation without specific treatment.

Bursting the cyst using techniques such as aspiration or compression to allow the fluid to drain can be considered. These methods typically do not have adverse effects on the fetus.

Surgical treatment is an option, but it's important to note that tenosynovial cysts have a certain recurrence rate. Therefore, if surgical intervention is contemplated, it's advisable to consult a specialized department in a reputable hospital. Surgical procedures involve the use of anesthesia and related medications, which may have potential effects on the fetus. As such, this method is not currently recommended during pregnancy.

Tenosynovial cysts are benign in nature, so there's no need to overly worry. If the cyst is not causing significant issues, you can consider addressing it after giving birth.

Wishing you the best.

59.

Hello, doctor. I have a lump on my right wrist, and I went to the hospital for an examination. The ultrasound results indicated a tenosynovial cyst, with measurements of approximately 10mm x 6mm. It doesn't cause any pain or itching, and it appeared suddenly. What should I do next?

**The doctor's answer on the website**

Alright, based on your test results, the diagnosis of a tenosynovial cyst is confirmed. The exact cause of most tenosynovial cysts is unknown. These cystic structures often occur near or attach to (often with a stalk structure) tendon sheaths and joint capsules. The cyst wall is smooth, fibrous, and of varying thickness. The contents of the cyst are typically clear, gel-like, viscous, or sticky fluid, which may sometimes consist of hyaluronic acid.

Most tenosynovial cysts are solitary anomalies. Dorsal wrist tenosynovial cysts often arise from the radiocarpal joint and account for approximately 65% of wrist and hand tenosynovial cysts. Palmar wrist tenosynovial cysts originate from the distal radius, constituting about 20%-25%. Flexor tendon sheath cysts (originating from the dorsum of the distal interphalangeal joint) make up the remaining 10%-15%. Tenosynovial cysts can sometimes resolve spontaneously.

In most cases, tenosynovial cysts do not require treatment. However, if a patient is troubled by the appearance of the cyst, experiences pain, or has tenderness upon touch, further treatment may be considered. Treatment options include the following:

For relatively soft cysts, they can be gently pressed to rupture them. However, this method can lead to recurrence.

If pressing doesn't work or the cyst is not easily ruptured, aspiration of the cyst fluid can be performed, often accompanied by compression bandaging. This approach is effective in approximately 50% of patients.

For patients in whom non-surgical treatment is ineffective (40%-70%), surgical excision may be required. Excision can be performed using arthroscopy or standard open surgery. The recurrence rate after surgical excision is approximately 5%-15%.

In summary, there's no need to be overly concerned about tenosynovial cysts. They are like a water-filled lump inside the hand and do not have malignant potential. As long as there is no pain and it doesn't significantly affect your daily life, you can continue to observe it. If you find it aesthetically bothersome and wish to address it, you can consult with an orthopedic specialist for evaluation and consider the treatment options mentioned earlier, which may include surgery if other methods are ineffective.

60.

Symptoms and Duration of Illness: Six months

Medical Consultation and Medication: In August of last year, I experienced lower back pain and stiffness while playing basketball. When I returned home, the symptoms worsened to the point where I couldn't bend over. I couldn't even turn in bed and had to spend two days lying down before I could barely stand. Initially, I thought it was just a strained lower back muscle, so I didn't pay much attention. I rested for about two weeks and then resumed playing basketball. After playing basketball a few more times, I began to experience significant pain on the right side of my buttocks, which sometimes radiated down to my thigh and the outer side of my lower leg. There's also occasional pain and a burning sensation in the upper part of the outer ankle, numbness near the base of my little toe on the top of the foot, and pain on the outer side of my lower leg near the knee. Additionally, the area around the base of my big toe on the sole of my foot and my heel often feel achy and weak, with pain when pressed. Currently, I experience these symptoms almost daily, with varying intensity. I commute two hours by car for work every day. An MRI taken on April 5th showed disc protrusions between the 4th and 5th lumbar vertebrae and between the 5th lumbar and 1st sacral vertebrae.

Questions to Be Addressed:

Is surgery required for this condition? If not, what medications and exercises can help relieve the pain and strengthen the lower back muscles?

Are the symptoms of weakness and achiness in the sole of my foot related to the lumbar disc protrusions? How can these symptoms be relieved and treated?

**The doctor's answer on the website**

Considering the characteristics of your symptoms and the results of the lumbar MRI, the diagnosis is likely lumbar disc protrusion. Given your age, conservative treatment is recommended initially to improve your symptoms.

The achiness and weakness in the sole of your foot are also related to the compression of nerves caused by the lumbar disc protrusion. To address these symptoms, conservative treatment options should be considered:

Lifestyle adjustments are crucial: Avoid bending over, lifting heavy objects, and prolonged sitting (leaning forward while sitting exerts the most pressure on the lumbar discs and is most likely to cause protrusion). Try to lie down as much as possible during rest, but complete bed rest is not recommended. Short walks in daily life are not restricted. Keep your lower back warm, and you can use hot packs or take a hot shower for 5-10 minutes to relieve lumbar muscle tension. Consider wearing a lumbar support belt when driving for extended periods or during travel, such as by plane or train. It's not necessary for everyday use.

Medication treatment aims to improve acute symptoms: Oral anti-inflammatory and pain-relieving medications, as well as topical ointments, will be provided in the prescription.

Additionally, you can consider rehabilitation therapy with a physiotherapist: Modalities like low-frequency electrical stimulation, medium-frequency stimulation, laser therapy, and traction may be used for rehabilitation. It's recommended to have 10-15 sessions, 3-4 times a week. During the acute pain phase, daily therapy may be recommended.

Further evaluation and treatment: If conservative treatment for one month does not show improvement or if there is noticeable numbness, increasing pain in the lower limbs, muscle weakness, and a pronounced limp when walking, it is advisable to revisit an orthopedic specialist for a reassessment of the lumbar disc protrusion using MRI. Severe lumbar disc protrusions causing nerve compression may require surgical treatment.

Thank you.

61.

Hello doctor, when I press with my hand a few levels above the coccyx, it feels more painful, but I don't feel anything if I don't touch it. Additionally, in the lower back on both sides of the spine, there is pain when pressed, with one side being more pronounced than the other. After standing for one or two hours, my lower back feels very sore. This has been going on for about a year or two, and I haven't sought medical attention before. Recently, I had a massage and found it to be very painful. I would like to know what the possible condition could be, and if I were to get a medical examination, what type of CT or X-ray should I have?

Additional information:

I work as an online customer service representative for about ten hours a day.

I haven't had any injuries, but in the past, when my lower back was uncomfortable, I would twist left and right while lying in bed, and my bones would make cracking sounds, which would provide some relief. Now, the symptoms are similar to before.

When I press on it, the pain is around a 5 on a scale of 1 to 10. I don't feel much when I don't touch it, but I can't maintain an upright posture for long when sitting, as it becomes sore after a while.

There is no pain in other areas, and I don't have any other medical conditions.

I haven't been to the hospital for this issue yet.

**The doctor's answer on the website**

Based on your sedentary work habits and current clinical symptoms, the possible conditions to consider are lumbar disc herniation or discogenic low back pain. These conditions typically present as recurrent and long-term lower back pain, which worsens after prolonged sitting or standing. In addition to lower back pain, discogenic low back pain often includes referred pain in the groin area, buttocks, or anterior thigh, though it typically does not extend below the knee.

I would recommend starting with a complete lumbar spine X-ray and lumbar spine MRI to establish a definitive diagnosis. Secondly, it's advisable to modify your sedentary lifestyle habits, engage in regular physical exercise, and finally, for most patients with this type of low back pain, conservative treatments such as medication, physical therapy, bed rest, massage, and lumbar traction can be effective treatment methods. Specific medication guidance will be provided later.

62.

Symptoms and Duration of Affliction: Neck and shoulder soreness for 11 days. The pain is most severe in the morning upon waking up, primarily on the entire right side of the neck and right shoulder, sometimes waking me up at night. Any movement of the head or sleeping on my side causes a pulling and painful sensation. After getting up and moving around slowly, the pain tends to ease significantly. During the day, there is discomfort in the neck when tilting it downwards or upwards, as if a nerve is being compressed. These past few days, I've also started feeling some pain on the left side, but the right side is still more painful. A similar issue with left neck and shoulder pain occurred in August for about a week, which was relieved after receiving acupuncture. However, since then, I've been experiencing stiffness and soreness in my neck and shoulders for about six months. Due to my frequent use of mobile phones and computers for work, my neck hasn't been comfortable, and the discomfort has worsened over the past six months. This current episode of pain started on January 5th and has persisted until now.

Medical Visits and Medication: None.

Questions Requiring Answers: Do I need to undergo imaging, take medication, or pursue other treatments?

**The doctor's answer on the website**

Hello, considering your medical history and clinical experience, the initial consideration for your discomfort in the neck and shoulder area is cervical spondylosis and a reduction in the physiological curvature of the cervical spine. It would be advisable to undergo a cervical spine X-ray or MRI scan to assist with the diagnosis. Prolonged periods of sitting, standing, or looking down can trigger this condition. It's not severe, and you can start with conservative treatment following the recommendations below to promote recovery.

Conservative treatment recommendations:

Currently, when sitting for extended periods or standing, you can use a cervical spine automatic traction device or neck brace (a crucial treatment measure, available for purchase online) to alleviate the pressure on the intervertebral discs and promote recovery. If possible, try to minimize prolonged sitting and standing and spend more time in a semi-recumbent position. The better you rest, the faster the recovery.

You can apply fluorouracil ointment or diclofenac gel locally to the neck for anti-inflammatory and pain relief. For oral medication, you can take ibuprofen or celecoxib capsules to reduce inflammation and alleviate muscle discomfort, speeding up recovery.

You can perform neck massages (using a simple neck massager available for purchase online, 3-4 times a day) and infrared therapy (available for purchase online, 3 times a day for 20-30 minutes per session, with the lamp placed about 30cm away; infrared therapy is easy to operate and highly effective) to improve microcirculation through physiotherapy.

Maintain a balance between work and rest, try to avoid prolonged neck flexion, and engage in neck extension exercises to promote recovery.

Be consistent with your treatment, with one course of treatment per week, and you will experience faster relief of symptoms.

63.

Symptoms and Duration of Affliction: Yesterday, around two or three o'clock in the afternoon, pain started below the left clavicle, spreading to the neck and the area behind the ear by nine or ten o'clock in the evening. I woke up in the middle of the night with occasional pain in the area above the chest, connected to the clavicle. While the pain isn't extremely severe, it has been persistent since yesterday afternoon. My left arm also feels somewhat weak, which is distressing. Less than half a year ago, I experienced a similar pain in the same area, and it seemed to improve after a night's sleep. (I've been working rotating shifts and sitting at a computer for twelve hours recently, and I'm not sure if this could be a contributing factor.)

Medical Visits and Medication: I haven't seen a doctor. This morning, around seven o'clock, I took one sustained-release ibuprofen capsule, but the effect wasn't significant, and the pain persists.

Questions Requiring Answers: 1. Due to the severe pandemic situation, I cannot leave my workplace, and nearby hospitals are also dealing with high caseloads. I'm afraid to go to a hospital. Can you please suggest ways to alleviate and treat the pain? If medication is necessary, please recommend several options that can be ordered online for delivery. 2. If the symptoms subside and disappear in the future, and the pandemic situation improves, would it still be necessary to go to the hospital for an examination? I've had high uric acid levels once or twice before, but they returned to normal after dietary adjustments. Could this pain in the mentioned area be related to uric acid or heart issues? 3. Are there any specific precautions or considerations in my daily life that I should be aware of? Thank you, doctor.

**The doctor's answer on the website**

Based on the information you provided, it seems likely that your current condition is related to your cervical spine. From your symptoms, it appears that the initial pain below the left clavicle may be due to strain on the chest and rib joint area caused by prolonged periods of forward head posture or slouching, leading to inflammation and pain. The pain in the neck and behind the ear could be attributed to muscle strain around the cervical spine. Your recent prolonged periods of desk work, including overnight shifts, may have contributed to this issue as well.

If it's possible, you should consider getting a cervical spine X-ray or MRI to assess the severity of your current cervical spine problem. If it's not too severe, conservative treatment can be very effective. Given that you've experienced similar symptoms before and they improved after rest, it's a positive sign. However, if you can't currently access medical imaging due to the pandemic, you should focus on addressing the cervical spine issue with the following recommendations, which should aid in your recovery:

Rest is crucial. Avoid prolonged desk work and consider using a neck brace to stabilize and protect your cervical spine for about a week. This will help relax your muscles and maintain the correct posture. Use a cervical pillow while resting, as it can help relax the muscles around your neck and behind the ears, contributing to your recovery. During work, maintain proper posture, keep your head up, and take breaks to stretch and relax every half to one hour.

Consider undergoing physical therapy either at a clinic or using a self-administered therapy lamp at home, such as infrared therapy. If these options are not available, you can use a hot water bottle for 5-10 minutes, three times a day.

Medication is essential. You can apply fluorouracil gel externally on the painful areas and consider taking oral medications like ibuprofen, celecoxib, and methylcobalamin, which tend to be effective. These medications can be ordered online through various platforms.

By following these recommendations, I believe your condition should gradually improve and recover. Don't worry too much. After recovery, it's important to continue protecting your cervical spine, adjusting your posture, and engaging in exercises that benefit the neck, such as neck exercises or, if possible, playing badminton or swimming, as these activities can be highly beneficial. Consistency is key for long-term results. At the moment, it doesn't appear that your elevated uric acid levels are directly related to your current symptoms, as the pain you're experiencing is not typical of gout, which typically affects the feet or knees rather than the neck. As for heart issues, if you didn't have any previous heart problems and your blood pressure is normal, your current symptoms do not strongly indicate heart-related concerns. However, excessive workload and exhaustion can have an impact on your heart, so it's essential to find a balance between work and rest.

These are my initial assessments and treatment recommendations based on your consultation. I hope they are helpful. If you have any further questions, please feel free to ask, and I will respond promptly.

64.

Hello doctor, I just turned 23 years old this year, and I'm a male. About three days ago, I noticed discomfort in my neck, specifically around two and a half to three fingers above the cervical spine. When lying flat or lifting my head, I can feel significant soreness and pain in that area. Prior to this, I had symptoms of numbness in my left scapula, almost like an electric shock, and both shoulders felt sore and swollen. I tried doing chest-expanding exercises to relieve it, but I couldn't continue for long as the discomfort became too pronounced. I have very little physical activity in my daily routine and frequently use smartphones and computers. Another bad habit I have is that I tend to prop my head up while sleeping. Since my pillow is quite soft, I often wake up with my upper body in a semi-sitting position, with my neck unsupported and my head resting against the headboard. I've been doing this for a while without paying much attention, thinking it was just a habit, until this recent issue with my neck. Currently, I haven't experienced symptoms like dizziness or nausea, but I do get slight headaches if I stay in my room for an extended period, and they tend to subside when I go outside for fresh air. Additionally, when I woke up today lying flat on my back, I felt a slight pain at the back of my head, almost like a nerve pain, but it disappears when I lie on my side. Due to the pandemic, I'm unable to go to the hospital for X-rays or scans, so I wanted to ask here if the doctor could provide a general assessment and offer some advice on how to alleviate the symptoms. I appreciate your help very much!

**The doctor's answer on the website**

I'm sorry for keeping you waiting. Based on the information you provided, it seems that you are currently experiencing an issue with your cervical spine. The reason for this is likely related to your poor lifestyle and work habits. For instance, your lack of physical activity, frequent use of smartphones and computers, and sleeping habits may have contributed to this condition. You mentioned that you often prop your head up while sleeping, which results in a semi-upright position with your neck unsupported and your head resting against the headboard. Over time, this posture can strain the muscles in your neck, shoulders, and back, leading to fatigue and discomfort, as well as headaches.

Currently, you haven't mentioned any symptoms of nerve compression, such as numbness or tingling in your hands or feet. From this perspective, your cervical spine issue doesn't appear to be severe. With proactive treatment and lifestyle adjustments, your symptoms should gradually improve, and as long as you take measures to protect your cervical spine and engage in neck-friendly exercises, you should be able to prevent future recurrence of these symptoms.

Here are some recommendations for your treatment:

Rest and adjust your work and lifestyle habits. Maintain proper posture while working, with your head up, chest out, and eyes at eye level. Consider using stands or ergonomic chairs to provide adequate support for your neck, shoulders, and back. Take short breaks every half hour to an hour to stretch and relax. When sleeping, make sure to use a neck pillow and avoid keeping your head in an elevated position for extended periods.

Consider seeking physical therapy if possible. While you mentioned difficulties with visiting a hospital due to the pandemic, you can perform heat therapy at home using a hot water bottle for 5-10 minutes, three times a day, which can help alleviate your symptoms.

Medications such as topical ibuprofen gel patches (like Voltaren), oral muscle relaxants (such as Cyclobenzaprine), and pain relievers (e.g., acetaminophen) may be beneficial in managing your symptoms. Please consult a healthcare professional for proper medication recommendations.

After your symptoms improve, engage in regular neck exercises, such as neck stretches and strengthening exercises. You can find instructional videos for neck exercises online. Additionally, activities like playing badminton or swimming can be beneficial for your neck health if practiced consistently.

Your current condition appears to be a cervical spine issue, but it doesn't seem too severe, especially since you haven't experienced nerve compression symptoms. While it may not be possible to undergo imaging tests at the moment, you can address your symptoms by following the advice provided. Over time, your symptoms should gradually improve, and you shouldn't worry excessively.

65.

Hello doctor, I usually dare not lower my head to read books or use my phone because it makes me feel nauseous and my neck becomes very uncomfortable. I feel extremely uncomfortable and it takes several days to recover from this abnormal sensation. Additionally, when I raise both hands up, I feel a more comfortable sensation in my neck and above my shoulder blades. I have to be very careful not to lower my head too much, as it makes me feel unwell. Should I go to the hospital for further examination, and how can I read books, use my phone, or use a computer normally like other people?

**The doctor's answer on the website**

Based on the information you provided, it appears that your current symptoms are typical of cervical spine issues. The fact that you avoid lowering your head to read books or use your phone due to nausea and discomfort suggests that there might be muscle strain in your neck or alterations in the natural curvature of your cervical spine. Given the pronounced and prolonged nature of your symptoms, it is advisable to seek medical attention. I recommend getting an X-ray of your cervical spine, and if possible, an MRI for a clearer assessment. If the imaging results show changes in the cervical spine curvature, bone spurs, or disc protrusions, your diagnosis of cervical spine issues would be confirmed.

However, if the imaging results do not reveal severe issues, which is likely since you haven't mentioned nerve compression symptoms such as numbness, your condition may not be too severe. Your symptoms of discomfort and nausea when lowering your head are consistent with cervical spine problems. It seems you haven't undergone any treatment for this condition yet, so it's essential to address it now.

Here are some treatment recommendations, and I believe they should be helpful in your recovery:

Rest and Adjust Work/Lifestyle Habits: Since you currently experience discomfort when lowering your head, it's crucial to rest adequately. Consider using a neck brace to support your cervical spine. When working, use stands or ergonomic setups to maintain a proper posture—head up, chest out, and eyes at eye level. Take short breaks every 30 minutes to an hour to stretch and relax. Avoid maintaining a prolonged downward head position. When sleeping, use a neck pillow to allow your cervical spine to relax. These habits should be maintained to prevent a recurrence of cervical spine issues.

Physical Therapy: It's advisable to seek physical therapy if possible. Infrared therapy or ultrasound treatments can be beneficial in your recovery. Consistent therapy sessions can help alleviate your current symptoms and promote healing. Consider undergoing 1-2 treatment courses.

Medications: You might benefit from medication. I recommend taking oral muscle relaxants (e.g., Cyclobenzaprine), pain relievers (e.g., acetaminophen), and anti-inflammatory medications (e.g., ibuprofen) to manage your symptoms. Consult a healthcare professional for the appropriate medication and dosage.

Exercise: After some time of treatment and symptom improvement, engage in regular neck exercises. Neck stretches and strengthening exercises can be beneficial. You can find instructional videos online. Additionally, participating in sports like badminton or swimming can be helpful for your neck health if done consistently.

While your condition seems to indicate cervical spine issues, it's crucial to get a medical examination to understand the extent of the problem. However, as long as the imaging results don't reveal severe issues, following the above recommendations, including ongoing lifestyle adjustments and exercises, should lead to gradual symptom relief and recovery. Please don't worry excessively, and take proactive steps to address your cervical spine concerns.

66.

My wife recently experienced discomfort in her right hip joint, but imaging tests did not show any issues. In the past few nights, she has been experiencing numbness and even some pain in her right hand and foot, which partially improves when lying flat. What could be the cause of this situation?

**The doctor's answer on the website**

Based on the information you provided, it seems that your wife's condition may be related to her daily work and lifestyle habits. She recently experienced discomfort in her right hip joint, which didn't show any issues on imaging tests. However, in the past few nights, she has been experiencing numbness and occasional pain in her right hand and foot, which partially improves when lying flat. This suggests that her symptoms are more pronounced when she sleeps on her side, possibly indicating a connection to her cervical spine.

I suspect that her condition could be linked to her prolonged sitting and posture, such as hunching over while working, which may have led to cervical spine issues. Additionally, extended periods of sitting or sitting with legs crossed can strain the muscles around the hip joint.

I recommend that she seek medical attention to have a thorough examination. The doctor can assess the specific problem areas and may consider conducting an MRI of her cervical spine to obtain a clearer diagnosis. If there are mild issues found in the imaging, it could explain her current symptoms. However, even if there are no severe problems detected, proactive treatment, lifestyle adjustments, and exercises can help with symptom relief and recovery.

For the next steps in treatment, it's essential to adjust her work and lifestyle habits. She should avoid prolonged sitting and maintain proper posture, using supports and ergonomic setups if necessary. Taking short breaks every 30 minutes to an hour is crucial to prevent strain. When sleeping, she should use an appropriate neck pillow to relax her cervical spine.

Physical therapy can be beneficial. Infrared therapy or ultrasound treatments may help relieve her symptoms and promote healing. Consider undergoing 1-2 treatment courses.

Medications such as oral muscle relaxants, pain relievers, and anti-inflammatory drugs can provide relief. Consult a healthcare professional for the appropriate medication and duration.

Once her symptoms improve, she should engage in regular neck exercises and consider participating in physical activities like jogging or swimming to enhance her overall health and prevent symptom recurrence.

While her condition may be related to her habits and posture, a medical evaluation is crucial for a precise diagnosis and tailored treatment plan. Encourage her to seek medical attention to address her symptoms effectively.

67.

I underwent a medical examination in April of last year and learned that I have a straightening of the physiological curvature of my cervical spine. My main symptom is experiencing numbness in the ring and little fingers of both hands when lying flat to sleep at night, which often wakes me up. This symptom occurs at least 4-5 days a week. I spend a lot of time sitting in front of a computer due to my studies, but I have always elevated the computer screen to eye level and hold my phone at eye level when using it. Over the past year, I have reduced my head-tilting and frequently stretch my neck by looking upward. However, I have noticed that whenever I lie flat to sleep, my left hand's ring and little fingers become numb after a while, and the same happens occasionally in my right hand, but less frequently. If I sleep on my side, I don't experience numbness. Apart from numbness during sleep, I sometimes hear a mild clicking sound when tilting my head backward, and I currently don't have any other adverse symptoms. I would like to know if my condition requires surgery, or if it can be managed through daily care and exercise.

**The doctor's answer on the website**

Hello, based on your medical history and clinical experience, your current discomfort appears to be related to cervical spondylosis due to prolonged sitting, but it doesn't seem to be severe enough to warrant surgical intervention. It is likely manageable through daily care and specific neck exercises. Here are some treatment suggestions to facilitate your recovery:

Treatment Recommendations:

You can consider wearing a cervical spine self-traction device or neck brace on your neck during long periods of sitting or standing. This is a crucial treatment measure and is recommended for at least 10 hours a day (available for purchase online). It helps relieve pressure on the intervertebral discs and promotes recovery. However, if possible, try to reduce prolonged sitting and standing, take breaks, and ensure good rest, as the better you rest, the faster the recovery.

You can apply local anti-inflammatory and pain-relieving ointment containing diclofenac to your neck and take nutritional supplements like methylcobalamin tablets for nerve support. You can also consider taking oral anti-inflammatory and pain-relieving medication such as Tolfenamic acid to expedite recovery.

You can perform neck massages using a simple neck massager (available for purchase online, 3-4 times a day) and infrared therapy (available for purchase online, 3 sessions per day, each lasting 20-30 minutes, with the infrared lamp placed 30 cm away). Infrared therapy is straightforward to administer and highly effective. These therapies can improve microcirculation and alleviate tension in the soft tissues of the neck.

Maintain a balance between work and rest, and try to avoid prolonged periods of looking down. Engage in neck exercises and movements, especially those that involve tilting your head backward, to promote recovery.

Consistency is key; adhere to the treatment plan. A treatment course typically lasts for 4 weeks for noticeable symptom improvement.

By following these recommendations, you can enhance your chances of recovery and alleviate your current discomfort associated with cervical spine issues.

68.

I am a 54-year-old teacher, and in recent years, cervical spine CT scans have shown disc protrusion in my neck. I have experienced severe dizziness accompanied by vomiting during two episodes (most recently just a few days ago). Additionally, I often experience headaches due to insufficient blood supply to the brain. I have not used medication but have undergone some physical therapy. Could you please provide me with a treatment plan?

**The doctor's answer on the website**

Hello, based on your medical history and clinical experience, it appears that you may be suffering from cervical spondylosis, which is causing vascular and nerve compression. This condition is relatively severe and requires a comprehensive treatment approach. Currently, I can provide you with the following treatment recommendations to promote your recovery:

Treatment Recommendations:

You should consider wearing a cervical spine self-traction device or neck brace around your neck during extended periods of sitting or standing. This is a crucial treatment measure and is recommended for at least 10 hours a day (available for purchase online). It helps alleviate pressure on the intervertebral discs and facilitates recovery. However, if possible, try to minimize prolonged sitting and standing, take breaks, and ensure proper rest, as better rest leads to quicker recovery.

You can apply local anti-inflammatory and pain-relieving ointment containing diclofenac to your neck and take nutritional supplements like methylcobalamin tablets to support nerve health. Additionally, oral anti-inflammatory and pain-relieving medications like Tolfenamic acid, Ginkgo Biloba supplements to improve blood circulation to the head, and Betahistine to alleviate dizziness can expedite your recovery.

You can perform neck massages using a simple neck massager (available for purchase online, 3-4 times a day) and other physical therapies to improve microcirculation.

Maintain a balance between work and rest, and try to avoid prolonged periods of looking down. Engage in neck exercises and movements, especially those involving tilting your head backward, to promote recovery.

Consistency is key; adhere to the treatment plan. A treatment course typically lasts for 4 weeks for noticeable symptom improvement.

69.

Hello doctor, since April 19th, I have been feeling some discomfort and soreness in the muscles and bones of my right upper limb while working during the day. I initially attributed it to work-related factors and didn't pay much attention. However, I woke up in the middle of the night with pain, which came in waves, and massaging it made it worse. The next day, I felt a slight ache when I massaged it again, and this discomfort continued. I also started experiencing occasional soreness in my left hand. Every day, both hands would occasionally feel sore and achy, with the pain coming in waves. The discomfort was in different parts of the upper or lower limbs, but not in the joints. Occasionally, my legs would also suddenly ache, but it was not as pronounced as the discomfort in my hands. I have hyperthyroidism, which I've had for almost three years, and it's currently well controlled with regular check-ups. I have been taking methimazole. I also have a tendency towards anxiety and palpitations. Since last year, the doctor prescribed me Xanax, which I've been taking until now. I'm not sure if taking too much of this medication is causing these symptoms of limb soreness and fatigue. Right now, I'm troubled by the soreness and discomfort in my limbs, especially in my arms. I'd like to ask the doctor what could be the cause of these symptoms and what should I do about it? I was diagnosed with chronic gastritis two months ago, and I occasionally take some medication for my stomach, but it doesn't seem to be related to my current condition.

**The doctor's answer on the website**

Based on the information you provided, it's important to consider whether your current symptoms are related to your medication or hyperthyroidism. You mentioned that you've been reducing your medication recently, and if your symptoms have worsened since then, it might be necessary to reassess your thyroid function. If you haven't had a recent check-up, it's advisable to get one to confirm if your thyroid levels are within the desired range. If your thyroid levels are stable and not contributing to your symptoms, then we should explore other potential causes.

Given your description of working habits, including prolonged sitting and computer or smartphone usage, it's possible that your symptoms are related to musculoskeletal issues, particularly in the neck and possibly the lower back. These symptoms may be associated with poor posture and prolonged periods of immobility. Therefore, if your thyroid check comes back normal, you could consider consulting a orthopedic specialist. They can assess your musculoskeletal health and perform imaging tests like a CT scan or MRI of the neck and lower back to check for issues such as disc protrusions or nerve compression that could explain your symptoms.

Treatment options would depend on the findings of these tests. If musculoskeletal issues are contributing to your symptoms, conservative measures can be taken, such as adjusting your work habits. This may include avoiding prolonged sitting, maintaining good posture, and taking regular breaks to stretch and move around. Sleeping on a firm mattress with a cervical pillow to support your neck may also help.

In addition to lifestyle modifications, physical therapy, such as infrared therapy or acupuncture, can be considered to improve blood circulation and relieve muscle tension. Medications like acetaminophen or non-steroidal anti-inflammatory drugs (NSAIDs) can provide pain relief. If your symptoms are primarily related to muscle strain and posture, low-impact aerobic exercises like walking, swimming, or yoga can be beneficial. However, it's important to start slowly and gradually increase the intensity of your exercise routine to avoid exacerbating your symptoms.

Furthermore, considering your history of anxiety and medication for it, it's worth addressing any potential psychological factors contributing to your symptoms. Stress and anxiety can manifest as physical symptoms, and managing your emotional well-being is an integral part of your overall health.

70.

Hello, I'm not a doctor, but I can try to provide some general information based on the symptoms you described. It's important to consult a healthcare professional for a proper diagnosis and treatment plan.

Your mother's symptoms, including numbness in the right hand, along with pain in the forearm, upper arm, shoulder, and neck, could be related to various factors. One possibility mentioned by doctors is cervical spine (neck) issues, which might be causing pressure on the nerves. Other potential causes could include musculoskeletal issues or even conditions like carpal tunnel syndrome.

Since you mentioned that your mother is currently taking "追风透骨胶囊" (a Chinese herbal medicine), it's essential to consult with a healthcare provider before starting or continuing any medication to ensure it's safe and appropriate for her condition.

When she retired, she worked at a parts factory (rubber products) for about four years. She had a habit of playing Mahjong and used her phone for approximately 4 hours a day. During the summer, she mostly lived in an air-conditioned environment. She didn't stay up late, and her sleep and appetite were normal. The numbness in her hand gradually appeared, and sometimes it worsened when she lifted heavy objects, which she didn't experience before. She is right-handed.

She feels that the numbness encompasses the entire palm of her hand, not just a single finger. She believes it's caused by issues with her cervical and shoulder vertebrae. The main sensation in her hand is numbness, but her shoulder, neck, forearm, and upper arm mainly experience aching pain. During the day, the symptoms are not very severe, but they worsen at night when she sleeps. She doesn't experience numbness in her face or lips, and her mental state and appetite are normal. However, she has had occasional constipation for a while (constipation has been a chronic issue for her). Currently, the symptoms don't significantly impact her daily life, but the numbness is uncomfortable. She had a previous uterine fibroid surgery, and she no longer menstruates.

In addition to the numbness in her hand, she experiences noticeable pain in her shoulder and neck, occasionally accompanied by dizziness. Changes in weather can exacerbate the pain in her shoulder and neck, which in turn worsens the symptoms in her hand. The conclusion from the community clinic was that it's a cervical spine issue. She was prescribed several medications to alleviate neck and shoulder pain, and they provided some relief.

Other information: She had a uterine fibroid surgery to remove the uterus about six months ago.

**The doctor's answer on the website**

There are various reasons for upper limb pain, arm numbness, and hand numbness:

The most common causes include cubital tunnel syndrome (as well as carpal tunnel syndrome), incorrect sleeping posture or maintaining the same position for an extended period during sleep. For example, some patients use their wrists and elbows frequently in daily activities, leading to the "wear and tear" narrowing of the nerve pathways, resulting in localized pressure, most commonly seen as carpal tunnel syndrome or cubital tunnel syndrome. Some patients who favor sleeping on their left or right side may compress the hand nerves at the elbow or wrist, causing nighttime numbness that improves with movement upon awakening. In severe cases, patients may experience muscle atrophy in the hand, hand weakness, and limited mobility, necessitating further examination. For some patients, after conservative treatments such as rest, anti-inflammatory pain relief, nerve-nourishing medications, and heat therapy have proven ineffective, surgery may be considered.

Cervical spondylosis can also lead to hand numbness. Prolonged sitting and working with the head lowered can cause disc herniation in the cervical spine, which may compress the cervical nerve roots, resulting in neck and shoulder muscle pain, numbness, weakness in the upper limbs, and even muscle atrophy. Given your mother's occupational habits and current symptoms, this possibility is currently the most likely.

Of course, there are other possibilities, such as stroke (cerebral infarction). Patients with high blood pressure, high cholesterol, and other risk factors for atherosclerosis may experience reduced blood flow and increased blood viscosity during sleep, leading to inadequate cerebral blood supply and symptoms such as limb numbness.

If a patient experiences persistent bilateral hand numbness, it could be due to peripheral nerve damage or compression, such as in diabetes, alcohol poisoning, uremia, vitamin deficiencies, drug toxicity, etc. Some diabetic patients with long-term uncontrolled blood sugar levels may develop peripheral nerve damage, resulting in numbness and sensory abnormalities, particularly noticeable during nighttime sleep. Additionally, if there is central nervous system involvement, such as damage to both brainstem sides, syringomyelia, or central nervous system infections, it can affect the lateral corticospinal tracts and result in numbness in both limbs (although this possibility is less likely in your mother's case).

Therefore, the causes of neck and shoulder pain and hand numbness are numerous, and many conditions require attention. Based on your mother's occupational habits and current symptoms, the most likely possibility at present is cervical spondylosis with nerve root compression. Current treatment recommendations are as follows:

a. Pay attention to rest and avoid prolonged periods of sitting with the head lowered while using a smartphone or playing Mahjong. Taking breaks and performing neck exercises can help alleviate symptoms. Ensure regular sleep patterns and avoid activities that exacerbate pain. Find a comfortable position to rest, and if lying down alleviates symptoms, consider spending more time in a reclined position.

b. Apply hot compress therapy to the sore or painful area of the neck and shoulders. Hot compresses are recommended 3-5 times a day for about 20-30 minutes each time. Adjust the temperature to your comfort level—avoid excessive heat to prevent local burns or inflammation of soft tissues. Hot compresses can improve local blood circulation and should be continued even after symptom improvement.

c. Neck traction therapy (recommended after a comprehensive examination to determine the extent of cervical spondylosis). Consider receiving traction therapy at a local tertiary hospital's rehabilitation department for more standardized treatment and to prevent complications related to traction.

d. For your current pain and numbness, you can consider the following medications (which can be taken simultaneously):

Naproxen sodium tablets (brand name: Aleve), which are commonly used anti-inflammatory and pain relief medications. The recommended dosage is one tablet in the morning, one at noon, and one in the evening, taken orally for 7-10 days.

Mecobalamin capsules, a nerve-nourishing medication. Take one capsule (50mg) in the morning, one at noon, and one in the evening, orally for 15-20 days.

These medications are commonly used in clinical practice and are generally safe. However, please review the medication instructions for usage and potential side effects. If you experience any discomfort during treatment, if your symptoms worsen during the course of treatment, or if there is no significant improvement after treatment, seek immediate medical attention and consult with a local hospital's orthopedic department for further examination.

e. After the acute phase has passed, it is essential to strengthen the muscles of the neck. This is crucial for your mother's recovery. Once the existing symptoms have disappeared and she has fully recovered, consider engaging in appropriate exercises to strengthen neck muscles, such as swimming.

I believe that with comprehensive treatment for some time, there should be effective results. If conservative treatment for 3-4 weeks does not lead to improvement or if symptoms worsen during treatment, further evaluation at a hospital with electromyography (EMG) and cervical magnetic resonance imaging (MRI) may be necessary to diagnose any nerve conduction issues causing pain and numbness.

71.

Hello, doctor. Recently, I'm not sure when it started, but before going to sleep, I've been feeling some discomfort in my right leg and right foot. In the past few days, I've noticed a slight ache in the little toe of my right foot, and my right arm and the little finger of my right hand also have a slight tingling sensation. There is also some mild soreness near the joints of my right leg. Occasionally, I experience slight twitching during sleep. Thank you!

**The doctor's answer on the website**

Hello, your symptoms are quite similar to cervical spondylosis and lumbar disc protrusion.

When there is a protrusion at these two locations, it can compress the nerve roots. Cervical disc protrusion can lead to neck pain and a tingling sensation extending to the fingers and arm. On the other hand, lumbar disc protrusion can cause radiating pain in the lower limbs, which is a typical symptom of sciatic nerve pain. If the protrusion is on the right side, it can result in radiating pain on the right side of the lower limb. Sciatic nerve pain can lead to pain radiating along the back of the thigh, outer side of the lower leg, and toes. All the pain along this pathway is indicative of sciatic nerve pain, which is a manifestation of lumbar disc protrusion. It can cause sensations of soreness, weakness, and numbness in the thigh and lower leg.

Have you had a lumbar MRI scan to assess the presence and severity of lumbar disc protrusion? If not, I would strongly recommend not delaying any further. It's advisable to schedule a lumbar and cervical MRI scan as soon as possible to assess the extent of spinal cord and nerve root compression, which will help determine the appropriate treatment strategy.

72.

Hello, Dr. Ni. Previously, during my time at school, I spent long hours using a computer, which led to discomfort in my neck and occasional numbness in my fingers. My neck discomfort had started during high school due to prolonged computer usage. I had purchased medicated patches and, at that time, went to the school hospital for an X-ray examination. The doctor informed me that I had a fairly severe case of cervical spondylosis, prescribed some medication, and advised me to be cautious with neck movements. During the holidays when I returned home, I didn't experience any discomfort in my neck or numbness in my hands since I didn't engage in prolonged desk work.

However, about ten days ago, I went to the driving school for training early in the morning, and it was quite cold. I had to wait outside for an extended period, and after returning home, I developed a headache and felt nauseous for about a day. The symptoms gradually subsided after resting. Then, last night around 10:30 pm, I experienced a heavy head, not exactly dizziness, along with nausea and a desire to vomit. I vomited twice, once around 11:30 pm and again around 1:00 am. After vomiting, I felt somewhat relieved, and the nausea decreased. Following the second episode of vomiting, I went to sleep. Yesterday afternoon, I spent a considerable amount of time on the computer, and for dinner, I had deep-fried rice dumplings and sweet potatoes (I'm not sure if this is related to the symptoms). It's important to note that during both instances of headache and nausea, I didn't experience any neck discomfort or hand numbness.

Additional information: I tend to prefer sleeping on my left side. Thank you.

**The doctor's answer on the website**

Based on the information you provided, it appears that you have a clear case of cervical spondylosis. This is because you have previously experienced neck problems, and you mentioned that the X-ray examination showed a fairly severe condition. You also spend a significant amount of time with your head in a downward position, which can lead to recurrent symptoms. During your breaks, when you have the opportunity to rest and avoid prolonged desk work, your symptoms tend to improve.

However, recently, possibly due to the weather or other factors, you have experienced symptoms that are different from your previous neck discomfort and hand numbness. These new symptoms include headaches, dizziness, nausea, and vomiting, which are also common symptoms of cervical spondylosis. This may indicate that your condition has developed further. Therefore, if these symptoms are recurring frequently, it's advisable to seek medical attention and undergo another cervical spine X-ray to assess the situation. If the X-ray shows that the problem is not severe, we can continue with conservative treatment.

It's essential to address the root cause of your condition, which is prolonged periods of using a computer, smartphone, or reading with your head in a downward position. If this issue is not resolved, your cervical problems are likely to recur. I recommend that you refrain from prolonged computer use, smartphone usage, or reading from now on. If you must engage in these activities, consider using a support or stand to elevate your books, computer, or smartphone screen to eye level. Additionally, take short breaks every half hour to stretch and move around. If you experience significant dizziness, it's a good idea to use a neck brace to stabilize your cervical spine and promote better recovery.

Physiotherapy, such as acupuncture or infrared therapy, can be beneficial, and I recommend seeking such treatments in a hospital. Medication may also be necessary, and I suggest trying oral methylcobalamin, cyproheptadine, and ginkgo biloba leaf tablets. These medications can help alleviate your current symptoms. You can try them for about a week, and if they are effective, continue using them until your symptoms have largely subsided.

Regarding your sleep position, there is no specific requirement, but a good neck pillow is important. I recommend purchasing a cervical pillow, which can provide support whether you sleep on your back or side. Once your symptoms have improved considerably, it's essential to engage in regular physical exercises that are beneficial for your neck, such as playing badminton or swimming. This will strengthen the muscles around your neck and better protect your cervical spine, aiding in your recovery and preventing future relapses.

73.

Symptom Description: I am a 20-year-old college student. Lately, I have been experiencing unexplained headaches, and based on online research, I suspect it may be related to my cervical spine. In the past, my posture has indeed been detrimental to my neck. Over the past few days, whenever I spend a prolonged period with my head in a downward position, it leads to fatigue around my neck, noticeable headaches, and occasional dizziness and nausea. On a related note, does this have anything to do with scoliosis? I was diagnosed with scoliosis five or six years ago, but it hasn't had a significant impact.

Duration of the condition: In the past two weeks.

Medication: Currently not using any treatment.

Additional Information:

No other symptoms, just headaches, occasional neck fatigue, and intermittent dizziness and nausea.

I often stay up late, typically going to bed around 1 or 2 AM.

No numbness in the limbs.

I am a junior in college, and I spend long hours with my head down studying.

Resting helps provide relief, but I easily experience discomfort in my neck after just an hour or two of looking down. However, it doesn't always trigger a headache; it occurs approximately 20% of the time.

My neck can be moved in any direction without any issues or pain.

This condition started only in the past few days. I have never had these symptoms before, so I have never undergone any tests or treatments.

Do I need to go to the hospital for an examination? If so, what should I get checked?

**The doctor's answer on the website**

Based on the analysis of your condition, the symptoms you are currently experiencing are likely related to cervical spine issues. Prolonged periods of staying up late, working or using your phone with your head down can lead to an abnormal straightening of the natural curvature of the cervical spine. This can cause tension and spasms in the neck and shoulder muscles, especially the trapezius muscle. As a result, you may experience discomfort, soreness, or pain in the neck area, particularly when exposed to cold temperatures. These symptoms are typical early signs of cervical spine problems. Your symptoms appear to improve when you rest, allowing your muscles to relax. At this stage, there is no immediate need for specific medical tests or undue worry.

However, it is essential to treat this condition properly to facilitate a speedy recovery. The dizziness and nausea you mentioned may be related to autonomic nervous system disturbances caused by chronic sleep deprivation from staying up late.

Here are some treatment recommendations for your current condition:

Prioritize rest and avoid staying up late. Maintain a regular sleep schedule and avoid extended periods of computer or phone usage, which involves keeping your head down. Maintain proper sitting posture and take breaks to stretch and relax your neck muscles if you work on a computer for an extended time. Ensure you have an appropriate pillow or consider using a cervical pillow to support your neck.

Continue with physiotherapy, especially targeting the trapezius muscle. You can use infrared therapy lamps or seek traditional Chinese medicine treatments like massage, acupuncture, or cupping to effectively relieve muscle tension. This can be done for an extended period.

Consider using topical analgesic gels or patches containing flurbiprofen for local pain relief. Additionally, you can take oral analgesics like ibuprofen (sold as "Xilebao") to alleviate your symptoms.

Following the above treatment plan, your symptoms should improve quickly. Do not overly worry. After recovery, continue to protect your cervical spine by minimizing excessive head-down positions and participating in physical exercises that strengthen your muscles, such as badminton or swimming. Consistent long-term adherence to these practices will yield positive results. Pay attention to keeping your neck warm while sleeping at night.

Thank you for your trust, and I wish you a speedy recovery!

74.

Hello, doctor. My father, 68 years old, has been experiencing pain in his right knee for about a month. The pain occurs when he walks uphill but not downhill. He also experiences pain when bending his knee. He has seen a doctor and tried various Western and Chinese medications for joint issues, but there has been no improvement. Could you please tell me what might be the underlying condition?

Additional Information:

There is no stiffness, misalignment, or cracking of the knee.

There is slight swelling, and pressing on the knee causes some pain.

Occasionally, the tendons next to the knee may also be painful.

The most noticeable point of pain is on the inner side just below the knee where there is a protruding bone.

Thank you.

**The doctor's answer on the website**

Hello, doctor. My father, 68 years old, has been experiencing pain in his right knee for about a month. The pain occurs when he walks uphill but not downhill. He also experiences pain when bending his knee. He has seen a doctor and tried various Western and Chinese medications for joint issues, but there has been no improvement. Could you please tell me what might be the underlying condition?

Additional Information:

There is no stiffness, misalignment, or cracking of the knee.

There is slight swelling, and pressing on the knee causes some pain.

Occasionally, the tendons next to the knee may also be painful.

The most noticeable point of pain is on the inner side just below the knee where there is a protruding bone.

Based on the symptoms described, I suspect the diagnosis could be "Degenerative Osteoarthritis of the Knee Joint."

Degenerative osteoarthritis of the knee joint, also known as osteoarthritis or degenerative joint disease, is common in individuals over the age of 50. It is typically associated with factors such as age, body weight, gender, and occupation. The causes of osteoarthritis include changes in cartilage metabolism and nutrition, long-term weight-bearing, uneven joint surfaces, and chronic overuse or strain, among others.

In the early stages of degenerative osteoarthritis, patients may experience knee pain, particularly during activities that put stress on the knee joint, such as walking uphill. The pain is often intermittent and can improve with rest. Without early treatment, the condition may progress. In the mid-stage, pain can occur during regular walking, and the knee may feel weak and fatigued, with aching in the affected area. Typical symptoms of degenerative knee osteoarthritis include knee pain, joint deformity, swelling, recurrent swelling, joint crepitus (cracking or popping), and, in some cases, joint locking and limited mobility. Patients often find it difficult to squat, especially during the initial rise from a seated position, and may experience intermittent pain that improves with movement.

Additionally, consideration should be given to the possibility of adductor tendonitis. The adductor muscles are located on the inner side of the thigh, just above the knee, and adductor tendonitis can occur due to excessive strain, especially in individuals who engage in activities like running. Symptoms of adductor tendonitis include pain on the inner or outer side of the knee, swelling, and limited joint mobility, with increased pain during leg adduction or abduction. Tenderness may be felt at specific points on the inner or outer sides of the knee.

Finally, it's essential to evaluate the potential for chronic soft tissue injuries around the knee joint, such as meniscus tears, collateral ligament injuries (medial or lateral), or anterior cruciate ligament (ACL) injuries. These conditions may present with specific symptoms and should be considered, especially if the current symptoms persist.

Preventing the progression of knee joint degenerative osteoarthritis is crucial. Recommendations for prevention and management include maintaining proper posture during activities, avoiding prolonged squatting, and ensuring appropriate footwear with cushioned soles. Patients should refrain from excessive stair climbing, hiking, prolonged standing, and heavy lifting to reduce stress on the knee joint. Regular exercise to strengthen the muscles around the knee, such as swimming and walking, can be beneficial.

In colder weather, it's essential to keep the knees warm to prevent stiffness and increased pain. Maintaining a healthy weight is also important to reduce the burden on the knee joints. Dietary choices can support joint health by including foods rich in protein, calcium, and collagen, such as dairy products, legumes, seafood, seaweed, black fungus, and tendon meat.

Medication options may include oral non-steroidal anti-inflammatory drugs (NSAIDs) like ibuprofen or naproxen (taken with food to reduce potential stomach irritation), topical NSAID gels (e.g., flurbiprofen gel) for pain and swelling relief, and oral supplements like glucosamine, which may support cartilage health.

Thank you, and I hope this information is helpful. Wishing the patient a speedy recovery.

75.

Hello, I'm unable to bend my right knee, and my left knee also feels uncomfortable. When I squat down, my knees make a crackling sound, but it hasn't caused any issues. However, yesterday, around 6 PM, I felt a twist in my right knee, and since then, it's been painful whenever I try to bend it. Doctor, could this be a meniscus injury? Can it be diagnosed with an MRI scan? (Born in 1991, I haven't had any knee injuries before, but I walked a bit more yesterday, and I'm usually quite active...)

**The doctor's answer on the website**

Hello, considering your medical history and clinical experience, your current knee discomfort may be due to a meniscus injury, patellofemoral arthritis, or patellar maltracking. It is advisable to undergo a knee MRI scan to make a definitive diagnosis. In the meantime, you can follow the treatment recommendations below to promote recovery:

Conservative treatment suggestions:

You can apply topical anti-inflammatory and analgesic creams like diclofenac locally to relieve pain. You can also take oral nonsteroidal anti-inflammatory drugs (NSAIDs) such as celecoxib to enhance the anti-inflammatory and analgesic effects, helping alleviate pain and accelerate recovery.

Initially, use cold packs on the painful area. After 48 hours, switch to warm packs, maintain warmth, and consider infrared therapy to improve microcirculation and suppress the inflammatory response, promoting recovery.

For warm packs, use a warm towel to apply heat locally for at least two sessions each day, with each session lasting around 30 minutes, to expedite pain relief.

Infrared therapy involves using an infrared lamp to irradiate the painful area. This can help accelerate pain relief. Infrared lamp therapy (available for purchase online, costing approximately $100) should be done at least twice a day, with each session lasting about 30 minutes, and the lamp positioned approximately 30 cm away from the affected area.

You may consider wearing a knee brace to support the knee joint while walking, which can help distribute stress and alleviate symptoms, promoting recovery.

Reduce your level of physical activity, such as climbing stairs, and avoid prolonged standing, hiking, and long-distance walking. Prioritize conservative treatment and get plenty of rest. The better your rest, the faster and more effective your recovery will be.

Following the above treatment recommendations, you should start experiencing relief within two days. Each week constitutes one treatment cycle. Consistency in treatment is crucial for faster symptom relief and better recovery.

76.

Hello doctor, I would like to consult about a condition I've had for some time, which I believe started since I began exercising last year. I experience strong pain in the center of my chest, specifically in the area of the breastbone, when I perform chest expansion exercises or when I lower my head. There is also a popping sensation when I exert force (only when I lift my head and expand my chest). For instance, during chest expansion, I initially feel pain, and then the pain seems to disappear, but I can only maintain that position. Once I return to a normal position, the pain returns intensely, accompanied by a certain degree of chest tightness. When I press on the area during normal times, the pain is not very strong. If I avoid positions that involve opening up the chest, I don't have significant issues in my daily life.

Duration of the condition: Approximately six months.

**The doctor's answer on the website**

Based on the information you provided, I suspect that the pain in the middle of your chest may be due to costochondritis or costochondral dysfunction. It seems that when you initially started exercising about a year ago, you didn't have these symptoms. However, these symptoms have gradually appeared over the past six months, although it's possible that your timeline might not be entirely accurate. It's possible that you experienced these symptoms right from the beginning of your exercise routine, but you initially thought they weren't too concerning and would eventually resolve on their own. However, as it has persisted for such a long time and any chest-expanding or contracting movements still trigger pain, there might be two potential reasons for this.

Firstly, it could be related to your prior lack of exercise and prolonged periods of sitting, which may have resulted in stiffness in your costochondral joints. When you started exercising again, this stiffness could have led to localized pain, especially during movements with a large range of motion.

Secondly, it might be due to excessive or inadequate warm-up before your workouts. This could also lead to localized pain, including the popping sensation you mentioned.

Nevertheless, I want to reassure you that this issue doesn't seem to be very severe, and it's a common occurrence in clinical practice. Many individuals who sit for extended periods and suddenly stretch or perform chest-expanding movements experience chest pain or popping sounds. With appropriate treatment, this problem can gradually improve.

In terms of treatment, I recommend two main approaches. First, try to control the intensity and range of motion of your activities. Avoid movements that trigger pain and popping, allowing your costochondral joints to rest adequately. Over time, they should recover. Second, consider applying warm compresses for about 10-15 minutes before each workout. If you continue to experience pain, you can consider using topical medications like diclofenac gel to reduce local inflammation. By following these suggestions, your issue should improve, and you shouldn't worry too much.

77.

Hello doctor, four years ago, I experienced patellar lateral dislocation while playing basketball, and since then, I've been hearing noises from inside my knee joint. What could be the reason for this?

**The doctor's answer on the website**

Hello, your current symptoms are likely due to patellar maltracking leading to patellofemoral joint wear and tear.

Your injury from four years ago may have exposed an underlying issue with patellar tracking or, due to the significant force involved in the dislocation, resulted in scarring and poor healing of the medial patellar ligament, leading to maltracking.

While you may not be experiencing pain currently, prolonged wear and tear can lead to premature joint degeneration and the development of post-traumatic osteoarthritis.

Diagnosing patellar maltracking typically involves X-rays in various knee positions, MRI, and CT scans to make a definitive diagnosis. Treatment options depend on the evaluation of these imaging findings and may involve surgery. The specific surgical approach varies, whether it involves addressing the ligaments or the joint surfaces.

Considering your age, I recommend seeking further evaluation and consultation at a specialized orthopedic or sports medicine department at a tertiary hospital to determine the most appropriate treatment plan.

78.

Half a month ago, I developed tinnitus in my left ear. Recently, I received a 6-day course of dexamethasone at a local secondary hospital, totaling 55mg (15mg on the first day, 10mg on the following 2 days, and 5mg on the last 3 days, along with a 5mg injection behind the ear). I was also prescribed ligustrazine. I stopped taking dexamethasone three days ago. I've read online that the use of dexamethasone carries the risk of avascular necrosis of the femoral head. There have been cases where 70mg of dexamethasone over 9 days led to femoral head necrosis, so I am concerned. Is there a risk of femoral head necrosis with my steroid use, and should I take any preventive measures? Can oral calcium and vitamin D supplements like Caltrate D3 help prevent this?

**The doctor's answer on the website**

Hello, there are many reasons for avascular necrosis of the femoral head, such as taking hormone medications as you mentioned, experiencing trauma, alcohol and tobacco consumption, injuries, surgeries, and other factors that can lead to abnormal blood supply to the femoral head. However, we need to understand that the occurrence of femoral head necrosis due to hormone medications like dexamethasone is more common in patients with immune system or blood system diseases who require long-term hormone therapy. This is because long-term use of hormones can disrupt the body's clotting mechanism, leading to vasculitis of the blood vessels supplying the femoral head. This can cause blockage and damage to the blood vessels, leading to impaired blood supply to the femoral head, which is the mentioned injury to the nutrient arteries of the femoral head, and can further result in femoral head necrosis.

If you have only had a history of dexamethasone injections for 6 days, it is unlikely to cause avascular necrosis of the femoral head because the duration of hormone exposure is short and the dosage is relatively low. If you are concerned and want to be sure, you can go to the hospital for a hip joint MRI to observe if there are early signs of ischemic changes in the femoral head, such as cystic changes in the imaging findings. Generally, if these changes do not appear, there should be no major issues.

As for taking calcium and vitamin D supplements like Calcichew or Rocaltrol, they can only promote osteoblast bone formation and increase bone density, but they do not have a substantial impact on the blood supply to the femoral head. Therefore, they do not serve the purpose of preventing femoral head necrosis.

To prevent femoral head necrosis, it is important to avoid the factors I mentioned earlier that can lead to its occurrence. For example, you should refrain from smoking and drinking alcohol in the near term, discontinue the use of hormones, and avoid trauma to the femur.

You don't need to worry excessively; generally, a six-day history of hormone use should not be a problem. Wishing you a speedy recovery!

79.

Hello doctor, I'd like to ask on behalf of my father. My father is 58 years old and has had a history of lumbar disc herniation for over a decade. Two months ago, his knees started making a cracking sound, and since then, he has been experiencing pain. He has been taking glucosamine sulfate capsules for two months now, and the pain in his left knee has improved, but now his right knee is making cracking sounds and hurting, although it doesn't affect his mobility. Seven days ago, he felt a slight needle-like pain in his left buttock when lifting his left leg or applying pressure, and his left calf feels swollen. Both sides of his lower back muscles feel numb. I looked up online and performed the straight leg and four-finger tests for my father. The straight leg test was negative, and the four-finger test was negative on the right side. However, when doing the four-finger test on the left side, he felt a slight tingling sensation in the buttock. Does this indicate a problem with his hip joint and femoral head? He had a lumbar CT scan two years ago but hasn't had one recently. Due to the pandemic, he has been sitting at home watching TV, but he does go out for a walk every day.

**The doctor's answer on the website**

Hello, you are very thoughtful for conducting physical examinations yourself, and I commend your efforts.

Lumbar disc herniation can indeed cause leg pain and numbness. Given his medical history, it's possible that the pain is related to his lumbar condition. After the pandemic, it would be advisable to revisit the hospital for a comprehensive evaluation. The symptoms you've described do not seem to indicate an acute phase of lumbar spine disease. If the discomfort is relatively mild and does not significantly impact daily life, it may not require immediate intervention.

Lastly, based on your initial description, it appears that your father's knee may have pre-existing issues. At his age, common conditions to consider include osteoarthritis and meniscal injuries. During the pandemic, staying at home and continuing rest for another two weeks to a month, similar to what he did for the left side, might be beneficial. If the symptoms persist or worsen, then seeking medical attention for further evaluation is advisable. He can continue taking glucosamine sulfate; the typical treatment course is six weeks. It's worth noting that the effectiveness of this medication is subject to debate, so there's no need to extend the duration indefinitely. Continue for a few more months to assess its efficacy.

80.

In 2002, I was diagnosed with avascular necrosis of both hip joints, and I have not undergone surgery to date. The condition on the right side started earlier, and the femoral head has shown increased bone density, which appears to be a self-protective repair mechanism. On the left side, the condition developed later and has not shown any increase in bone density. Due to the lack of effective medication, I stopped taking medication for it five years ago. The pain in both hips has been tolerable and hasn't significantly affected my ability to walk.

However, in the past month, my wife has been giving me one tablet of vitamin D and calcium (Diquet) daily, and suddenly, the pain in my left hip has intensified. It has become severe during the day and particularly affects my ability to walk at night, disrupting my sleep. I suspect that this might be related to Diquet. Could it be a side effect or toxicity? Please analyze and provide guidance. Thank you!!!

**The doctor's answer on the website**

Hello, I apologize for keeping you waiting. Now, I'll address your questions:

You have a confirmed diagnosis of avascular necrosis of the femoral heads and have not undergone surgical treatment or received other medications. You mentioned that the right femoral head has increased bone density, which is a natural course of the disease. This represents self-repair by the necrotic femoral head, but it doesn't offer any protective benefit, nor does it slow down disease progression or alleviate pain.

According to your description, you experienced symptoms five years ago, and during this time, you have tolerated the pain in both hips without significant impairment of walking, which is why you haven't pursued other treatments. Currently, you're experiencing pain in the left joint while taking Diquet. Diquet contains vitamin D3 and calcium carbonate, taken as one tablet daily, and this is a very safe dosage that should not cause side effects. The pain you're currently experiencing is likely related to your underlying medical condition. Individuals with femoral head necrosis can occasionally experience acute pain episodes, often associated with physical exertion and insufficient rest. I recommend that you reduce your physical activity, get more rest, and consider taking oral pain relievers and glucosamine. Pain relievers not only alleviate pain but can also help reduce the aseptic inflammation associated with femoral head necrosis. You can take pain relievers on a long-term basis, and since the inflammation is aseptic, antibiotics are not needed. Glucosamine can provide some nutrition to the cartilage and has a certain therapeutic effect on femoral head necrosis. Pain relievers can be taken long-term, but since you have hypertension, there is a potential risk when combining pain relievers with certain hypertensive medications. Please provide a list of your current medications for further assessment. Glucosamine can be taken for approximately two months each year.

The pandemic has not yet ended, and for now, you can follow the treatment plan I've outlined. When the pandemic subsides, I recommend visiting an orthopedic specialist for a pelvic X-ray to assess the extent of your current femoral head necrosis. If there are changes in the appearance of your femoral head and if you continue to experience hip joint pain despite following my recommendations, it may be worth considering hip joint replacement surgery. Hip joint replacement for femoral head necrosis is a well-established procedure, and given that you are 50 years old, it is a viable option to consider.

81.

Hello doctor, my mother is 77 years old. She used to enjoy walking and would often go to the park for walks, averaging about 20,000 steps a day. However, recently, since the onset of winter, she has been experiencing pain in both of her knees, particularly in the knee bends. Walking has become difficult for her, and when she sits or lies down for a while and then tries to stand up, the pain becomes particularly intense. Taking a slow walk can provide some relief, but the pain is still there. I would like to know what kind of medical examinations she should undergo if we were to visit the hospital. Please help.

**The doctor's answer on the website**

Hello!

This condition is typically due to high joint stress and wear and tear, resulting in joint damage, with possible cartilage surface wear. If it's not too severe, it falls under the category of synovitis. When wear and tear is more severe, it's diagnosed as osteoarthritis. This condition is quite common in older individuals and is a degenerative disease. To confirm the diagnosis, you can get a knee joint X-ray in the anteroposterior and lateral views.

Treatment options for synovitis include the following:

First, maintaining warmth and providing heat therapy can help improve local blood circulation and promote tissue repair. A simple and effective method is to use a hot water bottle placed near the knee while sleeping.

You can also consider using a knee brace, which provides both warmth and support, reducing pressure on the knee joint.

Second, it's important to reduce excessive physical activity to minimize further wear and tear on the knee joint. You can consider taking oral glucosamine sulfate supplements, which can promote cartilage repair.

Third, for more severe cases, intra-articular injections of hyaluronic acid into the knee joint can provide relief. Many patients find relief after one or two injections. Surgery is an option for the most severe cases, but it doesn't seem necessary in your situation.

Fourth, calcium supplementation might be beneficial as knee synovitis can be related to osteoporosis. You can consider taking calcium and vitamin D supplements like Calcichew-D3 and Fosamax.

I wish you a speedy recovery!

82.

Hello, Dr. Zhang. Last week, I started noticing a slight, nagging pain in my tailbone after sitting on a hard chair and then standing up. I don't feel any discomfort when standing or lying down. When I press on the area, it also feels a bit uncomfortable. Is this condition likely caused by sitting on a chair, and is there any treatment or action I should take?

**The doctor's answer on the website**

Hello, in your case, it's likely that sitting on a hard chair for an extended period has caused some localized synovitis.

Treatment options primarily include the following:

Take short breaks and give the affected area some rest. Use a cushion or pillow for sitting, and try to avoid remaining in one position for too long.

Use pain relief medication such as Celecoxib or topical pain relief patches like Qizheng Stop Pain Patch.

Improve local blood circulation and promote the recovery of synovitis through techniques like massage and heat therapy. You can apply a warm towel or use a hot water bag locally.
