# Supplementary figures and images for: Quality and Dependability of ChatGPT and DingXiangYuan Forums for Remote Orthopedic Consultations: Comparative Analysis
Source: J Med Internet Res. 2024 Mar 14;26:e50882. doi: 10.2196/50882 (PMC10979330; doi:10.2196/50882)

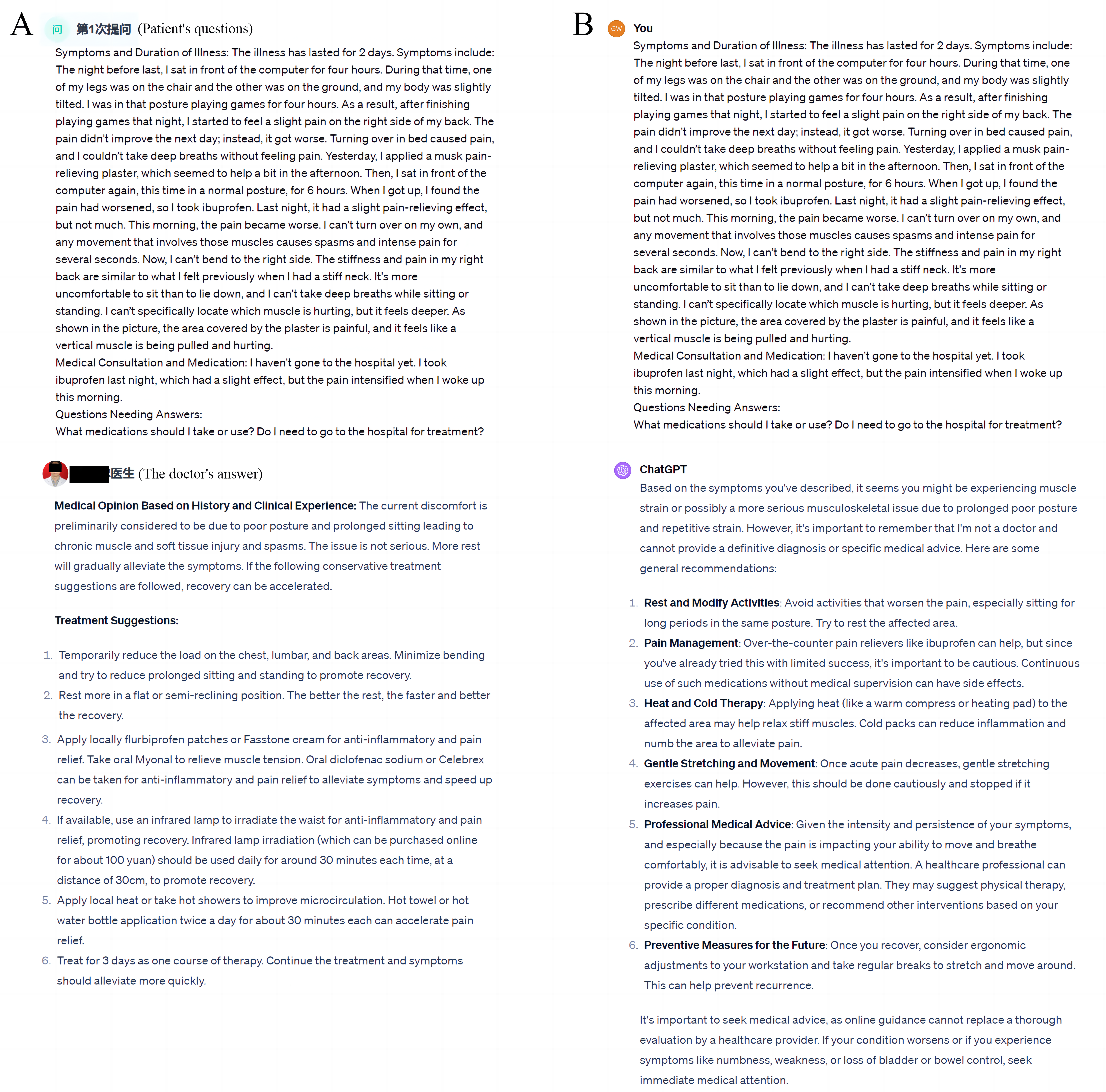

Supplement: Multimedia Appendix 3 [file jmir_v26i1e50882_app3.zip › Figure 1 Hd version.png]
