# Supplementary material for: Quality and Dependability of ChatGPT and DingXiangYuan Forums for Remote Orthopedic Consultations: Comparative Analysis
Source: J Med Internet Res. 2024 Mar 14;26:e50882. doi: 10.2196/50882 (PMC10979330; doi:10.2196/50882)
Supplement: Multimedia Appendix 4 [file jmir_v26i1e50882_app4.docx]

1.

The symptoms you described appear to be indicative of a condition called Rheumatoid Arthritis (RA), which is an autoimmune disease primarily affecting the joints and can result in pain, swelling, and stiffness. Rheumatoid arthritis symptoms are often more severe in colder environments or upon waking in the morning. However, please note that this is just a possibility, and a definitive diagnosis should be made by a doctor through a physical examination and relevant tests.

If your symptoms are due to Rheumatoid Arthritis, treatment may include:

Medications: This can include nonsteroidal anti-inflammatory drugs (NSAIDs), corticosteroids, disease-modifying antirheumatic drugs (DMARDs), and biologics. These medications can help alleviate pain and inflammation, and prevent or slow joint damage.

Physical therapy: Physical therapists can provide guidance on how to protect your joints and recommend exercises to help maintain joint flexibility.

Surgery: In cases of severe joint damage, surgery may be necessary to repair or replace the affected joint.

Lifestyle changes: A healthy diet, appropriate exercise, and adequate rest can all help in managing symptoms.

Regarding your mention of a Vitamin D3 deficiency, Vitamin D is crucial for bone health and has also been found to be associated with immune function. You can increase your Vitamin D levels through dietary sources (such as fatty fish, milk, eggs, etc.), exposure to sunlight (the skin can produce Vitamin D from UVB rays in sunlight), and by taking Vitamin D supplements or supplements.

However, despite providing these possible answers, it's essential to seek advice from a medical professional. A doctor will conduct a comprehensive assessment of your symptoms and may require further tests to establish a diagnosis. Please seek medical attention as soon as possible and provide them with detailed information about your symptoms and your entire medical history.

2.

Your symptoms may be due to overuse of your hands, especially from repetitive tasks such as prolonged typing or smartphone use, which can lead to a condition called Repetitive Strain Injury (RSI). This is a common work-related condition, especially among those who spend extended periods using computers. RSI can result in pain or discomfort in muscles, nerves, tendons, and other soft tissues.

Another possible condition is tenosynovitis, which is inflammation of tendons caused by prolonged repetitive movements. Tenosynovitis typically occurs in the wrists and fingers and can lead to pain, swelling, and limited mobility.

However, only a doctor can provide an accurate diagnosis. I recommend that you see a doctor as soon as possible and describe your symptoms and work situation in detail. They may perform diagnostic tests such as X-rays, ultrasound, or MRI to help determine the diagnosis.

If indeed diagnosed with RSI or tenosynovitis, the following advice may be helpful:

Rest and Avoid Repetitive Movements: This is the first step in treating RSI. You may need to change your work habits to avoid repetitive hand movements.

Cold or Hot Compresses: Cold compresses can help alleviate pain and swelling, while hot compresses can relax tense muscles.

Over-the-Counter Medications: Ibuprofen or nonsteroidal anti-inflammatory drugs (NSAIDs) can help relieve pain and swelling.

Physical Therapy: If symptoms persist, you may need to see a physical therapist. They can teach you stretching and strengthening exercises for your muscles and tendons, as well as how to modify your daily activities to prevent further injury.

Perform Hand Stretches: Gentle stretching exercises can improve the flexibility of your muscles and tendons, preventing further injury.

Improve Your Work Environment: Adjust your workstation's equipment and furniture to ensure they are ergonomically suited to your body and work style. For example, you can use ergonomic keyboards and mice to reduce pressure on your hands.

3.

Based on the symptoms you've described, it's possible that your condition may be due to poor posture and prolonged maintenance of the same position, leading to a strain or muscle spasm in your back. Prolonged improper sitting posture can cause excessive muscle tension, resulting in muscle pain, and in severe cases, it may compress nerves, exacerbating the pain.

Regarding your issue, here are some suggestions, but please remember that these cannot substitute for professional medical advice.

Medications: You can continue taking over-the-counter non-steroidal anti-inflammatory drugs (NSAIDs) like ibuprofen to help alleviate pain and inflammation. If there is no significant improvement in pain or if the pain worsens after taking medication, you should seek medical attention. Additionally, some muscle relaxants like cyclobenzaprine may help relieve muscle spasms, but these typically require a prescription from a doctor.

Hot and Cold Compresses: In the initial 48-72 hours, you can use cold compresses (such as an ice pack) to reduce pain and swelling, followed by applying heat (e.g., a heating pad or warm towel) to relax the muscles.

Rest and Posture Adjustment: You should reduce prolonged sitting, especially in fixed positions. Take breaks periodically to stand up, move around, and perform gentle stretching exercises to relax your muscles.

Physical Therapy: After a medical evaluation by a doctor, you may be recommended for physical therapy. This could include massages, traction, and other treatments aimed at alleviating muscle tension and pain.

In all these cases, it's crucial to seek professional medical help. Given that your pain has significantly impacted your daily life, it's advisable to consult a healthcare professional promptly. The doctor may suggest undergoing tests such as X-rays or MRI to rule out potential serious issues, such as spinal problems. Only a doctor can provide the correct diagnosis and recommend a treatment plan tailored to your condition.

4.

Your symptoms suggest that you may have sustained some injury to your knee during the fall. This could include soft tissue damage such as muscle or ligament strains, or it could involve a fracture. However, there is a question here: if it were a fracture, the pain is typically more severe than what you've described and may be accompanied by an inability to bear weight or walk. The protrusion and swelling in your knee could be due to bleeding or fluid accumulation, which is the body's response to injury.

Based on your symptoms, you should seek medical attention as soon as possible, ideally with an X-ray examination to determine if there is a fracture. If there is no fracture, your doctor may recommend physical therapy to help restore normal function to your knee joint.

Before seeing a doctor, here are some things you can do:

Continue applying ice. This can help alleviate pain and reduce swelling.

Rest and elevate the injured leg. Try to avoid putting pressure on the injured knee.

Continue using a knee brace, but avoid it being too tight, as this may further increase swelling.

If possible, try gentle knee joint movements. This can help reduce stiffness and promote recovery.

5.

Your medical history and symptoms could be indicative of a problem with your lumbar spine, which could potentially involve issues like lumbar spondylolisthesis, lumbar disc protrusion, or other forms of lower back injuries. Of course, it could also be a muscle strain or ligament sprain. The specific condition would need to be determined through clinical examination by a doctor and possible imaging studies like X-rays, CT scans, or MRIs.

Lumbar spondylolisthesis refers to an abnormal movement of one vertebra of the spine relative to the one below it. Lumbar disc protrusion, on the other hand, involves the protrusion of the soft cushioning disc (intervertebral disc) between lumbar vertebrae, which can compress nerve roots and commonly presents symptoms like lower back pain and sciatica (radiating pain down the back of the leg).

Regardless of the specific condition, your symptoms require evaluation and treatment by a medical professional, especially if the pain is affecting your daily activities such as sitting, walking, or using the restroom. Treatment options may include physical therapy, medication, or other therapies. In some severe cases, surgery may be necessary.

Before receiving professional medical help, you can try to alleviate your symptoms by resting in bed, avoiding activities that may worsen the pain such as bending, twisting, or heavy lifting, and using over-the-counter medications (such as ibuprofen or other non-steroidal anti-inflammatory drugs) to help with pain and inflammation. However, please be aware that these measures provide temporary relief and do not address the underlying issue, so it's important to seek medical attention as soon as possible.

6.

Your symptoms may indeed be related to tendonitis, especially considering your previous history of such a diagnosis. Tendonitis is often caused by overuse or injury. The goal of treatment is to alleviate pain and inflammation and gradually restore normal activity.

Regarding medication: Since your medication is running out, and your symptoms have not completely disappeared, it's advisable to consult a doctor again. The doctor may continue prescribing similar medications or may suggest you try some other treatment options. Physical therapy or other non-pharmacological therapies may also be considered.

Regarding the recovery time: The recovery time for tendonitis varies from person to person and may take several weeks to several months. If the symptoms persist or worsen, it's crucial to see a doctor again.

Regarding exercise: In general, you should avoid excessive use or straining of your knee, as it can exacerbate pain or prolong the recovery time. However, moderate exercise and gradually increasing intensity can be beneficial for recovery. A physical therapist can help you develop a suitable exercise plan to strengthen the muscles around your knee, providing better support and reducing the risk of future injuries.

In summary, if your symptoms haven't significantly improved or if you continue to experience noticeable symptoms after a period of using medication, it's important to see a doctor again for further evaluation and treatment advice.

7.

Your symptoms may be due to overuse of muscles, muscle tension, or potential injury. Increasing your abdominal wheel training may have put additional stress on your lower back muscles, which could explain the pain in your left side waist and hip. Additionally, the wrist issue may be related to overuse of hand and forearm muscles, especially during strenuous exercises like abdominal wheel training.

The primary goal of treatment should be to alleviate pain and inflammation and attempt to restore normal activity. Here are some possible treatment strategies:

Rest: Rest is crucial for muscles that have been overused or injured. You may need to temporarily reduce or modify your exercise activities to allow your body to recover.

Cold and Heat Therapy: In the first few days when pain is most severe, cold therapy can help reduce pain and inflammation. Afterward, heat therapy can help relax tense muscles and increase blood flow.

Gentle Activity and Mild Stretching Exercises: As pain subsides, gentle activity and mild stretching exercises can help increase your range of motion and prevent muscle stiffness.

Over-the-Counter Medications: Over-the-counter pain relievers or non-steroidal anti-inflammatory drugs (NSAIDs) like ibuprofen can help alleviate pain and inflammation.

Regarding your exercise concerns, as long as your symptoms permit, you can gradually resume your exercise routine. However, you need to listen to your body and stop if you feel pain. Gradually increasing the intensity of your activities and avoiding sudden increases can help prevent worsening pain. Lastly, you may need to reassess your training plan and techniques to ensure your body responds to training in a healthy manner.

If your symptoms persist or worsen, it's advisable to see a doctor, as you may need further evaluation and treatment. Additionally, considering your status as a marathon runner, you may benefit from specific advice and treatment from a physical therapist or sports medicine specialist.

8.

The condition you describe appears to be a protruding rib or, more specifically, a protruding xiphoid process. This condition is not uncommon and can sometimes be congenital or may be due to other factors such as posture or changes in body weight. In most cases, if a protruding xiphoid process does not cause pain or other symptoms, special treatment is not necessary. However, if you feel discomfort or experience pain, it may be advisable to seek medical advice.

You may need to consult a doctor for a comprehensive physical examination to rule out other potential causes. This may include a physical examination and may also involve imaging tests such as X-rays or ultrasound. In some cases, if the symptoms caused by the protruding xiphoid process are severe, surgery may be recommended by a doctor, although this is very rare.

If you do not have other symptoms such as pain, difficulty breathing, or swallowing difficulties, and your doctor has confirmed that there are no other serious underlying causes for your condition, you may not require specific treatment. In some cases, modifying daily habits, such as avoiding certain movements that may exacerbate symptoms, or undergoing physical therapy that can improve symptoms, may be helpful.

In conclusion, if your symptoms persist or worsen, it is important to seek medical attention.

9.

For your situation, it's important to clarify that I cannot replace a real doctor for diagnosis and prescriptions; I can only offer some suggestions. Your symptoms sound like sciatica, which can be caused by a herniated disc in the lumbar spine. In many cases, the pain may naturally subside over several days or weeks. However, if the pain is very severe, it's essential to see a doctor immediately, especially if you experience any of the following:

Numbness in the lower body, including the anus or genitals.

Loss of bowel or bladder control.

Weakness in the leg or foot muscles.

Unexplained recent weight loss.

While waiting to see a doctor, you can try some measures to relieve the pain. Over-the-counter medications like ibuprofen or aspirin can help reduce pain and inflammation. Please use them according to the package instructions and avoid overdosing. Besides medication, you can also try gentle heat or cold therapy or perform some mild stretching exercises to alleviate pressure.

Once you're able to stand up, you should make an appointment to see a doctor as soon as possible. They may recommend physical therapy or prescribe medications to manage pain and inflammation. In some cases, surgery may be necessary to address the underlying issue causing your pain.

Additionally, since you have a history of herniated discs, you should discuss with your doctor how to prevent such problems in the future. This may involve lifestyle changes like paying more attention to your posture, avoiding heavy lifting, and doing exercises to strengthen your core muscles.

In conclusion, while I've provided some general advice, I strongly recommend contacting healthcare professionals promptly for specialized medical advice and treatment.

10.

Your symptoms could be caused by various factors, including but not limited to overuse, arthritis, sprains, ligament strains, or other joint issues. Based on your description, especially starting running recently without warming up, it's possible that it could be due to overuse or a sprain. Overuse and sprains can lead to inflammation in the muscles, ligaments, or other structures around the ankle, resulting in pain.

Methods to alleviate ankle pain primarily include:

Rest: Temporarily cease or reduce the use of the injured ankle, especially activities that involve weight-bearing on the ankle, such as running.

Ice: Applying an ice pack to the painful ankle for 15-20 minutes at a time can help reduce inflammation and pain.

Compression and Elevation: Using an elastic bandage to wrap the injured ankle can help reduce swelling, and elevating the ankle above heart level whenever possible can assist in minimizing the accumulation of blood and fluid at the injury site.

Medications: Over-the-counter medications like ibuprofen or aspirin can help reduce pain and inflammation. Please use them according to the instructions on the medication packaging and avoid overdosing.

You should also consider seeking medical help, especially if your symptoms do not improve or worsen after rest and the self-care measures mentioned above. A doctor can help identify the cause of the pain and provide appropriate treatment recommendations. Additionally, if you plan to continue running, it may be beneficial to seek guidance from a fitness trainer or physical therapist who can instruct you on proper warm-up techniques and how to safely increase your exercise intensity to prevent future injuries.

11.

Your mother's chronic pain lasting for 8 years is a very long duration, and it appears to significantly affect her daily life. Pain can have various underlying causes, including diseases, overuse, muscle tension, or other health issues. The first and most crucial step is to identify the root cause of the pain so that the most effective treatment approach can be determined.

For long-term pain, a multimodal treatment approach may be necessary. This may include:

Medication Therapy: Over-the-counter medications like ibuprofen and aspirin, as well as prescription medications such as pain relievers and anti-inflammatories, may help alleviate pain. Your doctor may recommend specific medications based on your mother's symptoms and the severity of her pain.

Physical Therapy: Physical therapists can provide pain relief strategies, which may include heat/cold therapy, electrical stimulation therapy, as well as specific stretching and strengthening exercises.

Exercise and Activity: Some types of pain can be alleviated through regular low-impact exercises, such as walking, swimming, or yoga. This can help strengthen muscles, reduce pain, and improve flexibility and balance.

Alternative Therapies: Acupuncture, massage therapy, and yoga, among others, have been shown to provide pain relief for some individuals.

Psychological Therapy: If the pain is impacting your mother's mental health, counseling or consultation with a mental health specialist may be helpful.

If your mother's pain persists and is severe, she should seek medical help to determine the underlying cause of the pain and develop a personalized treatment plan. Treating chronic pain may require some time and patience, but by working with a doctor, your mother can find a way to manage her pain and improve her quality of life.

12.

Hello, while I'm not a doctor, I can offer some possible explanations.

The symptoms you describe could be due to overuse or muscle tension. Prolonged hours working at the computer, especially in incorrect posture, can lead to tension and pain in the arms, shoulders, and neck. Additionally, gym workouts may also contribute to elbow pain, particularly if you are engaging in weight training or not warming up and cooling down properly.

Here are a few suggestions that might help you:

Rest: Temporarily avoid repetitive or excessive use of the affected elbow. Give your muscles time to recover.

Hot and Cold Therapy: Apply cold packs or warm compresses to the painful area. Cold packs can help reduce swelling and pain, while warm compresses can help relax muscles and improve blood flow.

Stretching and Strengthening Exercises: Some simple elbow stretching and strengthening exercises may help improve elbow pain.

Proper Ergonomics: Ensure that your workspace and chair are set up correctly, and that your arms and wrists are in the proper position.

If your pain persists, worsens, or is accompanied by swelling, redness, numbness, or a tingling sensation, you should seek medical attention promptly. Further examination may be necessary to determine the cause of the pain and identify the most suitable treatment approach.

13.

Hello, I will do my best to answer your questions:

"Small cystic lesion" in medical imaging typically refers to a small fluid-filled cyst. However, your doctor saying it's a "presentation" might suggest that it is an incidental finding and not the cause of your pain.

Small cystic lesions can result from various factors, including early injury, inflammation, degenerative changes, or normal anatomical variations. For your specific case, your doctor may provide a more specific explanation based on your medical history and examination results.

Small cystic lesions may resolve on their own over time or may persist. The likelihood of recurrence depends on the underlying cause, such as early injury or inflammation.

Regarding the source of your pain, I cannot provide a specific answer as it would require knowledge of your specific medical history and examination findings. Possible causes could include inflammation, injury, or overuse. Your doctor may offer a more specific explanation.

"T1WI shows low signal, and PD fat-suppressed shows high signal" refers to technical details of MRI imaging. T1WI (T1-weighted imaging) and PD (proton density) fat-suppressed are two different MRI scanning techniques with different sensitivities to different types of tissues. The small cystic lesion appearing as low signal on T1WI and high signal on PD fat-suppressed may indicate it is a fluid-filled cyst.

Please remember that the answers provided are based on general knowledge and not a substitute for personalized medical advice based on your specific medical history. If you have further questions about this issue or if your symptoms change or worsen, you should contact your doctor promptly.

14.

Hello, based on your description, your inner knee pain may be due to overuse or exercise-related injury. The knee is a complex joint with many structures that can be injured during physical activity, including ligaments, tendons, cartilage, or meniscus.

However, please note that long-distance or high-intensity running, jumping, or twisting movements, especially without adequate warm-up or preparation, can all potentially lead to knee injuries. In addition, improper footwear or exercise technique can also contribute to knee pain.

It's important to address your concern about osteosarcoma. While osteosarcoma can occur in young people, it is a rare disease, and typical symptoms include persistent pain (including at night), swelling or lumps, and systemic symptoms like unexplained weight loss or fever. This does not seem to align with the symptoms you have described, so you don't need to be overly worried about it.

However, regardless of the cause, experiencing pain for a month warrants attention. It is advisable to seek medical attention promptly for further evaluation to determine the cause of the pain and receive appropriate treatment. During this time, avoid overusing or engaging in vigorous activities that stress your knee, and you can try using ice packs to relieve pain and inflammation.

Please remember that these are general recommendations based on the symptoms you have described and do not substitute for in-person medical consultation. If the pain persists or if you experience any new symptoms, you should seek medical attention promptly.

15.

Based on your description, your mother may have a herniated disc or another related condition that could be causing irritation or compression of nerve roots, leading to pain or numbness.

For treatment, in the case of a lumbar disc herniation, medication is primarily aimed at relieving pain and reducing inflammation. Medications may include non-steroidal anti-inflammatory drugs (NSAIDs) such as ibuprofen and muscle relaxants. If medications and physical therapy are ineffective or if the condition significantly affects the quality of life, surgical treatment may be considered.

Regarding exercise therapy, a specialized physical therapist can create an appropriate exercise program based on the patient's specific condition. Gentle exercises such as core strengthening, yoga, or tai chi may also be helpful. However, it's essential to consult a doctor or physical therapist before engaging in any exercises, as improper exercise could potentially worsen the symptoms.

In terms of diagnostic tests, if the MRI shows a disc herniation and the symptoms are consistent with it, further testing may not be necessary. However, if the symptoms persist, worsen, or new symptoms develop, further evaluation may be required.

Overall, given the complexity and duration of your mother's condition, it is advisable to seek consultation with a specialized neurologist or orthopedic surgeon as soon as possible. They can provide a more personalized treatment plan for your mother.

During this time, it is recommended to avoid activities that involve repeated bending, heavy lifting, or vigorous exercise in the affected area to prevent worsening of the condition.

The information provided here is based on the symptoms and circumstances you described and should not replace the diagnosis and advice of a healthcare professional. If the symptoms persist or worsen, seeking medical attention is essential.

16.

Your symptoms could be due to wrist joint overuse or inflammation, such as tenosynovitis, arthritis, or other related conditions. However, please note that this is just a preliminary assessment based on the symptoms you described, and a specific diagnosis requires evaluation and examination by a healthcare professional.

Medical advice: I recommend that you seek medical attention from an orthopedic specialist or a sports medicine physician as soon as possible. The doctor may conduct a physical examination, consider your symptoms, or may perform imaging studies such as X-rays or an MRI to confirm the diagnosis.

Treatment advice:

For many cases of wrist pain, the primary treatment approach is rest and avoiding activities that exacerbate the pain. Wearing a wrist brace or bandage may be necessary to protect the wrist. Over-the-counter non-steroidal anti-inflammatory drugs (NSAIDs) such as ibuprofen or aspirin can help alleviate pain and inflammation. However, it's important to follow the doctor's recommendations and be aware of potential side effects of the medication. Physical therapy may also be beneficial, including hot and cold therapy, massage, and specific wrist and hand exercises to improve flexibility and strength. If conservative treatments mentioned above are ineffective, the doctor may consider corticosteroid injections or, in severe cases, surgery. The recovery time will depend on the specific cause of the pain and the effectiveness of the treatment, ranging from weeks to months.

Once again, it's important to emphasize that the above recommendations are based on the symptoms you described and do not substitute for a doctor's diagnosis and treatment. If your symptoms persist or worsen, it is advisable to seek medical attention promptly.

17.

Your situation appears to involve pain and swelling that may be caused by an infection or injury. If you have already been taking antibiotics and receiving intravenous fluids (IV), but your symptoms haven't improved, it's crucial to seek medical help promptly. Further tests may be necessary to determine the cause of the pain, which could include imaging studies such as X-rays or ultrasound.

Additionally, your doctor may need to adjust the type or dosage of antibiotics. Ensure that you complete your full course of antibiotics as prescribed by your doctor, even if your symptoms have improved, to ensure that the infection has been completely cleared.

While waiting for your appointment, try to rest the injured foot as much as possible, and you can use cold compresses to reduce swelling and pain. If possible, elevating the injured foot can help reduce swelling.

Please note that if you experience severe pain, high fever, chills, rapidly spreading swelling, changes in skin color (such as turning red, black, or purple), or other concerning symptoms, you should seek immediate medical attention as these could be signs of a rapidly progressing infection.

18.

Your symptoms could be related to various conditions, including:

Arthritis: Joint pain and stiffness can be caused by inflammation, diseases, or age-related changes. For example, osteoarthritis or rheumatoid arthritis may lead to such symptoms.

Fatigue or overuse: Prolonged use of your hands or toes may result in pain and stiffness.

Nerve compression: Certain conditions such as cervical spondylosis or tenosynovitis could cause pain in the fingers and toes.

These are potential causes and cannot definitively identify your specific condition. If the pain and stiffness persist or worsen, you should seek medical attention as soon as possible. A doctor may perform physical examinations and, if necessary, order blood tests or imaging studies to help determine the cause.

Before seeking medical care, you can try some measures that may help alleviate the pain, such as adequate rest, avoiding overuse of your fingers and toes, and using warm compresses. However, these measures can only provide temporary relief and do not treat the underlying condition.

If the pain worsens or is accompanied by other symptoms such as fever, joint swelling, skin redness, or joint deformities, you should seek medical attention immediately.

19.

Swelling and pain in the ankle can have many possible causes, such as sprains, overuse, arthritis, and more. Changes in your walking posture after an injury can indeed lead to increased pressure and pain in other areas, but it cannot be confirmed whether this is the cause of your ankle pain.

In the absence of immediate medical attention, you can try some self-care measures:

Rest: Avoid using the painful ankle to give it a chance to rest.

Ice: Apply ice for 15-20 minutes at a time, every 2-3 hours, to help reduce pain and swelling.

Compression: Use an elastic bandage to wrap the ankle, but be careful not to wrap it too tightly to avoid affecting blood circulation.

Elevation: When resting, try to elevate the ankle above the level of your heart to reduce swelling.

The Fluibiprofen gel patch you are using is a non-steroidal anti-inflammatory drug (NSAID) that can help relieve pain and swelling. Please follow the instructions on the package for proper use.

If your symptoms do not improve within a few days or if you experience other severe symptoms (such as high fever, chills, redness of the skin, worsening pain, etc.), as soon as the lockdown is lifted or if it becomes possible, please seek medical attention immediately.

20.

Hello, for your condition, there are several possibilities to consider:

Gouty arthritis: Although gout most commonly affects the big toe, it can also involve other joints, including the elbow. You mentioned that your uric acid is controlled at around 300, which generally should not trigger a gout attack. However, everyone's tolerance for uric acid levels may vary. Gout typically causes acute pain, swelling, fever, and joint stiffness, which align with your symptoms.

Bursitis: There are fluid-filled sacs called bursae around our joints that help reduce friction between tendons and bones. Inflammation of these bursae can lead to bursitis. Bursitis typically causes localized pain and swelling.

Other types of arthritis: Conditions like osteoarthritis or rheumatoid arthritis can also cause joint pain but usually come with other symptoms like morning stiffness or pain in other joints.

Tendonitis or muscle strain: These typically cause pain during activity or exertion and may improve with rest.

The sustained-release sodium diclofenac tablets you are taking are a type of non-steroidal anti-inflammatory drug (NSAID) that can help alleviate pain and inflammation.

Self-management at home includes:

Continue taking the sustained-release sodium diclofenac tablets as directed by your doctor, avoiding exceeding the recommended dose.

Apply cold compresses to the elbow for 15-20 minutes several times a day to help reduce pain and swelling.

Avoid using the painful elbow and give it some rest.

These measures can help alleviate your symptoms, but if your symptoms do not improve within a few days or if the pain worsens, your elbow becomes warm, or you experience other new symptoms such as general fatigue or fever, it is advisable to contact your doctor promptly or seek medical attention.

21.

Firstly, I need to remind you that I am an artificial intelligence assistant, and I can provide general advice but cannot replace a doctor's professional medical opinion. There could be multiple potential causes for your symptoms, including but not limited to pain syndromes, sprains, arthritis, or gout.

Acute arthritis is one possibility, but typically, this condition would be accompanied by other symptoms such as joint swelling, redness, tenderness, warmth, or limited mobility. While soaking in cold water may cause some discomfort or pain, it generally wouldn't directly lead to acute arthritis.

Gout is a specific type of arthritis that often initially affects the big toe joint and presents with severe pain, swelling, warmth, and tenderness. It is caused by elevated levels of uric acid, which crystallize in the joints.

If your pain persists, is severe, or is accompanied by other symptoms like swelling, redness, or warmth, you should seek medical attention promptly. While waiting to see a doctor, trying ice packs on the painful area and avoiding overuse of the joint may provide some relief. Additionally, it's advisable to consult with a healthcare professional before using any medications or pain relief methods.

22.

The symptoms you describe could be a manifestation of heel pain, and the specific causes may include Achilles tendonitis, plantar fasciitis, or other foot injuries. However, as I cannot conduct a physical examination or other diagnostic procedures, this is just a possible explanation based on the symptoms you've described. If your pain persists or worsens, or if you experience other symptoms such as heat, redness, swelling, or impaired mobility, you should seek medical assistance as soon as possible.

In this situation, you can try the following methods to alleviate the pain:

Rest: Allow the injured foot to rest adequately and avoid overuse.

Cold Compress: Apply a cold pack (a mixture of ice and water, wrapped in a towel to prevent frostbite) to the painful area for 15-20 minutes at a time, every 2-3 hours.

Elevation: Whenever possible, try to elevate the injured foot, as it can help reduce swelling.

Over-the-Counter Pain Relievers: For some people, over-the-counter non-steroidal anti-inflammatory drugs (such as ibuprofen) may help relieve pain and swelling. However, these medications are not suitable for everyone, especially those with stomach, liver, kidney issues, or those taking other medications. Therefore, you should consult a healthcare professional before starting any new medication.

Rubbing alcohol may provide some temporary relief, but it does not address the underlying issue. Please remember that these are preliminary recommendations and cannot replace the professional medical advice of a doctor. If the pain persists or worsens, or if other symptoms develop, you should seek medical assistance promptly.

23.

Your symptoms could be caused by various reasons, including but not limited to a knee sprain, tendon strain, knee arthritis, or gout. While seafood consumption can trigger gout in some individuals, the likelihood is relatively low if you haven't had a history of elevated uric acid levels.

Here are some temporary measures you can try to relieve the pain:

Rest and avoid overusing your knee joint. You may need to reduce or avoid all activities that could potentially exacerbate knee joint pain.

Apply a cold compress to your knee joint. You can use an ice pack (or a mixture of ice and water wrapped in a towel to prevent frostbite) on your knee for about 15 to 20 minutes at a time.

Take over-the-counter pain relievers. Medications like ibuprofen or aspirin may help alleviate pain and swelling. However, remember that these medications are not suitable for everyone, especially those with heart conditions, high blood pressure, kidney issues, or stomach ulcers. They should consult a doctor before using these medications.

However, these are just temporary relief measures. If your pain persists, worsens, or if other symptoms develop, you should seek medical assistance promptly. It's essential to identify the underlying cause of the pain so that appropriate treatment can be provided. Your doctor may recommend tests such as X-rays or MRI to help diagnose your condition.

24.

Osteoporosis is a common bone disorder, especially in older individuals. It is characterized by reduced bone density, leading to fragile and easily fractured bones. The primary treatment goal for osteoporosis is to prevent fractures, as fractures can result in pain, reduced mobility, and a decreased quality of life.

Your grandmother's symptoms (excessive sweating, fever, pain, sleep problems) may be related to osteoporosis, but they could also be caused by other health issues. Therefore, it's best to seek medical advice to determine the underlying cause of these symptoms.

Treatment for osteoporosis typically includes:

Dietary and lifestyle adjustments: Ensuring an adequate intake of calcium and vitamin D is crucial for bone health. Calcium supplementation and exposure to sunlight are good methods, but calcium-rich foods such as dairy products and vitamin D-rich foods like fish and eggs can also be included in the diet. Additionally, regular moderate physical activity, like walking, can help maintain bone density and strengthen muscles, reducing the risk of fractures.

Medication therapy: There are several medications available that can help prevent fractures caused by osteoporosis, including bisphosphonates, selective estrogen receptor modulators (SERMs), calcitonin, parathyroid hormone, and denosumab. The choice and use of these medications should be guided by a healthcare provider, as each has its potential side effects and contraindications.

Pain management: If osteoporosis leads to pain, pain management medications such as over-the-counter non-steroidal anti-inflammatory drugs (e.g., ibuprofen) may be necessary. Prescription medications may be required for more severe pain.

Please remember that these are potential treatment options, and the choice of treatment should be made and guided by a healthcare provider based on your grandmother's specific circumstances. Additionally, if her symptoms persist, worsen, or new symptoms develop, seeking help from a doctor as soon as possible is important.

25.

Your symptoms sound like they could be due to either foot calluses (often forming as hard skin) or plantar warts. These conditions are typically a result of excessive pressure or friction on the feet and commonly occur on the soles, heels, or toes.

Treatment options may include:

Self-care: Soak your feet in warm water and then gently remove the calluses with a pumice stone or foot file. Exercise caution to avoid cutting healthy skin. Using lotions containing urea can help soften the hard skin.

Patches: Drugstores often sell patches containing salicylic acid for treating calluses. These patches can help soften the hard skin, making it easier to remove. However, if you have diabetes or circulatory problems, consult a doctor before using these products.

Insoles: Using insoles designed to reduce foot pressure may help prevent the formation of calluses.

Change of shoes: Wearing ill-fitting shoes can increase pressure on the feet, leading to callus formation. Choosing well-fitting, comfortable, and adequately spacious shoes may help address this issue.

If the above treatment methods are ineffective or if the pain persists, worsens, or if you notice redness, swelling, or fever, seek medical help promptly, as these could be signs of an infection. A doctor may recommend other treatment options, such as stronger medications or physical therapy, or they may perform surgical removal of calluses.

It's important to note that if you have diabetes or other conditions that may affect foot circulation, you should seek medical help promptly, as these conditions can impact wound healing and increase the risk of infection.

26.

Your mom's symptoms could be due to a ganglion cyst or a tenosynovial cyst, both of which are common benign tumors that typically contain fluid and feel soft to the touch. Their size may vary over time.

Ganglion cyst: A ganglion is a small sac located near joints and tendons, filled with synovial fluid that reduces friction and aids in smooth joint movement. When a ganglion becomes inflamed or injured, synovial fluid may accumulate and form a cyst.

Tenosynovial cyst: A tendon sheath is a thin membrane that surrounds tendons and contains lubricating fluid, facilitating the gliding of tendons. In cases of inflammation or injury to the tendon sheath, a tenosynovial cyst can develop.

In general, if these cysts are painless and do not affect function, they may not require specific treatment and can be observed for a period to see if they naturally resolve. Avoiding excessive use of the wrist can help reduce pain and the size of the cyst. If there is pain or functional impairment, wrist splints may be considered to limit wrist movement, or over-the-counter nonsteroidal anti-inflammatory drugs (such as ibuprofen) can be taken to alleviate pain.

However, if the cyst persists, continues to enlarge, or causes wrist dysfunction, it may be advisable to consult a doctor. The doctor may opt for aspiration (fluid removal) or surgical excision of the cyst.

It's important to note that while these possibilities are more likely based on your description of the symptoms, they are preliminary assessments and do not constitute a definitive diagnosis. If the cyst persists, enlarges, causes pain, or presents with new symptoms, you should promptly assist your mom in contacting a healthcare professional for a detailed examination and an accurate diagnosis and treatment.

27.

Your symptoms could be caused by various factors, such as arthritis (such as rheumatoid arthritis or osteoarthritis), repetitive strain (due to repetitive tasks), or even weather changes.

Here are some home remedies that you can try:

Warm Therapy: Applying heat to the affected hand can help relieve pain and stimulate blood circulation. You can use a hot water bottle, an electric blanket, or simply soak your hand in warm water.

Over-the-Counter Medications: Non-prescription non-steroidal anti-inflammatory drugs (NSAIDs), such as ibuprofen, may help alleviate pain and reduce inflammation. Follow the instructions on the medication, and consult a pharmacist or doctor if you have any concerns.

Rest and Gentle Exercise: Rest appropriately and avoid overusing the affected joints. Additionally, performing gentle exercises, such as finger joint flexion and extension, can help maintain joint flexibility and strength.

Use Hand Cream: During cold and dry seasons, the skin on your hands can become dry and cracked, potentially causing pain. Using hand cream can help protect your skin and prevent dryness.

These are all temporary relief measures. If the pain continues unabated or if new symptoms emerge, such as redness, increased warmth, or restricted movement, you should seek medical help promptly, as this could indicate a more serious underlying condition. More severe conditions may require different forms of treatment, including medications or even physical therapy or surgery. Additionally, to determine the cause of the pain, your doctor may conduct various tests, such as blood tests or imaging studies.

28.

Your grandma's symptoms could be caused by various factors, including but not limited to knee arthritis (such as osteoarthritis or rheumatoid arthritis), soft tissue injuries (such as tendon or ligament injuries), bursitis, cysts, or other knee-related conditions.

Here are some possible treatment options, but they should be used under the guidance of a doctor:

Medication: For knee arthritis, doctors typically recommend trying non-prescription medications first, such as non-steroidal anti-inflammatory drugs (NSAIDs) or acetaminophen to alleviate pain and inflammation.

Physical Therapy: Physical therapy may help improve joint flexibility and strength, reducing pain. Physical therapists may recommend exercises that can be done at home.

Surgery: For severe arthritis, if non-surgical treatments are ineffective, surgery such as joint replacement may be necessary.

Self-Management: Maintaining a reasonable body weight and engaging in low-impact exercises (such as swimming or cycling) can help reduce stress on the knee joint and alleviate pain.

Please have your grandma see a doctor as soon as possible. The doctor will conduct a comprehensive assessment, may perform some tests (such as X-rays or MRI), and will recommend the most suitable treatment plan based on your grandma's health condition and symptoms. Do not initiate any treatment on your own without the advice of a doctor.

29.

Based on your description, the swelling in the child's knee could be due to various reasons, such as mild sports-related injuries (like strains or sprains), overuse, or bursitis, among others. Trampolining is an activity that could potentially subject the joints to strain or twisting, which might be the cause of knee swelling.

In this situation, one common method for treatment and pain relief is the RICE principle:

Rest: Avoid activity and walking to prevent further injury.

Ice: Apply an ice pack to the swollen area for about 15-20 minutes at a time, 3-4 times a day.

Compression: Use an elastic bandage to wrap the knee to reduce swelling.

Elevation: When resting, try to elevate the knee, placing it on a pillow or soft cushion so that it's higher than the level of the heart, which can help reduce swelling.

If the swelling in the knee persists or worsens (e.g., if there is pain, redness, warmth, limited mobility, etc.), it's important to seek medical attention promptly as it may indicate a more serious injury.

It's worth noting that even if the child doesn't experience pain, if the knee swelling doesn't significantly improve within 48 hours, it's advisable to consult a doctor. In such cases, the doctor may recommend further examinations, such as X-rays or ultrasound, to understand the specific cause of the swelling and determine the most appropriate treatment plan.

30.

Your symptoms could be caused by overuse, prolonged standing or walking, and changes in environmental conditions. The pain and swelling in your heel and knee could be due to conditions such as Achilles tendonitis, plantar fasciitis, or osteoarthritis. Here are some common symptoms and possible reasons:

Achilles Tendonitis: This is inflammation of the Achilles tendon (the large tendon that connects the heel to the calf muscles) caused by overuse or injury. Symptoms include pain and stiffness in the heel or the back of the calf.

Plantar Fasciitis: The plantar fascia is a thick tissue that connects the heel bone to the toes. When this tissue is overstretched or inflamed, it can cause pain in the arch of the foot.

Osteoarthritis: This is a common form of arthritis that can lead to joint pain and stiffness, often affecting the knees.

If the pain affects your daily activities or persists, you should seek medical attention. Your doctor may need to perform tests such as X-rays or an MRI to help diagnose your symptoms.

Here are some self-management methods that may help alleviate symptoms, but you should follow your doctor's advice:

Rest: Give the affected area sufficient time to rest and avoid any activities that cause pain.

Cold Compress: You can try applying a cold compress to the painful area for 15-20 minutes at a time, 3-4 times a day.

Appropriate Exercise and Stretching: Engage in low-impact exercises such as swimming or cycling to improve joint flexibility. Additionally, perform stretching exercises, especially for the calf and heel, which may be helpful.

Over-the-Counter Medications: Non-prescription non-steroidal anti-inflammatory drugs (NSAIDs) like ibuprofen or aspirin may help relieve pain and inflammation. Be sure to follow the medication instructions and be aware of potential side effects.

The information provided is for reference only, and a specific diagnosis and treatment should be carried out by a medical professional. If your symptoms persist or worsen, please seek medical help promptly.

31.

Your symptoms may be due to overuse of your joints from cycling or other activities. Cycling is an activity that places significant stress on the knee joints, and if you're not accustomed to cycling regularly, suddenly taking up cycling can lead to joint pain. This type of pain typically subsides gradually after discontinuing the overuse of the joints.

However, if the pain persists, worsens, or affects your daily life, you should seek a comprehensive evaluation at a hospital. While your symptoms may not be caused by a serious underlying condition, an examination can rule out any potential diseases such as inflammatory conditions (like arthritis) or injuries (such as tears or strains).

Here are some self-management methods that may help alleviate symptoms, but it's important to consult a doctor before proceeding:

Rest: Allow the injured area sufficient time to rest and avoid any activities that trigger pain.

Cold Compress: You can try applying a cold compress to the painful area for 15-20 minutes at a time, 3-4 times a day.

Appropriate Exercise and Stretching: Engage in low-impact exercises like swimming or cycling to improve joint flexibility. Additionally, perform stretching exercises, especially for the calf and heel, which may be helpful.

Over-the-Counter Medications: Non-prescription non-steroidal anti-inflammatory drugs (NSAIDs) like ibuprofen or aspirin may help relieve pain and inflammation. Be sure to follow the medication instructions and be aware of potential side effects.

The information provided is for reference only, and a specific diagnosis and treatment should be carried out by a medical professional. If your symptoms persist or worsen, please seek medical help promptly.

32.

Your symptoms may be due to a foot injury caused by physical activity, which can result in pain and swelling. If your symptoms persist or worsen, you should seek immediate medical attention.

You can try the following methods to alleviate your symptoms:

Rest and Elevate the Injured Area: Avoid standing and walking, and elevate the injured foot to reduce swelling.

Cold Compress: Apply a cold compress to the injured area multiple times a day, for about 15-20 minutes each time.

Over-the-Counter Medications: Non-prescription non-steroidal anti-inflammatory drugs (NSAIDs) such as ibuprofen may help relieve pain and swelling. Regarding the use of ibuprofen, I recommend contacting the healthcare professional who administered the vaccine first to ensure that using ibuprofen will not affect the vaccine's effectiveness. Additionally, this medication may have side effects, so it should be used as directed.

Physical Therapy: If swelling and pain persist, you may need to see a physical therapist who can provide treatment and rehabilitation exercises to help you regain normal mobility.

These are general recommendations, and for your specific situation, you should seek professional medical assistance at a hospital. If you experience severe pain, worsening swelling, or the emergence of other new symptoms such as fever, skin redness, or heat, you should go to the hospital immediately. These could be signs of a serious issue such as infection or vascular blockage.

33.

Your symptoms suggest the possibility of a hip joint condition, which may be related to osteoporosis. Osteoporosis can weaken the bone structure, making it more susceptible to fractures or dislocations. These conditions can lead to pain, limited mobility, and require a period of recovery.

First and foremost, I recommend seeking medical attention at a hospital as soon as possible and undergo a detailed assessment by an orthopedic or rheumatology specialist. Depending on your specific condition, imaging tests such as X-rays or MRI may be necessary to evaluate the condition of the hip joint.

Regarding the treatment of osteoporosis, calcium supplements and vitamin D are the foundation, but if osteoporosis is severe, more potent medications such as bisphosphonates or other drugs may be required.

In addition, weight management, moderate exercise, and proper nutrition are also crucial for preventing and treating osteoporosis. Consult with a doctor or nutritionist for specific recommendations.

It should be emphasized that if the hip joint dislocates frequently, surgical treatment may be necessary, such as hip replacement surgery. This should be evaluated and determined by an orthopedic specialist.

Furthermore, you mentioned that taking calcium supplements leads to leg cramps. This may be due to a deficiency in magnesium. Calcium and magnesium need to maintain a certain balance in the body, and a lack of magnesium can lead to muscle cramps. I recommend consulting a doctor to determine if magnesium supplementation is necessary.

34.

Hello, based on your description, your symptoms may be due to a recurrence of synovitis or overuse of the knee joint, leading to pain and discomfort. Synovitis is a type of joint inflammation that primarily affects the synovium within the joint, resulting in joint swelling and pain. The knee soreness you described may be a symptom of synovitis.

As for the cracking sound in your knee joint, if it's not accompanied by pain or swelling, it might be a normal phenomenon caused by the release of gas within the joint when the joint is stretched or twisted. This is medically referred to as "joint crepitus" and generally doesn't indicate a serious issue. However, if joint crepitus is associated with pain, swelling, or other discomfort, or if it occurs following a joint injury, it may suggest an underlying joint problem, such as arthritis or cartilage damage, and should be evaluated by a medical professional.

For your symptoms, I would recommend the following:

Reduce Joint Stress Appropriately: Try to avoid overusing the joint, especially in activities that may impact the joint, such as basketball. You can consider engaging in low-impact exercises like swimming or cycling.

Maintain a Healthy Weight: Excess weight can put extra pressure on the knee joint, exacerbating pain and inflammation.

Physical Therapy: Seek the assistance of a physical therapist who can provide guidance on how to protect your joints, alleviate pain, and improve joint function.

Medication: Over-the-counter medications like ibuprofen or other anti-inflammatory drugs may help reduce pain and inflammation.

Hot and Cold Compresses: Generally, cold compresses can help alleviate acute pain and swelling, while hot compresses can aid in muscle relaxation and increasing the joint's range of motion.

These are some temporary solutions, and you should discuss long-term management strategies with your doctor and undergo regular check-ups to monitor changes in your condition. If your symptoms persist or worsen, or if you experience any new symptoms, it's important to seek medical attention.

35.

Hello, your symptoms may not be typical of gout. Gout typically causes acute joint pain, especially at the base of the big toe, and it usually is not influenced by changes in weather or climate.

However, your symptoms may be more in line with rheumatoid arthritis, osteoarthritis, or a similar condition. Symptoms of these conditions can worsen with weather changes (such as humidity or changes in air pressure) or in cold weather. Additionally, nerve pain could also result in similar symptoms, including knee pain and nerve pain sensations in the buttocks.

Your symptoms should be evaluated by a doctor for a proper diagnosis. I recommend seeking medical attention as soon as possible, and you may need some tests, such as blood tests or imaging studies (such as X-rays or MRI), to determine your specific condition.

In the meantime, you have already taken some measures, such as using a heating pad, to alleviate your symptoms. Additionally, some over-the-counter medications, such as nonsteroidal anti-inflammatory drugs (e.g., ibuprofen), can help relieve pain and inflammation. However, the use of these medications should be done under the guidance of a doctor, as they may have some side effects.

36.

Hello, based on your description, it seems that your family member may be experiencing some age-related symptoms, including lower back pain and fatigue while walking. For such symptoms, especially in older individuals, it is advisable to seek medical advice first to determine the underlying cause and establish a treatment plan.

In some cases, musculoskeletal pain can be due to conditions like osteoarthritis or other joint-related disorders, or it may result from certain types of nerve compression. Further tests such as Magnetic Resonance Imaging (MRI) or electrophysiological examinations may be needed to determine the cause.

Regarding the medication "Shujin Jianyao Wan," this is a traditional Chinese medicine primarily used to treat lower back pain caused by liver and kidney deficiency. While in some cases, these medications may help alleviate symptoms, they do not treat the underlying cause. Without proper treatment of the cause, the symptoms may persist. Additionally, although Shujin Jianyao Wan has relatively few side effects, in some cases, it may lead to adverse reactions such as diarrhea, nausea, and stomach discomfort.

I recommend discussing this issue with their doctor to determine whether this medication is suitable for them based on their specific condition. Furthermore, for the issues of lower back pain and fatigue, physical therapy, especially a customized exercise program, can be very helpful. Physical therapists or rehabilitation doctors can provide personalized exercise plans tailored to their situation.

Regarding the issue of fatigue, if all possible medical causes have been ruled out (such as heart disease, respiratory disorders, etc.), it may be necessary to consider whether their lifestyle can be modified to improve their energy levels. This may include ensuring they get enough sleep, maintain a balanced diet, and engage in regular exercise.

37.

Hello, based on your description, this hard lump could have several possibilities:

Osteophyte: Osteophytes are a degenerative condition of the skeletal system, primarily caused by excessive bone growth due to wear and tear on the bones. This growth is typically the bone's attempt to repair itself through proliferation. Generally, this proliferation does not cause noticeable symptoms unless it compresses surrounding tissues and nerves, leading to pain.

Lipoma: Lipomas are benign soft tissue tumors mainly composed of fat cells. They typically form beneath the skin and feel soft and movable to the touch. Lipomas usually do not cause pain unless they grow and exert pressure on surrounding nerves.

Cyst: Cysts can also be responsible for the formation of a hard lump. They are typically composed of a cyst wall and inner filling material, such as fluid, semi-solid, or gas. In some cases, cysts may cause pain, especially if they become inflamed or infected.

To accurately determine the nature of this hard lump, I recommend consulting a doctor. The doctor may need to perform some tests, such as an ultrasound, X-ray, or CT scan. These tests can help the doctor understand the lump's nature, location, size, and whether it is affecting surrounding tissues. This information will assist the doctor in making an accurate diagnosis and developing a treatment plan.

38.

Hello, based on your description, the lump on the top of your foot could be a "tendon sheath cyst." Tendon sheath cysts, also known as "synovial cysts," typically occur within the sheath surrounding tendons. The cysts are filled with clear fluid and usually feel soft and movable to the touch, and they may not be painful or only mildly so. The size of the cysts can fluctuate, and sometimes they can self-resolve, but in some cases, they may grow larger.

However, besides tendon sheath cysts, there are many other conditions (such as lipomas, lymphomas, cysts, etc.) that could also lead to similar symptoms. Therefore, to accurately diagnose your condition, I recommend seeing a doctor. The doctor may conduct a physical examination and may require some imaging studies (such as ultrasound or MRI) to determine the nature and origin of the lump.

39.

Hello, congenital hip dislocation is a relatively common pediatric condition, and it predominantly affects females. Its etiology may involve various factors, including genetic factors, intrauterine positioning, and abnormal fetal hip joint development.

Regarding whether to undergo surgical treatment, this depends on multiple factors. These factors include your age, physical condition, the severity of symptoms, and your willingness to accept potential risks and complications. In your case, you already experience difficulty in walking and post-activity pain, which may impact your quality of life. Therefore, you may benefit from surgery.

The goal of surgery is typically to repair or reconstruct the hip joint to enable normal movement and minimize pain as much as possible. However, surgery may not always achieve a completely normal gait, and there may still be some mild gait abnormalities. Additionally, all surgeries carry certain risks, including infection, blood clots, nerve damage, and more.

Your situation may require evaluation by a multidisciplinary team, including orthopedic surgeons, physical therapists, rehabilitation doctors, and others. I recommend seeing a doctor and discussing your treatment options with them. They can explain potential treatment approaches, expected outcomes, and potential risks based on your specific circumstances.

40.

Hello, based on your description, these symptoms may be due to prolonged periods of maintaining the same posture, such as prolonged smartphone use, which can lead to tension in the neck muscles and result in numbness in the head and face, as well as a sensation of blood vessels pulsing. This may also be related to your diet, sleep, and lifestyle habits.

Additionally, the sensation of your fingers and toes feeling cool may be related to poor blood circulation, especially when you are not moving or when your hands and feet are held in a fixed position, which can slow down blood flow and lead to this sensation.

If you have recently been exposed to air conditioning or a cold environment, this cool sensation may also be a normal physiological response.

I suggest trying to change your daily habits, such as reducing the time spent on your smartphone, engaging in more physical activity to stretch your muscles and keep a good posture, and taking breaks to move around periodically, especially for your neck and hands and feet.

Furthermore, maintaining a healthy diet and including foods rich in vitamin B (such as lean meats, eggs, vegetables, and whole grains) can help support the normal functioning of your nervous system.

If your symptoms persist or worsen, I strongly recommend seeking medical attention promptly for a detailed examination and diagnosis. Additionally, considering your history of gynecological inflammation and the use of anti-inflammatory drugs, it's advisable to inform your doctor about these symptoms to see if any adjustments to your treatment plan are necessary.

41.

Your symptoms could be caused by conditions such as sciatica, avascular necrosis of the femoral head, hip arthritis, fasciitis, and more. These conditions often lead to pain in the hip, thigh, and even knee, especially when sitting or after physical activity.

Sciatica is caused by compression of the sciatic nerve and includes symptoms like pain, numbness, tingling, or weakness, typically affecting only one side of the body.

Avascular necrosis of the femoral head results from interrupted blood supply to the femoral head and commonly presents as hip pain, which can worsen with walking, climbing stairs, or twisting.

Hip arthritis is due to joint inflammation, and symptoms include pain and reduced mobility, especially after activity.

Fasciitis results from inflammation of the fascia (a thin membrane surrounding muscles) and includes symptoms of pain and stiffness.

I recommend seeing a doctor for a proper evaluation, which may include X-rays or MRI scans to pinpoint the specific cause of your symptoms. Additionally, trying gentle exercises such as yoga and stretching may help alleviate pain. Remember to start any new exercise program slowly and gradually increase intensity. Maintaining good posture, especially when sitting, can also aid in preventing and relieving pain.

42.

For the treatment of synovitis, common approaches typically include rest, cold compresses, physical therapy, anti-inflammatory medications, and sometimes even surgery. In your case, it seems you have already tried some of these methods without significant improvement, which may indicate the need for further evaluation and treatment.

Regarding the swelling on your knee, it could be due to inflammation or swelling caused by synovitis. Synovitis involves the excessive growth and inflammation of the synovium, a thin membrane within the joint, which can lead to joint swelling and pain.

The inability to fully straighten your leg may be due to joint swelling or the surrounding muscles and connective tissues becoming tight or shortened. This may require improvement through physical therapy and/or specific stretching and strengthening exercises.

At this point, I recommend revisiting your doctor for further examination and evaluation. The doctor may suggest imaging tests (such as MRI or ultrasound) to gain a clearer understanding of the internal condition of your knee. Based on this information, the doctor will be able to provide more specific treatment recommendations, which may include physical therapy, medication therapy, or potentially surgery.

43.

Your situation may have several possible reasons:

You may have re-injured your ankle. Although you don't recall a significant injury, certain activities, especially ones you may not be aware of after consuming alcohol, could lead to a minor sprain or injury.

An old injury may not have fully healed. If your ankle didn't receive proper treatment and adequate recovery time following the initial injury, an old injury may not have fully healed, leading to ongoing pain.

You may have inflammation. Structures around your ankle may have become inflamed after your ankle injury, especially if your ankle didn't receive proper rest and recovery time. Inflammation can lead to pain, warmth in the ankle, and even swelling.

In this case, I recommend seeking medical attention as soon as possible, especially if your symptoms persist or worsen. The doctor may suggest X-rays or other types of imaging tests to more accurately determine the nature and severity of the problem. They may also recommend specific treatment approaches, such as rest, physical therapy, or possible medication therapy.

44.

Hello, I can provide some possible explanations and suggestions, but I must emphasize that I am not a doctor; I am an artificial intelligence model and cannot replace a medical diagnosis. The symptoms you described could have various causes, including flat feet, plantar fasciitis, neuropathy, poor circulation, and more. I recommend seeking medical attention at a hospital to receive a proper diagnosis and treatment. This may involve undergoing tests such as blood tests, nerve conduction studies, ultrasound, or X-rays.

Before seeing a doctor, you can try the following:

Avoid prolonged standing or walking as much as possible. When resting, try to elevate your feet, which can help improve circulation.

Try wearing comfortable shoes with adequate support and cushioning. At home, consider wearing soft-soled slippers.

Perform foot and calf stretching exercises before bedtime and upon waking up. For example, sit down and roll a rolling object (such as a foam roller or a frozen water bottle) back and forth under your feet. Or stand on a step, let your heels sink down, and then raise them to stretch your heels and calf muscles.

Pay attention to staying warm, especially in cold weather. This can help maintain good blood circulation.

If your symptoms do not improve or if there is any worsening of pain or conditions, you should go to the hospital's emergency department immediately. If prescribed medication by a doctor, make sure to use it correctly following the doctor's instructions. Remember, whether online or elsewhere, you should not seek or accept medication recommendations without a doctor's permission.

45.

Hello, the symptoms you described may be related to several conditions, but please note that I am not a doctor; I am an artificial intelligence model and cannot replace a medical diagnosis.

Impact of Cold Weather on Blood Flow: Cold weather can cause blood vessels to constrict, which may lead to pain in your feet in cold conditions. Try to keep warm, wear thick socks and warm shoes to see if it helps alleviate the pain.

Footwear: Your shoes may have an impact on your foot discomfort. If your shoes lack adequate support or are too narrow, they could lead to foot pain. Try wearing a better pair of shoes to see if it alleviates the pain.

Plantar Fasciitis: This is a common foot condition that can cause pain in the sole of the foot, particularly the heel. The plantar fascia is a thick tissue connecting the toes and the heel, and if inflamed, it can cause pain.

Flat Feet or Fallen Arches: If the arch structure of your feet is abnormal, it may lead to foot pain. Particularly, flat feet can unevenly distribute pressure on the feet, causing pain.

Pain may also be related to your walking posture. If you have an improper gait or walking style, it could lead to foot or other body part discomfort.

Again, you should consult a doctor to obtain the correct diagnosis and treatment. They may conduct tests such as X-rays, ultrasound, or MRI to determine the cause of your pain. If it is plantar fasciitis or another foot issue, physical therapy, special insoles or shoes, medication, or, in some cases, surgery may help.

Before seeing a doctor, taking these measures may help alleviate pain:

Perform foot and calf stretching exercises.

Use hot and cold therapy: Cold packs can help reduce inflammation, and warm packs can help relax muscles and increase blood flow.

Consider using over-the-counter pain relievers like ibuprofen, but follow the instructions and avoid prolonged use.

Rest as much as possible and avoid prolonged standing or walking.

However, if the symptoms persist or worsen, or if you have any other health concerns, you should contact a doctor as soon as possible.

46.

Hello, your mother's symptoms could be related to many different conditions. Knee pain is a very common symptom and can be caused by wear and tear, injury, or diseases such as arthritis. It's important to note that I am not a doctor, and I cannot replace a doctor's diagnosis. Here are some possible causes and recommendations:

Knee Arthritis: This includes osteoarthritis and rheumatoid arthritis, among others. Pain is typically worse after activity, especially climbing stairs, squatting, or standing for extended periods. The joint may also swell and sometimes feel warm.

Patellofemoral Syndrome: This is a common condition that causes pain at the front of the knee, especially when going downstairs or on slopes. It's due to improper movement of the patella (kneecap) in the groove of the femur (thigh bone).

Meniscus Injury: The meniscus in the knee is two C-shaped pieces of cartilage that act as cushions in the knee joint. Twisting or overuse can lead to meniscus injuries.

Your mother should visit a larger hospital for a thorough examination, including X-rays, MRI scans, and other tests to determine the specific cause of the pain. She may need to take medications such as non-steroidal anti-inflammatory drugs (NSAIDs) to relieve pain and swelling. Physical therapy may also be beneficial, including heat and cold therapy, electrotherapy, and knee-strengthening exercises. In some cases, surgical treatment may be necessary.

Before seeing a doctor and getting a definitive diagnosis, your mother can try the following methods to alleviate pain:

Rest: Avoid overusing the knee joint, especially activities that require exertion.

Ice Pack: Applying an ice pack to the knee can help reduce pain and swelling.

Elevation: Elevating the foot while resting can help reduce swelling.

Gentle Exercise and Stretching: Some gentle exercises and stretching can help maintain knee joint mobility, but they should be done without causing pain.

47.

First and foremost, I must emphasize that I am just an artificial intelligence and cannot replace the advice or diagnosis of a doctor. Before attempting any new exercises or rehabilitation activities, you should consult with a medical professional or physical therapist. However, I can provide some general suggestions that you may try at home to help alleviate some hip pain:

Muscle Stretching: Stretching the muscles around the front and back of your thighs can relieve hip joint pressure. You can try sitting on the floor with your legs extended and attempt to lean forward, reaching for your toes as much as possible. Another stretching technique is to create a "4" shape with one leg crossed over the other and then try to lean forward.

Bridge Exercise: Lie on the floor with your feet flat on the ground and knees bent. Then raise your hips, keeping your back and thighs in a straight line. This movement can exercise your glutes and the muscles on the back of your thighs, thus alleviating pressure on the hip joint.

Wall Squats: Stand against a wall and slowly squat down until your knees and hips are at a 90-degree angle. Hold this position, starting with 15 seconds and gradually increasing over time.

You mentioned having resistance bands at home, and these rehabilitation exercises can also be performed using resistance bands:

Band Walks: Secure the resistance band around your legs and then try to walk as you normally would. This exercise can help work your glutes and the muscles on the outer side of your hips.

Band Hip Abduction: Attach the resistance band around your legs, lie on one side, and then attempt to raise the upper leg outward. This exercise can also help work your glutes and the muscles on the outer side of your hips.

Consistency is key. While doing these exercises, you may experience muscle soreness, which is normal. However, if you experience severe pain or an exacerbation of hip pain, you should stop these exercises and consult a doctor as soon as possible.

Lastly, while these exercises may help relieve pain, it doesn't mean you shouldn't seek the underlying cause. Please get an MRI scan at the hospital to identify the cause of your hip pain once the pandemic subsides.

48.

First and foremost, I want to emphasize that I am an artificial intelligence, not a doctor, and I cannot make a diagnosis. However, I can provide some advice based on the information you've provided.

Your father's symptoms appear to be consistent with osteoarthritis, which is a common condition characterized by the wear and tear of joint cartilage, especially in middle-aged and older individuals. Typical symptoms include joint pain, swelling, and difficulty with movement. Osteoarthritis is a chronic condition that cannot be fully cured, but its symptoms can be managed through various means, including medications, physical therapy, and exercise.

In terms of medications, over-the-counter drugs like ibuprofen or naproxen may help alleviate pain and swelling. Prescription medications such as COX-2 inhibitors (e.g., celecoxib) may also be effective in relieving symptoms. If the pain is severe, your doctor may recommend corticosteroid injections.

On the non-pharmacological treatment side, physical therapy can help improve joint flexibility and strength. Customized exercise plans can help strengthen the muscles around the knee joint and reduce joint stress. Additionally, if overweight, weight loss may also help reduce the strain on the knee joint, thus relieving pain.

If the above management strategies are not effective in alleviating symptoms, your doctor may recommend surgical options such as joint replacement.

Remember that while I've provided some potential treatment suggestions, these should all be carried out under the guidance of a medical professional. Therefore, it's advisable to take your father to see a doctor to discuss a detailed treatment plan.

49.

I want to emphasize that I am an artificial intelligence model, and I cannot diagnose diseases. When it comes to health issues, it's essential to see a doctor as soon as possible, especially when your symptoms are unexplained and severe. Your symptoms may include the following possibilities:

Sprain or Strain: If you've recently engaged in strenuous physical activity or have had a recent injury to your foot, it could result in muscle, ligament, or tendon sprains or strains.

Gout: Gout is a form of arthritis caused by the formation of uric acid crystals in the joints. The most common site for gout is the big toe, but it can also affect other parts of the foot and ankle. Symptoms typically include severe pain, redness, and warmth.

Infection: If you've had a recent cut or break in your foot, it's possible that an infection has developed, leading to redness and pain.

Blood Clot: In very rare cases, redness and pain in the foot could be due to the formation of a blood clot, blocking blood flow. This is a medical emergency and requires immediate attention.

These are just some of the possible causes, and if your symptoms persist, you should seek medical help promptly to obtain the correct diagnosis and treatment.

50.

There could be several possible reasons for your symptoms:

Metatarsalgia: This is a common condition in the foot characterized by pain in the ball of the foot, especially during walking and physical activity. It can be caused by factors such as prolonged standing, overuse, ill-fitting shoes, or being overweight.

Nerve pain: Compression or diseases affecting the nerves in the foot can lead to pain. For example, Morton's neuroma is a common foot nerve condition that typically occurs between the third and fourth toes but can also affect the big toe.

Hallux Pressure Point Inflammation: This is inflammation caused by prolonged pressure or irritation. It may be due to walking style, ill-fitting shoes, or structural foot problems.

Gout: This is a form of arthritis that often affects the big toe but is typically accompanied by other symptoms such as redness and warmth.

If your symptoms persist or worsen, it is advisable to consult a doctor for a proper diagnosis and treatment. Additionally, if possible, try to avoid prolonged standing and walking and wear appropriate and comfortable footwear to help alleviate the pain.

51.

Hello, it is indeed advisable for gout patients to limit the intake of high-purine foods. Purines in the body can be converted into uric acid, and excessive purine consumption can lead to elevated uric acid levels, triggering gout. Here are some general recommendations regarding the purine content of foods:

Low-purine foods (can be consumed in moderation):

Most vegetables and fruits: Examples include carrots, lettuce, cucumbers, tomatoes, apples, bananas, etc.

Whole grains and legumes: Such as oats, brown rice, and legumes. However, some legumes like lentils, peas, and chickpeas contain higher levels of purines and should be consumed in moderation.

Low-fat dairy products: Such as skim milk, low-fat yogurt, etc.

Eggs

Moderate-purine foods (should be consumed in moderation):

Poultry such as chicken, turkey, and rabbit: These meats have relatively lower purine content but should still be consumed in moderation.

Fish and seafood: Such as eel, cod, lobster, etc. While some fish and seafood have relatively lower purine content, gout patients should still consume them in moderation due to potential health risks.

High-purine foods (should be avoided whenever possible):

Red meat: Such as beef, lamb, pork, etc.

Seafood: Such as shellfish (e.g., scallops, clams, crab), and fish roe.

Organ meats: Such as liver, kidneys, hearts, etc.

Some legumes: Such as soybeans, lentils, peas, and chickpeas.

Brewer's yeast, such as beer yeast.

These recommendations are general in nature, and their applicability may vary from person to person. You should adjust your diet based on your body's response and guidance from your doctor. Managing gout is not only about diet but also includes maintaining a healthy weight, engaging in appropriate exercise, and avoiding excessive alcohol consumption. Additionally, for individuals with elevated uric acid levels, drinking plenty of water is crucial as it helps the body eliminate excess uric acid.

52.

The symptoms you described may be related to Achilles tendonitis or plantar fasciitis. Both conditions can cause heel pain, especially when walking or standing.

Achilles tendonitis is caused by overuse or injury to the Achilles tendon, which is located at the back of the ankle. It can lead to pain in the heel or the back of the calf.

Plantar fasciitis is a common foot problem that occurs under the heel bone on the sole of the foot. It results from overstretching or tearing of the plantar fascia, a thick band of tissue that connects the heel bone to the toes, leading to inflammation and pain. Its symptoms are typically most severe in the morning when getting out of bed and gradually improve with walking.

Both of these conditions can be managed through physical therapy, including stretching and strengthening exercises, cold and hot compresses, and/or over-the-counter medications such as pain relievers. Sometimes, further treatment may be needed, such as orthopedic shoe inserts or, in severe cases, surgery.

I recommend that you see a doctor as soon as possible to obtain a proper diagnosis and treatment. Before that, you can try some self-management techniques, such as reducing the time spent standing and walking, applying cold packs to the painful area, and doing gentle foot and calf stretches. However, you should avoid any activities that worsen the pain.

Please note that this is just one possible explanation based on the symptoms you described and cannot replace a doctor's diagnosis.

53.

The symptoms you described may be related to a metatarsal stress fracture, especially if you are a runner. Metatarsal stress fractures are common sports injuries, particularly among individuals who engage in regular running or walking. They typically occur in the front or top part of the foot.

Symptoms may include:

Pain during or after activity, especially when walking or running.

Pain in a specific area on the top of the foot.

Pain that is more severe when starting to walk or run and may lessen as the activity continues.

If your pain does not subside after continuing to run or walk, or if it persists even after resting, you should seek medical attention promptly. An X-ray or MRI may be necessary to confirm the diagnosis.

Before that, you may need to stop running and any activities that may exacerbate the pain, apply cold packs, and possibly take over-the-counter pain relievers like ibuprofen to help alleviate the pain. However, you should avoid any activities that worsen the pain.

Please note that this is just one possible explanation based on the symptoms you described and cannot replace a doctor's diagnosis.

54.

Hello, from your description, it appears that the raised small bump may be a "ganglion cyst." Ganglion cysts typically occur near joints in the wrist, hand, or foot. These cysts are fluid-filled sac-like structures that usually do not cause serious issues but may cause some discomfort. They often move with the movement of the hand or when making a fist and may be tender to the touch.

However, if your cyst becomes painful after squeezing or increases in size, it may indicate irritation or injury. In such a case, you should see a doctor. The doctor may recommend various treatment options, including aspiration (removing the fluid from the cyst with a needle), and if the cyst recurs or causes pain, surgery may be necessary.

Remember that the information I provide is based solely on your description. An accurate diagnosis should be made by a doctor after a thorough examination. So, please seek medical attention promptly.

55.

Hello, based on your description, this red lump could be caused by various reasons. It may be due to a condition called osteoarthritis, which is a form of arthritis that causes joints to become stiff, painful, and inflamed. Osteoarthritis typically affects middle-aged and older individuals but can also affect younger people, especially if they have a history of joint injuries or overuse.

Another possibility is rheumatoid arthritis, which is an autoimmune disease that causes inflammation and pain in the joints. However, this condition typically affects multiple joints, not just one.

If this red lump continues to enlarge and is painful, it's important to see a doctor as soon as possible. They may recommend various tests, such as X-rays or blood tests, to help determine the cause of this lump. Over-the-counter pain relievers may help alleviate the pain until a diagnosis is confirmed.

56.

Hello, your symptoms could have several possible causes:

Soft Tissue Lump: This could be due to soft tissue tumors or cysts, such as lipomas, fibromas, neurofibromas, etc. These are usually benign and may change in size and color over time.

Folliculitis or Sebaceous Cyst: If the red and swollen area is on the skin, it may be due to folliculitis (inflammation of hair follicles) or a sebaceous cyst caused by blocked sebaceous glands.

Arthritis: If the lump is near a joint, it could be a sign of gout or another form of arthritis. This is typically accompanied by pain and swelling.

If you don't have any other symptoms such as pain, fever, signs of infection (such as pus), restricted movement, etc., the lump may not be a serious issue. However, if it persists, enlarges, or if you develop other symptoms, you should see a doctor. The doctor may need to perform further examinations, including imaging tests (such as ultrasound or MRI) or a biopsy, to determine the nature of the lump.

Please note that these are possible explanations, and it's important to seek medical attention for a proper evaluation.

57.

Hello, your symptoms could be related to several conditions:

Ganglion Cyst: This is a fluid-filled cyst that typically occurs on the wrist or fingers. The size of the cyst may change with hand movement. They are usually painless but can cause discomfort or numbness if they press on nearby nerves.

Lymphangitis: This is inflammation of the lymphatic vessels (tubes that carry lymph fluid throughout the body). It typically presents as red or pink streaks on the hand or foot and may be accompanied by fever or pain. However, this condition often comes with other symptoms like redness and fever.

Tenosynovitis: Prolonged use of the hand, such as typing or lifting heavy objects, can lead to tenosynovitis, which is inflammation of the sheath surrounding a tendon (fibrous tissue that connects muscles to bones). It often results in hand pain and swelling, especially with hand use.

These are just possibilities and may not necessarily be accurate. The best advice is to see a doctor. The doctor may need to perform further examinations to determine the cause of this small bump and recommend appropriate treatment methods.

58.

For a tendon sheath cyst during pregnancy, it's important to emphasize that any medical decisions should be made under the guidance of a healthcare professional. While some treatment options for a tendon sheath cyst are theoretically safe for pregnant women and their fetuses, these choices should be made after assessment and guidance from your medical team.

Conservative Treatment: Typically, the preferred approach is conservative non-surgical treatment, including using a wrist splint to reduce wrist movement, alleviate pressure, and manage pain. Physical therapy may also be helpful.

Needle Aspiration: In some cases, a doctor may choose to aspirate the cyst using a fine needle to extract the fluid inside. This procedure is usually done under local anesthesia. However, a drawback of this method is that the cyst may refill and recur.

Surgery: Surgery may be necessary if the cyst becomes large enough to interfere with daily life or if non-surgical methods prove ineffective. However, in general, it is advisable to avoid unnecessary surgery during pregnancy.

"Rupturing" the cyst is not a recommended practice. While some individuals may find that cysts rupture naturally under certain pressure, attempting to rupture a cyst artificially can lead to complications such as infection and should be avoided.

Please contact your doctor or medical team to discuss your specific situation and potential treatment options. They can take into consideration your pregnancy and provide you with the most suitable treatment recommendations.

59.

Hello, a tendon sheath cyst is a common soft tissue cyst that typically occurs on the wrist, hand, or foot. They are usually painless unless the cyst becomes large enough to compress nearby nerves. These cysts result from the leakage of fluid from a joint capsule or tendon sheath, collecting within a cystic structure.

Regarding the management of a tendon sheath cyst, here are the typical recommendations:

Observation and Wait: If the cyst is not causing pain and does not affect your hand's function, a doctor may recommend observation and waiting for some time. Some tendon sheath cysts may resolve on their own.

Non-Surgical Treatment: This may include using a wrist splint or bandage to restrict wrist movement, reducing pain and the size of the cyst. Sometimes, a doctor may choose to aspirate the cyst using a fine needle to remove the fluid inside. This method can be effective at times, but cysts may refill and recur.

Surgery: If the cyst causes pain, impairs hand function, or doesn't improve after a period of observation, surgery may be recommended. Surgery is typically performed under local anesthesia, and the goal is to completely excise the cyst to reduce the likelihood of recurrence.

Each person's situation is unique, and your doctor will recommend the most suitable treatment plan based on your specific circumstances. If the cyst is not causing pain and doesn't significantly impact your daily life, observation and wait may be a viable option. If the cyst is painful or affects hand function, non-surgical treatment or surgery may be considered. It's important to discuss the various options and potential risks and benefits with your doctor to make the best decision.

60.

Hello, based on your symptoms, you may be experiencing a herniated lumbar disc, which can cause back pain while compressing nerves, leading to pain, numbness, or tingling radiating into the buttocks and legs.

For your first question regarding whether surgery is needed, it depends on the severity of your symptoms and the effectiveness of non-surgical treatments you've tried. Typically, the preferred treatment approach is non-surgical methods such as physical therapy, pain management (including over-the-counter or prescription medications), and lifestyle changes like weight management and core muscle strengthening exercises. Surgery is usually considered when non-surgical treatments are ineffective or when symptoms are severe enough to impact your quality of life, such as severe pain, inability to walk, or significant neurological symptoms (such as leg weakness or loss of bladder and bowel control). Specific medications and rehabilitation exercises would require detailed guidance from your doctor or physical therapist.

Regarding your second question about the burning sensation on the soles of your feet, this symptom may be related to the herniated lumbar disc, especially if a nerve is being compressed. A herniated lumbar disc can lead to sciatica, which is a condition where pain, numbness, or tingling radiates from the lower back down to the buttocks and legs, sometimes even to the feet. This may also explain the burning sensation you're feeling on the soles of your feet.

It's important to note that while this information may be helpful to you, you should reach out to your doctor or other healthcare professionals who can provide personalized advice based on your individual health condition. I hope this information is of assistance to you.

61.

Hello, your symptoms could be due to long periods of sitting and poor posture, leading to tension or strain in the lower back muscles. Prolonged sitting, especially with incorrect posture, can put pressure on the lower back and spine, resulting in muscle tension and pain.

The pain in the tailbone and lower back may also be related to issues with the vertebral discs, such as disc herniation or degenerative disc disease, which can cause pain in the lower back, tailbone area, and on both sides of the lower back.

Further examination is needed to determine the exact cause. You may require lumbar X-rays or an MRI to assess the condition of your spine and intervertebral discs. These tests can help identify if there are fractures, osteoporosis, disc problems, or other issues that may be causing your lower back pain.

As for pain relief, pain management may include over-the-counter medications like ibuprofen or acetaminophen, physical therapy, and correcting poor sitting posture. Changing your workstation setup, such as using a standing desk or an ergonomic office chair, and taking regular breaks to stand or walk can also help relieve lower back pressure.

However, it's important to consult a doctor to receive the best advice and treatment plan tailored to your individual situation. I hope this information is helpful to you.

62.

Hello, based on your description, your symptoms may be related to poor posture over an extended period or excessive use of computers and mobile phones. It could potentially be a manifestation of cervical strain or cervicalgia, which can lead to muscle tension in the neck and shoulders, resulting in pain and discomfort. However, a definitive diagnosis would require a clinical examination by a doctor.

For such symptoms, you may benefit from an initial assessment. First, seek the assistance of a healthcare professional who can conduct a physical examination, inquire about your work environment and daily habits, and understand the specific location, nature, and impact of your pain. This can help the doctor assess which potential underlying causes might be related to your symptoms.

If deemed necessary by the doctor, they may recommend further tests such as X-rays, CT scans, or an MRI of the cervical spine to gain insights into the structure of your neck and potential issues.

Once a precise diagnosis is obtained, treatment options may include physical therapy, pain medications, muscle relaxants, and modifying habits in daily activities or work environments. This could involve taking regular breaks, performing neck and shoulder stretching exercises, and adjusting the posture when using computers and mobile phones.

63.

For pain relief, in addition to over-the-counter medications like ibuprofen, resting and applying heat may help alleviate symptoms. Prolonged computer use can lead to tension in the neck and shoulder muscles, so appropriate neck and shoulder exercises and applying heat might be beneficial. If you need to purchase medication, you can consider buying some over-the-counter pain relievers like ibuprofen, paracetamol, or aspirin, which are common pain relievers. However, please follow the instructions on the label and be aware of potential side effects.

After your symptoms improve, if your physical condition allows, I still recommend that you seek medical attention promptly. The symptoms you described could be caused by various factors, such as muscle tension, nerve compression, heart issues, or other reasons. High uric acid levels are typically associated with gout, which primarily affects joints, particularly the big toe joint. While less common, it can also affect other areas. However, your symptoms seem more likely related to musculoskeletal or nerve issues.

In daily life, maintaining appropriate physical activity, avoiding prolonged periods in the same position, and regularly performing neck and shoulder stretching exercises can help prevent such issues. Additionally, maintaining a healthy diet, avoiding high-purine foods like red meat and organ meats, can help control uric acid levels. If you have any dietary concerns, you may want to consult with a nutritionist.

Please remember that these are general recommendations, and your specific symptoms need to be assessed by a medical professional. If your symptoms persist, worsen, or new symptoms arise, such as difficulty breathing, chest pain, dizziness, etc., seek medical help immediately, even in the midst of a severe pandemic situation.

64.

Hello, based on the symptoms you described, it's possible that you are experiencing neck muscle tension or cervical spine issues. Prolonged use of smartphones and computers, improper sleep positions, and inadequate physical activity can all contribute to this condition.

Here are some suggestions that may help alleviate your symptoms:

Change your sitting posture and electronic device usage habits: Ensure that screens are at eye level, maintain proper sitting posture, and take regular breaks to stretch and move around.

Sleep position: The sleep position you described might put pressure on your cervical spine. Try adjusting your sleeping position, and using a supportive pillow for your neck may be helpful.

Perform gentle neck and shoulder stretching exercises, such as slight left-right rotation and forward-backward tilting.

Applying heat may also help relieve muscle tension. You can use a hot towel or a heating pad on the painful area.

Whenever possible, consider engaging in some exercise to improve muscle strength, such as yoga or aquatic exercise.

If the pain persists, over-the-counter medications like ibuprofen or aspirin may provide some relief. However, remember to use them according to the package instructions and be aware of any potential side effects.

These suggestions are for reference only and cannot replace professional medical advice. Since it's currently not possible to visit a hospital for a detailed medical examination, these are preliminary assessments. I recommend that you seek medical attention as soon as conditions allow.

If your symptoms worsen or if new symptoms such as severe headaches, dizziness, nausea, vomiting, visual problems, or limb weakness occur, seek medical help immediately.

65.

Firstly, your symptoms may be related to cervical spine issues or other neurological conditions. These symptoms could include neck pain, dizziness, nausea, shoulder and back pain, and more. Based on your description, your symptoms worsen when looking down to read a book or use your phone, which could be due to compression of the cervical spine on nerves. Additionally, you mentioned that lifting your arms upwards provides some relief in your neck and upper shoulder blade area, which may alleviate pressure on the cervical spine.

It's advisable to seek the assistance of a medical professional at a hospital. They may recommend undergoing tests such as magnetic resonance imaging (MRI) or X-rays to determine the specific issue.

Furthermore, maintaining proper posture, avoiding prolonged periods of looking down at electronic devices, and regularly performing neck exercises can help alleviate your symptoms. When using a computer or phone, consider elevating the screen to eye level to reduce pressure on the cervical spine.

However, please bear in mind that these are suggestions and not medical advice. You should contact a doctor as soon as possible and describe your symptoms to receive the most accurate diagnosis and treatment recommendations.

66.

Hello, your wife's symptoms could be caused by various factors, including neurological issues, skeletal problems, or circulatory issues.

Neurological issues: Problems in the spine or neck could lead to numbness or pain in the hands and feet, which might be the result of nerve compression or irritation. For example, sciatica can cause numbness and pain from the lower back down to the feet.

Skeletal problems: Issues related to the skeletal structure, such as osteoporosis, arthritis, or hip joint problems, can also result in pain and discomfort.

Circulatory issues: If there are circulatory problems, it could also lead to numbness and pain in the hands and feet. This could be due to issues with the circulatory system, such as arteriosclerosis, deep vein thrombosis, and more.

The relief of symptoms when lying flat might be because this position reduces pressure on the nerves or blood vessels. However, a detailed medical evaluation is needed to determine the exact cause.

I strongly recommend taking her to the hospital for further examination as soon as possible. It may require more comprehensive tests such as nerve conduction velocity testing, magnetic resonance imaging (MRI), or blood tests to pinpoint the source of the problem.

67.

Hello, based on your description, your symptoms may be related to compression of the cervical nerves, possibly due to a straightening of the normal cervical lordosis curve. Numbness in the fingers, especially the ring finger and pinky finger, may be due to compression or irritation of the ulnar nerve (located on the inner side of the arm and responsible for the sensation in the ring finger, pinky finger, and part of the palm).

The choice of treatment will depend on your specific situation and the severity of your symptoms. In many cases, non-surgical approaches can effectively relieve symptoms, such as physical therapy, performing neck and back stretching and strengthening exercises, and making lifestyle modifications that can exacerbate symptoms.

Additionally, try to avoid maintaining the same position for extended periods, especially a forward-leaning neck posture. Consider taking breaks to stand up and stretch every so often, or using a computer stand that allows for height and angle adjustments. Maintaining good posture and minimizing excessive pressure on the neck and shoulders is crucial.

Regarding sleep, you may need to adjust your sleeping position and choose an appropriate pillow. Some people find that using specially designed cervical pillows can provide better support and reduce symptoms.

However, if the symptoms are severe, non-surgical treatments are ineffective, or if there is a worsening of nerve function, surgical intervention may need to be considered. Nevertheless, you must consult with a doctor or a qualified healthcare provider who will tailor the most suitable treatment plan based on your specific circumstances and needs. The symptoms you've described may require more detailed examinations and assessments by a physician to ensure the most appropriate course of action.

68.

Based on the symptoms you described, you may have cervicogenic dizziness, which is a symptom caused by neck issues, especially cervical spine disorders, that can lead to headaches, dizziness, and even nausea and vomiting. Here are some possible treatment approaches:

Physical Therapy: Physical therapists can provide specific exercises and stretches for your neck and shoulders to help manage symptoms. These exercises may help reduce muscle tension, improve blood flow, and potentially address cervical disc protrusion.

Medication: Over-the-counter medications like non-steroidal anti-inflammatory drugs (NSAIDs) may help alleviate pain and inflammation. In some cases, your doctor might recommend muscle relaxants or prescription pain medications. If dizziness is severe, certain medications like meclizine may help manage these symptoms.

Neck Traction: In some situations, neck traction may help relieve pressure from cervical disc protrusion, which could aid in alleviating your symptoms.

Surgery: If the above treatments are ineffective, and your symptoms significantly impact your daily life, your doctor may suggest surgical treatment. The specific type of surgery will depend on your individual circumstances.

In addition to these options, making lifestyle changes may also help improve your symptoms. For example, taking regular breaks and changing positions, avoiding prolonged static postures, and using an appropriate pillow for neck support during sleep can be beneficial.

Lastly, I want to reiterate that you should have a detailed discussion with your healthcare team about these treatment options and choose the most suitable approach based on your specific situation.

69.

Hello, your symptoms could be caused by various factors, including neurological, muscular, joint, or circulatory system issues, or they may be related to your hyperthyroidism (Graves' disease) or medication side effects. While I cannot provide a specific diagnosis, I can suggest some possible scenarios:

Nerve Compression or Fatigue: Prolonged, repetitive movements or maintaining the same posture, such as extended computer use, can lead to nerve or muscle fatigue or nerve compression, resulting in aches and pains.

Medication Side Effects: Medication side effects could also cause similar symptoms. Both Methimazole (used to treat hyperthyroidism) and Propranolol (an anti-anxiety medication) may have side effects like muscle soreness or fatigue. You should discuss this issue with your doctor to see if medication dosage adjustments or alternatives are possible.

Effects of Hyperthyroidism: Hyperactive thyroid (hyperthyroidism) can cause various symptoms, including muscle aches, fatigue, and anxiety. While your hyperthyroidism may be controlled, it might still affect your body.

Psychological Stress or Anxiety: Psychological stress or anxiety can lead to various physical symptoms, including muscle tension and pain.

Arthritis or Myositis: Although your pain is not joint-specific, certain conditions like arthritis or myositis can also cause muscle pain.

I recommend seeing a doctor for further examination and diagnosis. Your doctor may perform tests such as blood tests, nerve conduction velocity tests, or magnetic resonance imaging (MRI) based on your symptoms and medical history to determine the cause. Your doctor can also provide advice on symptom relief, such as lifestyle changes, physical therapy, or medication adjustments.

70.

Hello, your mother's symptoms indeed could be related to cervical spine issues, especially if her work or daily activities involve prolonged periods of maintaining the same posture, such as sitting for extended periods or prolonged use of smartphones or computers. These behaviors can lead to increased pressure on the cervical spine, which can further result in nerve compression and symptoms like numbness in the hands, neck and shoulder pain, etc. Cold air conditions may exacerbate muscle tension and worsen these symptoms.

However, I must emphasize that I can only provide possibilities, not a definitive diagnosis, as there are multiple potential factors based on your description. These could include conditions like peripheral nerve disorders (e.g., thoracic outlet syndrome, which can compress arm nerves leading to symptoms), arthritis, neuropathy, and more.

There are no specific medications I can recommend at this stage because a definitive cause needs to be identified before determining a treatment plan. Therefore, I strongly advise your mother to seek a diagnosis from a healthcare professional as soon as possible. If visiting a hospital is not possible due to the pandemic, consider telephonic or online consultations with a doctor.

However, here are some lifestyle habits and exercise recommendations that may help:

Improve Posture and Workspace: Maintain proper posture, ensure that the computer screen is at or slightly above eye level, and avoid prolonged periods of looking down. Take regular breaks to stretch and move the neck and shoulders.

Physical Therapy for Neck and Shoulders: Trying some neck and shoulder stretches and strengthening exercises can sometimes help alleviate symptoms. However, it's best to consult a doctor or physical therapist before starting a new exercise regimen.

Avoid Repetitive Strain and Overexertion: Avoid repetitive wrist, hand, and shoulder movements, especially gripping or twisting motions. Avoid lifting heavy objects, especially with one hand.

Maintain a Healthy Diet and Sleep Routine: A varied and balanced diet, avoiding greasy and high-salt foods, and ensuring adequate sleep can aid in the body's recovery.

You mentioned "Zhuifeng Tougu Wan capsules," which are primarily used to relieve musculoskeletal pain. If your mother finds it helpful, she can continue taking them under the guidance of a doctor. However, if the symptoms persist, she should seek professional medical help.

71.

Hello, the symptoms you've described could have several possible causes, such as poor posture, prolonged periods of improper posture (e.g., extended use of computers or smartphones), lack of exercise, muscle tension, or strain, all of which can lead to your symptoms. Additionally, nerve compression, such as cervical spondylosis or lumbar spondylosis, could also result in similar symptoms.

Based on your description, another possibility is a peripheral nervous system issue. The peripheral nervous system consists of nerves that extend from the brain and spinal cord, controlling sensations and movements in the body. When these nerves are damaged or compressed, symptoms like pain, numbness, or muscle weakness can occur.

In this case, first, I would recommend trying to change your lifestyle habits, including:

Improve Your Work or Study Environment: Maintain proper sitting and standing postures as much as possible and avoid prolonged periods of the same posture, especially when using a computer or smartphone.

Increase Physical Activity: Regularly engage in stretching and relaxation exercises, such as yoga or Pilates, to help alleviate muscle tension and pain.

Ensure Adequate Sleep: Sleep is crucial for the body's recovery and repair, so ensuring sufficient sleep can aid in relieving pain.

If, after changing these habits, the symptoms persist or worsen, I suggest seeking medical help as soon as possible for further assessment and treatment. During this process, some tests, such as nerve conduction velocity testing, may be necessary to determine if there is nerve damage. This will help identify the correct treatment plan.

72.

Hello, based on your description, headaches and nausea can have various potential causes.

Firstly, you mentioned prolonged computer use, which could lead to cervical strain and discomfort in the neck, a common phenomenon known as "office syndrome" or "cervical spondylosis." This may cause neck and shoulder pain and could even lead to headaches. Taking regular breaks, improving your working environment and posture, and performing periodic neck stretching exercises can help prevent these symptoms.

Secondly, headaches and nausea might also be related to your diet. Certain components in food (such as monosodium glutamate or chocolate), excessive alcohol consumption, or eating spoiled food can trigger headaches and nausea. If your symptoms occur after consuming a specific type of food, that might be the cause.

Furthermore, the symptoms of headaches and nausea you described could also be attributed to cold weather. Cold temperatures can cause blood vessels to constrict, leading to headaches. Prolonged immobility in a cold environment may result in muscle stiffness, which could also trigger headaches.

Lastly, migraines could be another potential cause of headaches and nausea. Migraines typically come with some warning signs, such as visual disturbances, nausea, sensitivity to light or sound, etc. Migraines may require specific treatments, so if your symptoms align with migraine characteristics, it's advisable to seek medical help.

If your symptoms of headaches and nausea persist or worsen, I recommend consulting a doctor as soon as possible. These symptoms could be early signs of more serious conditions, such as brain tumors or cerebrovascular diseases, and prompt medical attention may be necessary.

73.

Hello, based on your description, your symptoms may indeed be related to maintaining a single position for an extended period, especially prolonged periods of looking down at a computer or reading. This can lead to tension in the neck muscles and compression of the cervical spine, potentially causing headaches. This condition is known as "cervicogenic headache." Additionally, the dizziness and nausea you mentioned could also be related to cervical spine issues, as compression of the neck may affect the blood vessels in the back of the neck, potentially leading to these symptoms.

However, I must emphasize that I cannot provide a definitive diagnosis as I am an artificial intelligence and have not conducted a face-to-face assessment with you.

Regarding scoliosis, scoliosis typically does not directly cause headaches, but it may alter your overall body posture, potentially affecting the cervical spine indirectly.

If your symptoms persist or worsen, I strongly recommend seeking professional medical advice. You may need to undergo tests such as cervical spine X-rays or MRI to rule out pathological factors like cervical spine abnormalities. Additionally, you may require blood tests to rule out other potential causes of your symptoms.

In the meantime, you can consider improving your lifestyle habits, such as taking short breaks to move and perform neck stretches every hour, adjusting the height of your workspace and chair to reduce neck strain. Getting adequate sleep, maintaining a healthy diet, and engaging in moderate exercise can also help alleviate symptoms. If your scoliosis is severe or worsening, you may benefit from seeing a physical therapist for specific treatments.

Please note that if your headaches suddenly worsen or if you experience new symptoms such as visual disturbances, severe nausea and vomiting, or altered consciousness, you should seek immediate medical attention.

74.

Hello, based on the symptoms you described, it is possible that your father may have a knee joint condition such as knee arthritis, particularly osteoarthritis, or a meniscus injury, among other possibilities. However, I cannot provide a definitive diagnosis as I am an artificial intelligence and have not conducted a face-to-face assessment of your father.

Osteoarthritis typically occurs in middle-aged and older individuals and is characterized by symptoms such as joint pain, swelling, and limited mobility. A meniscus injury, on the other hand, is often caused by twisting or overuse and may manifest with symptoms like knee pain, swelling, stiffness, and a feeling of instability while walking.

I recommend that your father consults a specialized orthopedic doctor at the hospital for a thorough evaluation. Further tests, such as X-rays or MRI scans, may be necessary to help diagnose the specific condition, and the doctor can provide tailored treatment options based on the diagnosis. If it is osteoarthritis, long-term use of anti-inflammatory medications, pain relievers, and appropriate physical therapy may be recommended. In the case of a meniscus injury, surgery may be necessary in some cases.

In daily life, your father can try to avoid walking uphill and downhill as much as possible to reduce stress on the knee joint. If he is overweight, weight control is advised as excess weight can increase stress on the knee joints. Engaging in low-impact exercises such as swimming can help maintain joint flexibility. If the pain is severe, he can consider using hot or cold packs to alleviate discomfort. Please note that these are general recommendations, and it's essential to follow the guidance of the doctor for treatment and daily management.

I hope the information I provided is helpful. If you have any more questions, please feel free to ask.

75.

Hello, the symptoms you described could be due to a meniscus injury or other knee joint issues, but it cannot be definitively determined based on the description alone. Twisting the knee during physical activity or sudden changes in direction can result in a meniscus injury. The meniscus is two C-shaped pieces of cartilage located within the knee joint, serving as cushions and stabilizers. Symptoms of a meniscus injury typically include knee pain, swelling, stiffness, difficulty walking, and the sensation of the knee "locking" temporarily (i.e., being unable to fully bend or straighten).

MRI (Magnetic Resonance Imaging) is a highly effective tool for diagnosing meniscus injuries and other issues related to cartilage, bones, or muscles. However, decisions regarding whether an MRI or other tests are needed and how to proceed with treatment should be made by a medical professional.

I recommend seeking medical attention as soon as possible, particularly from an orthopedic or sports medicine specialist. They can perform a physical examination and may recommend further imaging tests such as X-rays or an MRI. If it is indeed a meniscus injury, treatment may include rest, physical therapy, medication, and sometimes surgery.

In the meantime, it's advisable to avoid using the injured knee as much as possible before seeing a doctor. You can try using ice packs to reduce swelling and pain. I hope you recover quickly, and if you have more questions, please feel free to ask.

76.

Hello, based on your description, you may be experiencing "costochondritis" (also known as Tietze syndrome or costosternal syndrome), which is a less common condition that can cause pain at the joints between the sternum (breastbone) and the ribs. It is characterized by chest pain, especially when moving, yawning, taking deep breaths, coughing, or sneezing. Swelling in a specific area of the chest may also occur. However, it's important to rule out other conditions that could be causing these symptoms, such as heart or lung problems.

Yy advice is that you should seek medical attention as soon as possible, especially from an experienced sports medicine or orthopedic doctor. They can conduct a detailed physical examination and may recommend further diagnostic tests such as X-rays or an MRI. Additionally, if the pain is accompanied by shortness of breath, I strongly recommend seeking medical attention promptly, as this could be a sign of a heart issue.

Before seeing a doctor, you can avoid activities that may exacerbate the pain, use cold or warm compresses to alleviate discomfort, and consider over-the-counter pain relievers like ibuprofen or aspirin to help manage the pain.

77.

Hello, based on your description, it appears that you experienced patellar dislocation while playing basketball four years ago, and the clicking sound within the knee joint may be related to this. Specifically, there could be several reasons for this:

Patellar dislocation may have resulted in injuries to ligaments, tendons, or cartilage around the knee, which could lead to clicking sounds during knee movement.

There may be patellar cartilage damage or softening, causing the knee joint to produce clicking sounds during movement.

Patellar dislocation might have altered the way the knee joint moves or its tracking, which could also lead to clicking sounds.

However, the clicking sound itself doesn't necessarily indicate the presence of a medical condition. As long as it is not accompanied by symptoms such as pain, swelling, or difficulty in movement, there is generally no need to be overly concerned. Nevertheless, if the clicking is frequent, especially if it is accompanied by other symptoms like pain, swelling, instability, etc., you should consider seeing a doctor.

A doctor may perform physical examinations based on your symptoms and may recommend imaging tests like X-rays or MRIs to gain further insight into your condition. This will help determine if there is any structural damage and establish the best course of treatment.

Lastly, whether or not there is pain, maintaining regular physical activity and a healthy lifestyle is beneficial for joint health. If necessary, you can seek the assistance of a physical therapist to learn exercises that can improve joint function and stability.

78.

Hello, based on your description, you have stopped using dexamethasone (a type of glucocorticoid) for several days with a total dose of 55 milligrams. While long-term or high-dose use of glucocorticoids can increase the risk of avascular necrosis of the femoral head, in the case of short-term use and relatively low doses, this risk should be relatively low.

Avascular necrosis of the femoral head occurs when a portion of the bone tissue in the femoral head loses its blood supply, leading to its death. This typically occurs in individuals who use glucocorticoids long-term, abuse alcohol, or have certain medical conditions (such as fractures, fat embolism syndrome, etc.). People who use glucocorticoids in small doses for short periods, although there is some risk, generally face a low risk.

As for preventive measures, avoiding long-term or high-dose use of glucocorticoids is the most important step. Maintaining a healthy lifestyle, including a balanced diet, regular exercise, smoking cessation, and limiting alcohol intake, also contributes to bone health. Calcium and vitamin D supplements like Calcichew-D3 can help improve bone health, but they do not directly prevent avascular necrosis of the femoral head.

If you are concerned about the risk of avascular necrosis of the femoral head, it's advisable to consult with your doctor or pharmacist, as they can provide more specific recommendations based on your individual health status. If you experience any symptoms such as pain, swelling, or difficulty in movement, seek medical attention immediately.

79.

Regarding your father's condition, lumbar disc protrusion, clicking and pain in both knee joints, and the use of glucosamine sulfate capsules may be related to prolonged standing, walking, heavy physical work, or age-related degenerative joint conditions like osteoarthritis. Glucosamine sulfate capsules are typically used to treat joint pain and inflammation.

Concerning the slight pricking sensation in the buttocks when lifting the left leg and the swelling sensation in the left lower leg, along with a numb feeling in both lumbar muscles, this may be related to nerve compression, such as that caused by lumbar disc protrusion.

You mentioned the Patrick's test (FABER test) and the Lasegue test, which are typically used to assess hip and spinal conditions. However, the results of these tests need to be interpreted by a medical professional. If the left-sided Patrick's test causes mild discomfort, it may suggest issues with the hip joint or pelvis, but further examination is needed to confirm.

Given the current pandemic situation, if your father cannot see a doctor immediately, here are some suggestions that may help:

Maintain moderate activity: Prolonged sitting can exacerbate joint and back pain, so it's advisable to engage in moderate physical activity daily.

Gentle stretching exercises: These can help alleviate joint and muscle pain.

Balanced diet: Maintain a healthy diet with foods rich in vitamin D and calcium to support bone and joint health.

Use over-the-counter pain relievers appropriately: Consider using acetaminophen or non-steroidal anti-inflammatory drugs (NSAIDs) like ibuprofen under a doctor's guidance.

However, the most important thing is for your father to see a doctor for a comprehensive examination and assessment as soon as the pandemic conditions allow.

80.

Hello, your medical history includes avascular necrosis of the femoral head, which is a serious condition that can lead to joint pain and difficulty walking. Understanding the recent exacerbation of your left-sided pain is crucial, but it may require further medical evaluation.

Vitamin D and calcium are typically used to help maintain bone health, and DiCal is a supplement containing both of these components. Normally, they should not cause side effects or toxicity unless taken in excessively high doses. However, the symptoms you described (increased pain, particularly affecting walking and sleep) may not be caused by an overdose of vitamin D and calcium.

The worsening bone pain could be related to many factors, including the natural progression of arthritis, further deterioration of the femoral head, potential muscle or nerve damage, or even other unidentified medical conditions.

If your pain has reached a level where it significantly impacts your daily life, I strongly recommend contacting a doctor as soon as possible to discuss your symptoms and potential treatment options. Before getting guidance from a doctor, you may need to stop taking the vitamin D and calcium supplements to see if the pain improves. If the pain persists, it may be a signal of disease progression or another issue that requires immediate medical attention.

81.

Hello, your mother's symptoms may be related to knee joint issues, possibly caused by osteoarthritis (also known as degenerative joint disease) or other related conditions. Osteoarthritis is a common joint disorder that often leads to pain, stiffness, and difficulty in movement.

When going to the hospital, here are some tests that may be conducted:

Physical examination: The doctor may observe if there is swelling, redness, or tenderness in your mother's knee and assess the range of motion of the knee joint.

Imaging tests: These may include X-rays, MRI, or CT scans. These tests can help the doctor understand the detailed structure of the knee joint and whether there are issues such as arthritis, cartilage wear, or joint fluid accumulation.

Blood tests: While most causes of knee joint pain cannot be detected through blood tests, they can help rule out other potential causes, such as rheumatoid arthritis or infection.

Joint aspiration: In certain cases, if there is a significant buildup of fluid in the knee joint, the doctor may perform joint aspiration to remove joint fluid for further examination.

Additionally, it's a good idea to have a detailed medical history for your mother, including the onset of pain, the location of pain, the severity of pain, and any factors that may alleviate or exacerbate the pain. This information can be very helpful for the doctor.

Lastly, it's important to note that even mild knee joint pain should be addressed promptly to prevent the condition from worsening.

82.

Hello, coccyx pain (also known as coccydynia) is a relatively common condition that may be associated with prolonged sitting on hard chairs. It can occur due to prolonged pressure on the coccyx, leading to fatigue or tension in the surrounding muscles and ligaments. Discomfort when sitting is a common symptom of this condition.

In most cases, coccyx pain can be self-managed. Here are some suggestions that may help you:

Adjust seating and cushions: Try to maintain proper posture and avoid sitting on hard chairs for extended periods. You can use cushions or coccyx cushions (which have a central cutout to relieve coccyx pressure) for added comfort.

Take regular breaks and move: If your work or daily habits require prolonged sitting, remember to stand up and move around or perform simple stretching exercises every so often (e.g., every hour).

Warm compress: Applying a warm compress can help alleviate muscle tension and pain. You can try placing a hot water bottle on the painful area.

Over-the-counter medications: Over-the-counter pain relievers like acetaminophen or non-steroidal anti-inflammatory drugs (NSAIDs) such as ibuprofen can help relieve pain. However, if you have any known medication allergies or are taking other medications, consult a doctor before use.

However, if the pain persists or worsens, or if you experience other symptoms such as difficulty with bowel movements, urinary incontinence, or weakness in the lower limbs, you should seek medical attention immediately, as these may be symptoms of a more serious condition.
