# Supplementary material for: Quality and Dependability of ChatGPT and DingXiangYuan Forums for Remote Orthopedic Consultations: Comparative Analysis
Source: J Med Internet Res. 2024 Mar 14;26:e50882. doi: 10.2196/50882 (PMC10979330; doi:10.2196/50882)
Supplement: Multimedia Appendix 5 [file jmir_v26i1e50882_app5.docx]

**Table S1**. Consistent evaluation of Fleiss kappa among 3 raters.

|  | Item | [Fleiss kappa](http://www.baidu.com/link?url=8gk3WGJG5oEUITvdVcbcoAhukuEhkXPUwc0WhKhCDr9tXiqhDxRrIdMOVOvAEO_x3rrkWapWcFrwqjJ_RfVwuatgSfUQgYHFA8jXtFmzeim) | Interpretation | *P* value |
| --- | --- | --- | --- | --- |
| Control Group | Logical reasoning | .28 | Fair agreement | <.001 |
|  | Internal information | .37 | Fair agreement | <.001 |
|  | External information | .33 | Fair agreement | <.001 |
|  | Guiding function | .71 | Substantial agreement | <.001 |
|  | Therapeutic effect | .39 | Fair agreement | <.001 |
|  | Medical knowledge popularisation education | .39 | Fair agreement | <.001 |
|  | Overall satisfaction | .39 | Fair agreement | <.001 |
| ChatGPT Group | Logical reasoning | .44 | Moderate agreement | <.001 |
|  | Internal information | .29 | Fair agreement | <.001 |
|  | External information | .29 | Fair agreement | <.001 |
|  | Guiding function | .46 | Moderate agreement | <.001 |
|  | Therapeutic effect | .43 | Moderate agreement | <.001 |
|  | Medical knowledge popularisation education | .41 | Moderate agreement | <.001 |
|  | Overall satisfaction | .28 | Fair agreement | <.001 |
| Consistent interpretation of Fleiss kappa values: Poor agreement (<.01); Slight agreement (.01-.20); Fair agreement (.21-.40); Moderate agreement (.41-.60); Substantial agreement (.61-.80); Almost perfect agreement(.81-1.00); | | | | |
