# Supplementary material for: Quality and Dependability of ChatGPT and DingXiangYuan Forums for Remote Orthopedic Consultations: Comparative Analysis
Source: J Med Internet Res. 2024 Mar 14;26:e50882. doi: 10.2196/50882 (PMC10979330; doi:10.2196/50882)
Supplement: Multimedia Appendix 6 [file jmir_v26i1e50882_app6.docx]

**Question 1**

Good day, Doctor. My 60-year-old mother discovered a bulge on her wrist this evening that feels soft and pliable like a water sac. There is no history of mosquito bites, and there is no current particular discomfort other than a painful arm; thus, I would want to know if there is a potential need for medicine.

**Answer on the "DOCTOR DINGXIANG" forum**

I believe there may be two causes for the localised swelling of the patient's wrist, based on the facts you supplied. A tendon sheath cyst is the first option; this indicates that the tendon in his wrist and the tendon sheath on its surface have been continuously strained, resulting in local inflammation, which will cause local fluid buildup, and eventually this cyst will emerge. For instance, it is easy to see this circumstance since the patient often puts in more effort, particularly with their wrists being more active. The patient's present state may fit this ailment since, typically, there will be no noticeable signs at first, such as the cyst, but there may be some wrist discomfort or swelling. In this situation, it is advised to take the patient to the orthopaedics department of the hospital tomorrow to have a look. One is to ask the doctor to examine the little bump, and the other is to do a local ultrasound to confirm that the lump is what I believe it to be. If it's a cyst, there's no need to panic since there are no particular treatments that can be done for ganglion cysts with no symptoms, but you should take care to rest well to prevent the cyst from growing further. That requires him to purchase a wrist brace for about a month. Each day, a local heat compress should be used together with a local application of Futalin ointment to the skin to reduce local inflammation and allow for the gradual absorption of local fluid. Perhaps this cyst will gradually go away, making the issue less serious and allowing you to stop worrying. Even if the cyst is too large to be absorbed or your mother doesn't get enough sleep, it will continue to grow. If the cyst subsequently interferes with your mother's wrist movements or causes unpleasant symptoms, we can do a little procedure to remove the cyst, and everything will return to normal. However, the cyst is not particularly huge at this time, and there are no noticeable pain signs, so I believe I may avoid surgery and get by with conservative therapy.

The second option is that this swelling is a subcutaneous lipoma rather than a cyst. Older women have rather loose soft tissue beneath their skin, and if a little heavier woman is predisposed to lipoma, this area in the wrist may also manifest. In this case, a visit to the hospital is required as well as an ultrasound examination; however, if a lipoma is detected, no more care is required. There is no need for surgery since a little lipoma has no evident symptoms, so simply keep an eye on it. It doesn't really matter as long as it doesn't keep growing. You shouldn't be concerned since the lipoma is a benign tumour and won't have a significant effect.

Logical reasoning: 5

Internal information: 5

External information: 5

Guiding function: 5

Therapeutic effect: 5

Medical knowledge popularisation education: 5

Overall satisfaction: 10

**ChatGPT’s answer**

Both a synovial cyst and a tendon sheath cyst are typical benign tumours that are often fluid-filled, soft to the touch, and may fluctuate in size, which may explain your mother's complaints.

1. Synovial cyst: The bursa, a pouch close to the tendons and joints that is filled with synovial fluid to reduce friction and promote easy joint movement, is one kind of synovial cyst. Cysts may develop as a result of synovial fluid accumulating in the bursa after injury or inflammation.

2. Tendon sheath cyst: The tendon sheath, a membrane that encircles the tendon and also includes a lubricating fluid to aid with tendon sliding, is a condition that may occur. A tendon cyst may develop if the tendon sheath is irritated or damaged.

In general, if these two cysts are painless and do not interfere with function, they may be left alone for a while to see whether they go away on their own. To lessen discomfort and the cyst's growth, avoid overusing the wrist. Consider wearing wrist protectors, reducing wrist motion, or using over-the-counter non-steroidal anti-inflammatory medicines (like ibuprofen) for pain relief if it aches or interferes with function.

You may need to see a doctor if the cyst continues, enlarges, or limits the use of your wrist. The cyst may be removed surgically or by aspiration at the doctor's discretion.

Although the likelihood of the aforementioned possibilities is higher, it should be noted that this is merely a rough guess based on the symptoms you've mentioned and is not a diagnosis. You should make an appointment with your mother's doctor as soon as possible for a thorough examination, an accurate diagnosis, and treatment if the cyst continues, expands, is painful, or exhibits any new symptoms.

Logical reasoning: 5

Internal information: 5

External information: 5

Guiding function: 5

Therapeutic effect: 5

Medical knowledge popularisation education: 5

Overall satisfaction: 10

**Explanation**: The responses provided by both the manual and ChatGPT4.0 methods are deemed satisfactory. The responses adequately incorporate the details provided in the patient's enquiries, and the elucidation of the patient's condition is straightforward and uncomplicated, devoid of specialised terminology. Concurrently, both diagnoses and treatment recommendations were extremely precise. Perfect points for both responses.

**Question 2**

Hello, My right foot started hurting six months ago after I had an injury. My body was not X-rayed. It progressively improved after being sprayed with Yunnan Baiyao on the back. Nothing odd about looks; no recent injuries; just a touch warm. What is the issue?

**Answer on the "DOCTOR DINGXIANG" forum**

Hello, gout is most likely based on your description. The condition known as gout is brought on by a high amount of uric acid in the blood, which causes uric acid crystals to build up in the joints and produce severe inflammatory assaults in and around the joints. Alcohol use is a typical gout risk factor.

Patients with gout often experience sudden, intense pain in one or more joints, more commonly at night, as well as joint redness, swelling, an increase in skin temperature, red, tight, glossy skin, and other symptoms.

 It is advised that you visit a hospital to have your uric acid checked. Additionally, since your current pain is obvious, you can take oral non-steroidal anti-inflammatory medications like celecoxib, diclofenac sodium, etc., apply voltaren ointment locally, drink more water, and urinate more frequently.

 Pay attention to your diet.

Continue to drink water and stay away from alcohol, sweet beverages, and high-purine animal meals such as offal and shellfish.

Drink in moderation, particularly beer.

Limit your consumption of foods rich in purines. Animal offal, pig, cattle, lamb, shellfish, anchovies, sardines, tuna, etc. are typical meals with high purine content.

Avoid beverages that are high in fructose.

More than 2000 ml of water should be consumed daily.

Boost your consumption of fresh veggies.

Logical reasoning: 5

Internal information: 5

External information: 5

Guiding function: 5

Therapeutic effect: 5

Medical knowledge popularisation education: 4

Overall satisfaction: 9

**ChatGPT’s answer**

There might be a number of causes for your disease, including:

1. Your bones could have sustained a new injury. Even though there isn't much memory impairment, certain actions, particularly small sprains that you may not be aware of after consuming alcohol, might be harmful.

2. Past injuries may not have entirely healed. The original injury may not completely heal, and you may have discomfort if your ankle does not get the right care and enough healing time.

3. You might be inflamed. After your ankle is hurt, the tissues around it may become inflamed, particularly if your ankle doesn't receive enough rest and rehabilitation time. Ankle heat, soreness, and even swelling may all be brought on by inflammation.

In this instance, I advise that you get medical help as soon as you can, particularly if your symptoms intensify or continue. To more precisely assess the kind and severity of the issue, the doctor could recommend X-rays or other imaging procedures. Additionally, they could suggest certain therapies like rest, physical therapy, or even medication.

Logical reasoning: 2

Internal information: 1

External information: 2

Guiding function: 3

Therapeutic effect: 1

Medical knowledge popularisation education: 1

Overall satisfaction: 2

**Explanation**: Answers provided manually to this query are more precise. The artificial responses presented here effectively convey the crucial stimulus that is alcohol consumption as expressed in the patient's queries. This emphasises the benefits of manual responses, namely that not only does medical treatment require a high level of professional knowledge, but clinical experience is also crucial. The causes of gout, however, are inadequately communicated to patients. ChatGPT4.0's response to this query appears to lack comprehension of the fundamental aspect of diagnosis, as it merely offers conjecture regarding the site of the patient's ailment. Neglecting to utilise "internal information" supplied by patients in an appropriate manner. The absence of a precise diagnosis renders "external information" non-existent. In addition to the diagnosis and treatment plan receiving an inadequate grade, the overall satisfaction evaluation is also a failing grade, despite the fact that the disease's location is well-known but insufficiently described.
